# Genetic variation and genetic control of intraspikelet differences in grain weight and seed dormancy in wild and domesticated emmer wheats

**DOI:** 10.1270/jsbbs.21060

**Published:** 2022-06-29

**Authors:** Shoji Ohta

**Affiliations:** 1 Professor emeritus, Department of Bioscience and Biotechnology, Fukui Prefectural University, 4-1-1 Matsuoka-Kenjojima, Eiheiji, Yoshida, Fukui 910-1195, Japan

**Keywords:** *Triticum turgidum*, wild emmer wheat, seed dormancy, grain weight, genetic diversity, domestication, preharvest sprouting

## Abstract

Seed dormancy, a vital strategy for wild plant species to adapt to an unpredictable environment in their natural habitats, was eliminated from cereals during the domestication process. Intraspikelet differences in grain size and seed dormancy have been observed in wild emmer wheat. To elucidate the genetic variation of these intraspikelet differences and to determine their genetic control, grain weight ratio (first florets/second florets) (GWR), germination rate, and germination index (GI) were analyzed in 67 wild and 82 domesticated emmer wheat accessions, as well as F_1_ hybrids, F_2_ populations, and F_3_–F_6_ populations derived from reciprocal crosses between wild and domesticated lines. Only the grains on the first florets of two-grained spikelets in wild accessions had varying degrees of dormancy with GI ranging from 0 to 1, which positively correlated with their GWR. This implies that wild emmer populations comprised genotypes with varying degrees of dormancy, including nondormant genotypes. According to segregations observed in F_2_ populations, the intraspikelet grain weight difference was controlled by two independently inherited loci. Furthermore, low-GWR populations with low or high GI values could be selected in F_5_ and F_6_ generations, implying that the major loci associated with dormancy might be independent of intraspikelet grain weight difference.

## Introduction

Seed dormancy and the formation of soil seed banks are crucial strategies for wild grass species to survive in natural habitats under unpredictable environmental conditions (Simpson 1990). Previous studies on seed dormancy in wheat and its wild relatives attempted to elucidate the following three fundamental or applied topics.

The first topic is to develop favorable varieties, which are tolerant to preharvest sprouting (PHS). To that end, numerous varieties of domesticated wheats, particularly of common wheat, have been screened, and their seed dormancy was examined prior to after-ripening. In these studies, PHS-controlling alleles and QTLs were successfully identified (Kato *et al.* 2001, Kulwal *et al.* 2010, Mares *et al.* 2005; also see Nakamura 2018 for review).

The second topic is to elucidate the adaptation strategies of wild wheat relatives to different environments, such as a group of plant taxa adapted to drylands (Gutterman 2002). To address this challenge, the germination capacity of seeds collected from natural populations under different geographic, climate, and/or soil conditions was evaluated (Volis 2016). Several annual plant species living in drylands have evolved “phenotypic plasticity of seed germination”, and seeds from a single mother plant might have varying degrees of germinability (Gutterman 2002). Intraspikelet differences in grain size and seed dormancy have been observed in wild Mediterranean and Southwest Asian species from the Poaceae family, whose seed dispersal units contain more than a single grain, such as *Triticum dicoccoides* (Cook 1913, Harlan 1992, Nave *et al.* 2016), *T. monococcum* (Ohta 2009), *Aegilops geniculata* (Datta *et al.* 1970, Marañon 1989, Niwa *et al.* 2005), *Ae. neglecta* (Marañon 1989, Niwa *et al.* 2005), *Ae. kotschyi* (Wurzburger and Leshem 1967), *Ae. triuncialis* (Dyer 2004, Marañon 1989), *Ae. umbellulata* (Niwa *et al.* 2005), *Dasypyrum villosum* (De Pace *et al.* 1994, 2011), and *Avena ludoviciana* (Morgan and Berrie 1970). In spikelets of these grass species, the first (or lower) florets bear smaller and dormant grains, while the second (or upper) florets bear larger and nondormant grains.

The third and last topic is to understand the genetic changes that occurred during the domestication process from wild to domesticated wheat. Besides the development of tough rachises and the increase in grain size, one of the most distinct genetic changes caused by wheat domestication is the decrease or elimination of seed dormancy (Abbo *et al.* 2014, Fuller 2007, Harlan 1992, Zohary and Hopf 2000). Changes in the two first-mentioned morphological characteristics during the domestication process can be tracked from carbonized remains excavated from Epipaleolithic and early Neolithic sites in Southwest Asia (Fuller 2007, Tanno and Willcox 2006, Willcox 2004). Regarding seed dormancy, Fuller (2007) suggested that its loss was selected simply via cultivation and sowing from harvested seeds, because poorly germinated seeds had no effect on harvest yield. Seed dormancy, however, is a physiological characteristic that cannot evaluate from archaeological remains. To gain an insight into the genetic changes associated with seed dormancy that occurred during the domestication process, it is necessary to evaluate and compare the genetic variation in seed dormancy in living accessions of both wild and domesticated wheats, as well as to elucidate its genetic control. However, no wide-scale comparative study on seed germination of wild or domesticated emmer wheat has been conducted to date (Abbo *et al.* 2014).

This study comprised two experiments. The first experiment aimed to survey the genetic variation in intraspikelet differences in grain size and seed dormancy in both wild and domesticated emmer wheats. The second experiment elucidated the genetic control on intraspikelet differences.

## Materials and Methods

### Plant materials

For the first experiment, 67 accessions of wild emmer wheat (*Triticum turgidum* ssp. *dicoccoides* (Asch. & Graebn.) Thell.) and 82 accessions of non-free-threshing, or hulled, domesticated emmer wheat (*T. turgidum* ssp. *dicoccum* (Schrank ex Schübl.) Thell.) were used (Supplemental Table 1) (Furuta and Ohta 1993). All accessions are preserved at Kyoto University and managed by the National BioResource Project–Wheat, Japan. Wild emmer accessions were obtained from almost the whole distribution area of the subspecies, and their collection localities were divided into eight groups based on their geographical regions: Iran, Iraq (Northeastern), Iraq (Northern), Israel (Upper Jordan Valley and Mt. Hermon), Israel (Judean Mountains and foothills), Syria, Turkey (Karacadağ region near Diyarbakir), and Turkey (Karadağ region near Gaziantep) (Supplemental Table 1). Based on the results from the first experiment and owing to synchronized flowering, a wild accession from East Anatolia in Turkey with distinct intraspikelet differences in grain weight and seed dormancy (Accession No. W43) and a domesticated accession from Ethiopia (Accession No. D26) were selected as parental lines for the second experiment involving crossing. Grain weight and germination capacity of the parental lines, F_1_ grains from the maternal plants, and F_1_ hybrid plants obtained from their reciprocal crosses, F_2_ populations, and successive F_3_–F_6_ populations were analyzed.

### Plant cultivation and grain group preparation

For the first experiment, 60 wild and 37 domesticated accessions, and 66 wild and 69 domesticated accessions were sown in sterilized soil in December 2008 and 2009, respectively. About a month after sowing, two plants per accession were transplanted into 20-cm-diameter pots and grown in an unheated greenhouse at Fukui Prefectural University (FPU) under natural daylight conditions for both growing seasons. Additionally, 57 wild and 35 domesticated accessions were sown in October 2013. In November, a plant per accession was transplanted into an experimental field at FPU with 90 cm interspace between rows and 30 cm between individuals. Also, two plants per accession were transplanted into 20-cm-diameter pots in an unheated greenhouse. Throughout the growing season, they were grown under natural daylight conditions.

For the second experiment, the wild and domesticated parental lines were reciprocally crossed. Their F_1_ hybrids and F_2_ populations were transplanted into 20-cm-diameter pots and grown in an unheated greenhouse at FPU with their parental lines during the 2011–2012 growing season. Both F_1_ and F_2_ plants were grown again during the 2012–2013 growing season at the FPU experimental field under the same conditions as in the first experiment. The F_3_–F_6_ populations derived from an F_2_ plant were also grown in successive growing seasons at the FPU experimental field.

Five spikes from each plant were bagged before flowering to allow self-pollination and were harvested at ripening. They were dried at room temperature before being used for the germination experiments. Most spikelets consist of two fertile florets in emmer wheat, the first floret occupies a lower position, and the second floret is placed in an upper position. The third floret, which is typically rudimental and sterile, is in the topmost position. Spikes were threshed by hand with identifying the positions of florets in spikelets, and husk-free grains were divided into the following grain groups: (1) first floret grains from two-grained spikelets, (2) second floret grains from two-grained spikelets, (3) first floret grains from one-grained spikelets, and (4) second floret grains from one-grained spikelets. Intact grains belonging to groups 1–3 were used for the experiments since the grains belonging to group 4 were few and insufficient for the experiments.

### Grain weight measurements

Grain weight was measured using an electronic analytical scale. In this study, this parameter was used instead of the three elements of the grain size (length, width, and thickness) since measuring weight is considerably easier than measuring grain size and because it is correlated to grain volume when the density of each grain is uniform. In the first experiment, the weight of 20 grains from the three grain groups of each plant was individually measured, and the mean values and standard errors were calculated for the experiment performed in 2009. The weight of 10 and 50 grains from each grain group was measured in the 2010 and 2014 experiments, respectively. In the second experiment, 25 grains or as many grains as available from the three grain groups in parental lines, their reciprocal F_1_ hybrids, F_2_ populations, and F_3_–F_6_ populations were measured, and the mean values and standard errors of one-grain weight were calculated. In 2013, two replicates, both including 25 grains, were measured, and the average values were used for comparisons. Additionally, the weight of each F_1_ grain obtained from the maternal parents after the reciprocal crossing was measured in 2012.

In both the first and second experiments, the mean one-grain weight of each grain group and the grain weight ratio (GWR; the mean one-grain weight of the first florets/the mean one-grain weight of the second florets) within two-grained spikelets were used for comparisons. Grain weight *ratio* was adopted instead of grain weight *difference* for the current analysis to exclude possible effects from the variation in grain weight among accessions that could be controlled by genetic factors other than those related to intraspikelet difference in grain weight. For comparison of mean one-grain weight and GWR among groups, parametric statistic tests, including *t*-test, paired *t*-test, repeated measures analysis of variance (Holm method), Tukey–Kramer test, and Pearson’s correlation coefficients, were applied.

### Germination tests

Grains were placed on filter paper in 9-cm-diameter Petri dishes, and distilled water was poured in to soak the lower half of each grain. Subsequently, all Petri dishes were put in an incubator set at 20ºC in the dark. Germinated grains were counted every 12 h for the first three days and, afterward, every 24 h until the eighth or tenth day after sowing. The duration of the observation was determined based on the results of the first experiment. Nongerminated grains covered with fungi during the observation were deemed unviable and were excluded from counting (Baskin and Baskin 2001).

For the first experiment, germination tests were conducted twice in 2009, with a two-month interval from September 6 to November 6. In 2010 and 2014, grains were sown on September 19 and September 9, respectively. For the second experiment, grains were sown in either late August or early September. Two variables, germination rate (GR) and germination index (GI), were used for comparisons. GR represents the ratio of the total number of grains germinated during the observation to the total number of grains sown, and GI was calculated according to the following formula: $\sum_{i=1}^{d}\left\{\left(1-\frac{i-1}{d}\right)\times \frac{n_{i}}{N}\right\}$ (where *N* is the number of grains sown, *d* is the duration of observation in days, and *n_i_* is the number of grains germinated on the *i*^th^ day after sowing). Both variables ranged from 0 to 1. For the comparisons of GR and GI among groups, nonparametric statistic tests, including Friedman test, Mann–Whitney *U* test, Wilcoxon signed-rank test, and Spearman’s rank correlation coefficient, were applied.

## Results

### Variation in grain weight and GWR values

**Fig. 1.**
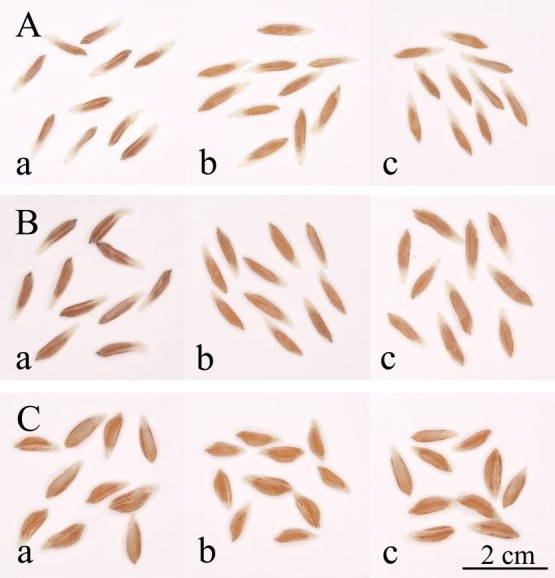
Grain size difference among the three grain groups in wild and domesticated emmer wheat harvested in 2009. A: wild emmer wheat W16 (GWR = 0.765), B: wild emmer wheat W17 (GWR = 0.850), and C: domesticated emmer wheat D82 (GWR = 1.065). The first and second floret grains of two-grained spikelets, as well as the first floret grains of one-grained spikelets, are indicated with a, b, and c, respectively.

**Fig. 2.**
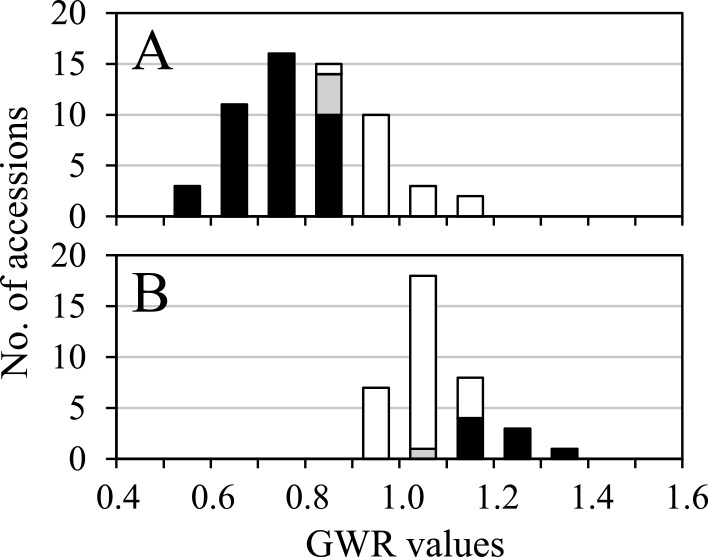
The frequency distribution of the GWR values in the 60 wild (A) and 37 domesticated (B) emmer wheat accessions measured in 2009. The black and gray columns represent accessions in which the mean one-grain weights of the first and second floret grains significantly differ at *p* = 0.01 and *p* = 0.05, respectively, and the white columns represent those in which the mean one-grain weights did not significantly differ between the first and second florets.

**Fig. 3.**
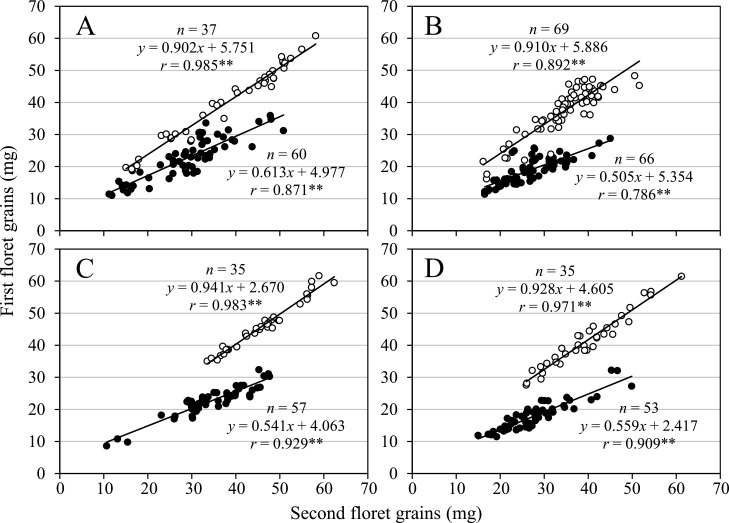
Scatter diagrams showing correlations between the one-grain weight of the first and second floret grains in two-grained spikelets in wild (solid circles) and domesticated (open circles) emmer wheat. A: harvested in 2009, B: harvested in 2010, C: grown at an experimental field and harvested in 2014, and D: grown in an unheated greenhouse and harvested in 2014.

Out of 60 wild emmer wheat accessions whose grain weight was measured in 2009, 44 (73.3%) displayed significantly lower one-grain weights of the first floret grains than those of the second floret grains in two-grained spikelets, whereas they were not significantly different in the remaining 16 (26.7%) accessions (Figs. 1A, 1B, 2A, Supplemental Table 2). The mean one-grain weight of the first floret grains across the 60 wild accessions was significantly lower than that of the second floret grains (Table 1). In contrast, among 37 domesticated accessions, the one-grain weight of the first and second floret grains in two-grained spikelets did not significantly differ in 28 (75.7%) accessions, and the former was significantly higher than the latter in nine accessions (Figs. 1C, 2B, Supplemental Table 2). The mean one-grain weight of the first floret grains of the 37 domesticated accessions was significantly higher than that of the second floret grains. The mean GWR values of wild accessions were significantly lower than those of the domesticated accessions (Table 1). Similar results were obtained throughout the first experiment from 2009 to 2014 (Table 1). The mean one-grain weight varied significantly among accessions, with the first and second floret grains showing highly significant correlations in this value in both wild and domesticated accessions (Fig. 3, Supplemental Tables 2, 3). During the first experiment, gradients of the regression lines in wild accessions, ranging from 0.505 to 0.613, were almost half of the gradients in domesticated accessions, ranging from 0.902 to 0.941 (Fig. 3).

In wild accessions, the mean one-grain weight of the first floret grains in one-grained spikelets was not significantly different from that of the second floret grains of two-grained spikelets recorded in 2009 or was intermediate between the values of the first and second floret grains in two-grained spikelets scored in 2010 and 2014 (Table 2). In domesticated accessions, the mean one-grain weight of one-grained spikelets was significantly lower than that of two-grained spikelets (Table 2).

Except for the GWR values recorded in domesticated accessions in 2009/2010 and wild accessions in 2014, the mean one-grain weight and GWR values differed significantly between harvest years and growing environments (Table 3). Moreover, these two variables showed significant positive correlations between harvest years and growing environments, except for the GWR values of domesticated accessions scored in 2009/2010 (Table 3, Supplemental Fig. 1A, 1B).

GWR values varied considerably within each geographical region, and no significant differences in GWR values were discovered among the eight geographical regions at *p* = 0.01 (Supplemental Table 5).

### Variation in GR and GI values

**Fig. 4.**
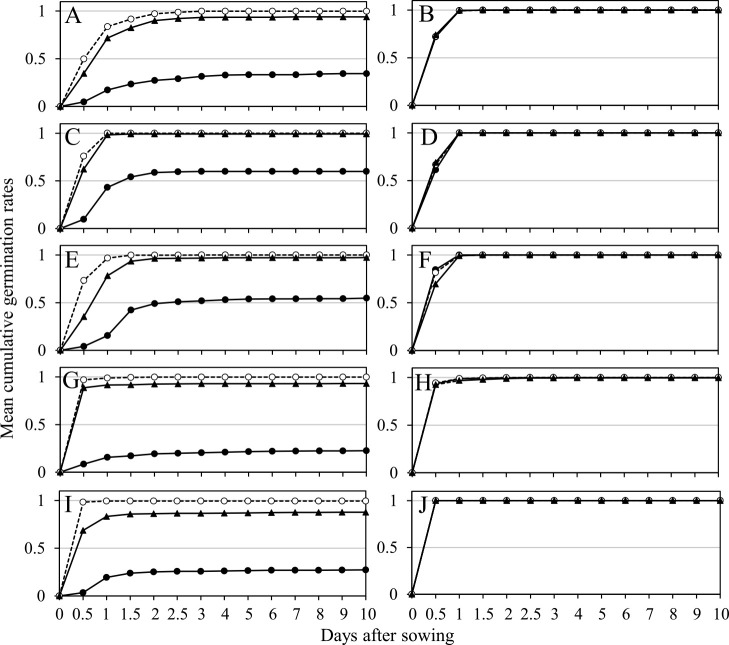
Time courses over ten days from sowing of the mean cumulative germination rates of the first (solid circles and lines) and second (open circles and broken lines) floret grains of two-grained spikelets, and the first floret grains of one-grained spikelets (solid triangles and lines). The left column (A, C, E, G, and I) and right column (B, D, F, H, and J) are wild and domesticated emmer wheat, respectively. A and B: sown in September 2009, C and D: sown in November 2009, E and F: sown in 2010, G and H: grown at an experimental field and sown in September 2014, and I and J: grown in an unheated greenhouse and sown in September 2014.

**Fig. 5.**
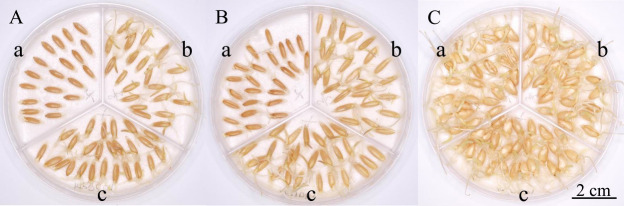
Germination 1.5 days after sowing under 20ºC and continuous dark conditions in wild and domesticated emmer wheat grown in an unheated greenhouse and harvested in 2014. A: wild emmer wheat (W05), B: wild emmer wheat (W10), and C: domesticated emmer wheat (D09). The first and second floret grains of two-grained spikelets, as well as the first floret grains of one-grained spikelets, are indicated with a, b, and c, respectively, in each Petri dish.

**Fig. 6.**
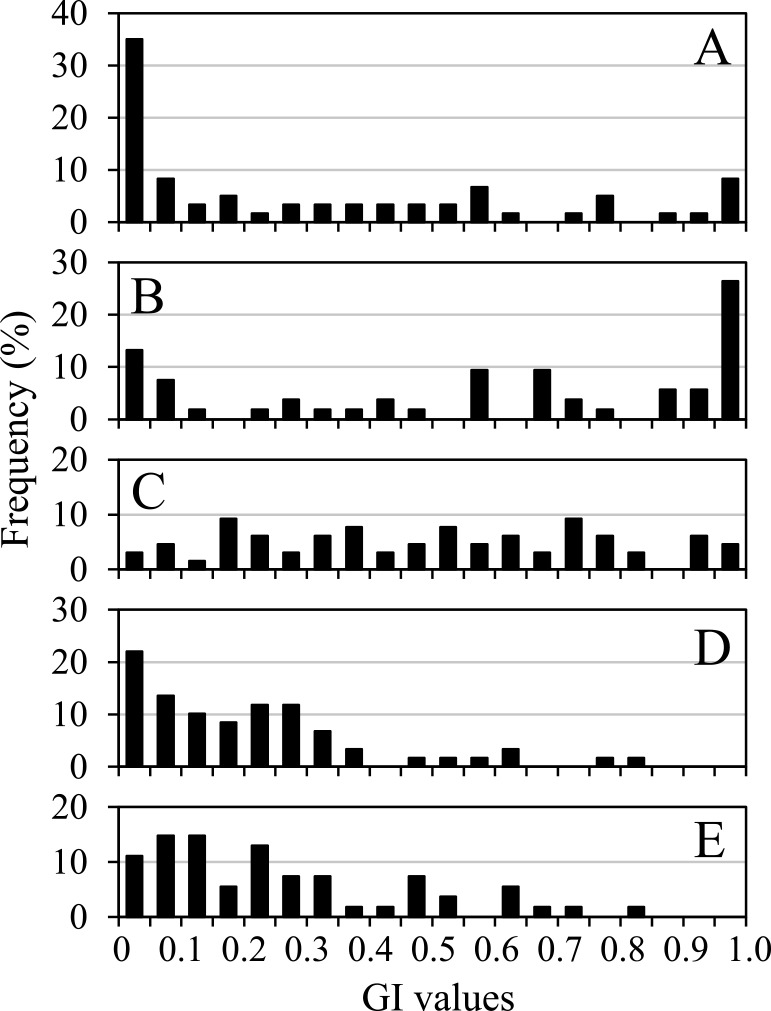
Frequency distributions of the GI values of the first floret grains of two-grained spikelets in wild emmer wheat. A: sown in September 2009, B: sown in November 2009, C: sown in September 2010, D: grown at an experimental field and sown in September 2014, and E: grown in an unheated greenhouse and sown in September 2014.

Grains from the grain groups of domesticated accessions germinated promptly after sowing, and their GR values a day after sowing were almost 1.00, regardless of harvest years or growing environments (Figs. 4B, 4D, 4F, 4H, 4J, 5C). Their GI values were almost 1.00 across all accessions (Supplemental Table 4) and did not significantly differ among the three grain groups (Table 4). In wild accessions, the second floret grains of two-grained spikelets and the first floret grains of one-grained spikelets germinated rapidly and showed high GR values (Figs. 4A, 4C, 4E, 4G, 4I, 5A, 5B). Their GI did not differ significantly, as recorded in 2009 and the field growing environment in 2014, however, the GI of one-grained spikelets was slightly lower than that of the second floret grains in the observation in 2010 and the greenhouse environment in 2014 (Table 4). GI values of the second floret grains in wild accessions did not significantly differ from those scored in domesticated accessions in two observations. In contrast, the first floret grains of two-grained spikelets in wild accessions germinated slowly, and their mean GR values ten days after sowing were lower than those of other grain groups (Fig. 4A, 4C, 4E, 4G, 4I). Their GI values were significantly lower than those recorded in the other two grain groups in wild accessions and lower than those of the first floret grains in domesticated accessions across all observations (Table 4). Additionally, the GI values of the first floret grains in two-grained spikelets in wild accessions varied significantly: 0–1 in 2009 and 2010, and 0–0.83 and 0–0.81 in 2014 (Fig. 6, Supplemental Table 4).

GI values varied widely within each geographical region, and no significant difference in GI values was discovered among the eight geographical regions at *p* = 0.01, except for comparisons between Turkey (Karadağ region near Gaziantep) and two Iraqi regions under greenhouse condition in 2014 (Supplemental Table 5).

### Comparison of GI values between sowing months, harvest years, and growing environments

GI values of each grain group were statistically compared across sowing months, harvest years, and growing environments (Table 5). In wild accessions, GI values of all grain groups recorded in November 2009 were significantly higher than those observed in September 2009. In 46 (86.8%) of the 53 wild accessions studied, the GI values of the first floret grains of two-grained spikelets in November were higher than those in September (Supplemental Table 4). Grains harvested from wild accessions in 2010 had significantly higher GI values than those harvested in 2009. The GI value of the first floret grains of two-grained spikelets in wild greenhouse-grown accessions was significantly higher than that of the accessions grown in the experimental field in 2014, whereas the GI values of the other two grain groups were very high and did not differ significantly between growing environments. The GI values of the first floret grains in two-grained spikelets scored across the wild accessions exhibited significant positive correlations in all three comparisons (Table 5, Supplemental Fig. 1C–1E). Among the domesticated accessions, the GI values of all grain groups were extremely high, with no significant difference detected, except for the comparison of growing environments.

### Correlations between grain weight and GI values

In wild accessions, GWR and GI values of the first floret grains in two-grained spikelets exhibited significant positive correlations in all observations in 2009, 2010, and 2014, however, there was no significant positive correlation between one-grain weights and GI values (Table 6, Supplemental Fig. 2).

### Grain weights in F_1_ grains, F_1_ hybrids, and F_2_ populations

**Fig. 7.**
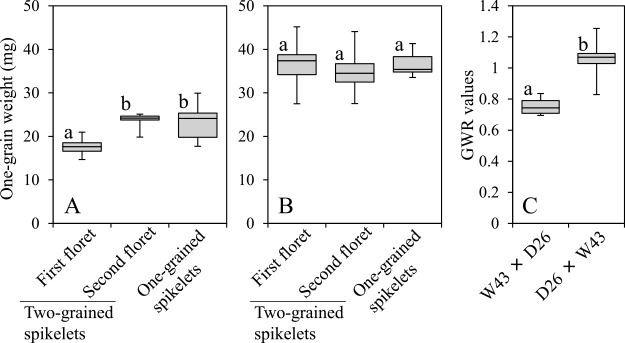
Box plots showing comparisons of medians, the first quartiles, and ranges in the F_1_ grains obtained from reciprocal crossing between wild (W43) and domesticated (D26) parental lines. A and B: one-grain weight of the first and second floret grains in two-grained spikelets, and the first floret grains in one-grained spikelets obtained from the crossing of A: W43 × D26, and B: D26 × W43, and C: GWR values of the first and second floret grains in two-grained spikelets obtained from the reciprocal crossing. Different small letters indicate that the means significantly differ from each other at *p* = 0.01 by Tukey–Kramer test (A and B) or *t*-test (C).

The mean one-grain weights of F_1_ grains of the first and second florets in nine two-grained spikelets and 14 grains of one-grained spikelets obtained from the cross with the wild line W43 as the maternal parent were 17.71 ± 0.63, 23.50 ± 0.63, and 23.05 ± 0.96 mg, respectively (Supplemental Table 6), with the first significantly lower than the other two (Fig. 7A). In contrast, no significant difference was discovered among one-grain weights of the three floret groups derived from the cross with the domesticated line D26 as the maternal parent (Fig. 7B). Thus, the mean GWR of the first and second floret grains in two-grained spikelets derived from the cross W43 × D26 was significantly lower than that of the cross D26 × W43 (Fig. 7C). These results indicate that GWR values of F_1_ grains are controlled by the genotypes of maternal plants while embryos of F_1_ grains obtained from reciprocal crossing have the same genotype heterozygous for alleles derived from wild and domesticated parental lines.

**Fig. 8.**
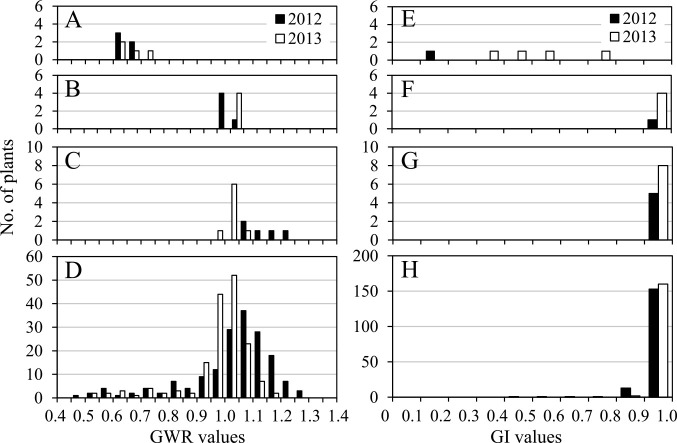
Frequency distributions of GWR values (A–D) and GI values (E–H) in wild (A and E) and domesticated (B and F) parental lines, their F_1_ hybrids (C and G), and their F_2_ populations (D and H) observed in 2012 and 2013. Data from reciprocal cross combinations are pooled because there was no significant difference between them in F_1_ hybrids and F_2_ populations.

Fig. 8A–8D show the frequency distributions of GWR values recorded across the wild and domesticated parental lines, their F_1_ hybrids, and their F_2_ populations. Observations in 2012 and 2013 yielded similar results. The mean GWR values of the domesticated parental line and F_1_ hybrids from W43 × D26 and D26 × W43 were 1.035 ± 0.006, 1.130 ± 0.045, and 1.142 ± 0.035 in 2012 and 1.025 ± 0.005, 1.023 ± 0.012, and 1.025 ± 0.004 in 2013, respectively. There were no significant differences in mean GWR values among the domesticated parental line and either of the two F_1_ hybrids obtained from the reciprocal crossing. The mean GWR values of the wild parental line, 0.640 ± 0.014 in 2012 and 0.643 ± 0.023 in 2013, were significantly lower than the values obtained for the domesticated parental line and F_1_ hybrids in both 2012 and 2013 (Supplemental Tables 6, 7, Fig. 8A–8C).

There was no significant difference in GWR values between F_2_ populations derived from reciprocal cross combinations (*t* = 0.35, *p* = 0.73 in 2012, and *t* = 0.35, *p* = 0.72 in 2013). The GWR values in F_2_ populations showed a wide variation, ranging from 0.478 to 1.291 in 2012 (Supplemental Table 6) and from 0.520 to 1.182 in 2013 (Supplemental Table 7), and their frequency distributions showed distinct modes between 1.05 and 1.10 in 2012, and between 1.00 and 1.05 in 2013 (Fig. 8D). F_2_ populations had minor distributions with lower GWR values despite having major distributions with modes ranging from 1.00 to 1.10. The number of plants in both minor and major distributions with a limit value of 0.75 or 0.80 did not significantly deviate from the segregation ratio of 1:15 (Table 7). Here, the limit values were adopted based on the ranges of the wild (W43) and domesticated (D26) parental lines (Supplemental Tables 6, 7, Fig. 8A, 8B).

### Germination in F_1_ grains, F_1_ hybrids, and F_2_ populations

The GR and GI values in F_1_ grains obtained from the maternal plants after reciprocal crosses between a wild and a domesticated parental line were examined in 2012 (Supplemental Table 6). When the wild line W43 was used as the maternal parent, F_1_ grains of the first florets in two-grained spikelets had lower GR and GI values of 0.89 and 0.88, respectively, which were intermediate between those of the wild and domesticated parental lines, while F_1_ grains of the second florets had both the GR and GI values of 1.00. When the domesticated line D26 was used as the maternal parent, F_1_ grains of both the first and second florets in two-grained spikelets showed high GR and GI values of 1.00. These facts imply that the genotypes of both the maternal parents and the embryos of F_1_ grains may influence the degree of dormancy in F_1_ grains.

**Fig. 9.**
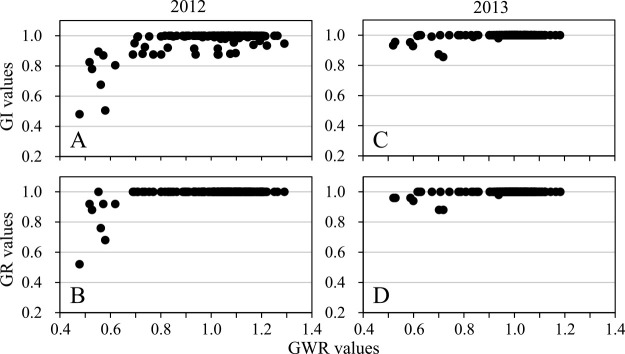
Scatter diagrams showing correlations of GI (A and C) and GR values (B and D) with GWR values in the F_2_ populations observed in 2012 (A and B) and 2013 (C and D). Data from reciprocal cross combinations are pooled because there was no significant difference between them.

Fig. 8E–8H depict the frequency distributions of the GI values of the first floret grains of two-grained spikelets in both wild and domesticated parental lines, their F_1_ hybrids, and their F_2_ populations observed in 2012 and 2013. The GI values of the wild parental line were higher in 2013 than in 2012 (Fig. 8E). The first floret grains of the F_1_ hybrids showed very high GI values, ranging from 0.99 to 1.00, which were comparable to those of the second floret grains and the domesticated parental line (Fig. 8E–8G, Supplemental Tables 6, 8). There was no significant difference in GI values between F_2_ populations derived from reciprocal cross combinations (*z* = 0.36, *p* = 0.72 in 2012, and *z* = 1.21, *p* = 0.23 in 2013). In the F_2_ populations, the GI of the first floret grains ranged from 0.48 to 1.00 among the 170 plants observed in 2012 and from 0.86 to 1.00 among the 162 plants observed in 2013. The latter was significantly higher than the former (*z* = 5.99, *p* < 0.01) (Fig. 8H, Supplemental Tables 6, 8). In both observation years, most F_2_ plants showed very high GI values: 153 (90.0%) of 170 plants in 2012 and 160 (98.8%) of 162 plants in 2013 had GI values higher than 0.90. The variation patterns of both the GI and GR values in the F_2_ populations were distinctly different between the high-GWR and low-GWR groups (Fig. 9), with both variables in the high-GWR group being significantly higher than those in the low-GWR group (Table 8).

### Selection of low-GWR lines having high- or low-GI values

**Fig. 10.**
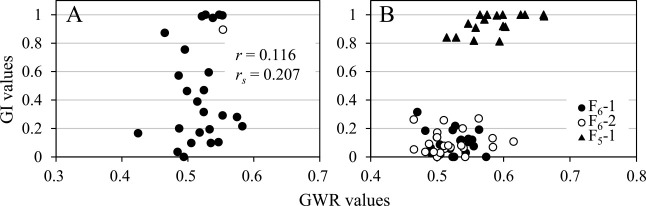
Scatter diagrams showing correlations between GWR and GI values. A: F_3_ population (solid circles) derived from self-pollination of the F_2_ plant 12403A-24 (open circle). Pearson’s and Spearman’s correlation coefficients are indicated with *r* and *r_s_*, respectively. B: Two F_6_ populations, F_6_-1 (solid circles) and F_6_-2 (open circles), derived from an F_5_ plant with a low-GI value, and the F_5_ population, F_5_-1 (solid triangles), derived from an F_4_ plant with a high-GI value.

Twenty-five F_3_ plants derived from self-pollination of the F_2_ plant, 12403A-24, whose GWR was 0.553 and GI of the first floret grains in two-grained spikelets was 0.90 (Supplemental Table 6), were examined for their GWR and GI of the first floret grains in two-grained spikelets (Fig. 10A, Supplemental Table 9). Their GWR values ranged from 0.425 to 0.583, with a mean value of 0.518 ± 0.007, and their GI values ranged from 0 to 1, with a median of 0.31. Pearson’s and Spearman’s rank correlation coefficients between their GWR and GI values were 0.116 and 0.207, respectively, and there was no significant correlation between the two variables.

Four F_4_ populations were established from four F_3_ plants with varying GI values: population F_4_-1 was derived from the F_3_ plant, 15502-14 (GWR = 0.494, GI = 0), F_4_-2 from 15502-22 (GWR = 0.505, GI = 0.10), F_4_-3 from 15502-17 (GWR = 0.499, GI = 0.46), and F_4_-4 from 15502-13 (GWR = 0.527, GI = 1) (Supplemental Table 9). The mean GI values of the four F_4_ populations were 0.41, 0.56, 0.85, and 0.94, respectively (Table 9). Population F_4_-1 was divided into two subpopulations, F_4_-1a and F_4_-1b, according to the GI values (Table 9, Supplemental Fig. 3A): the former ranged from 0.07 to 0.24 (median = 0.19) while the latter ranged from 0.45 to 0.73 (median = 0.65) (Table 9, Supplemental Table 10). GI median values of the other three populations, F_4_-2, F_4_-3, and F_4_-4, were 0.54, 0.85, and 0.96, respectively (Table 9, Supplemental Fig. 3B–3D). Except for the comparisons between F_4_-1a and F_4_-1b and between F_4_-1b and F_4_-2, the GI values of these five populations, including the two subpopulations, were significantly different from each other, whereas their GWR values were not significantly different from each other, except for the comparison between F_4_-3 and F_4_-4 (Table 9).

GWR and GI values of two F_6_ populations, F_6_-1 and F_6_-2, derived from a plant in the population F_4_-1a (F_4_-1a-6) with a low GI of 0.07 and an F_5_ population, F_5_-1, derived from a plant in the population F_4_-4 (F_4_-4-21) with a high GI of 0.98 (Supplemental Table 10) were analyzed (Table 10, Supplemental Table 11). Populations F_6_-1 and F_6_-2 had low GWR values with means of 0.524 and 0.521, respectively, and low GI values with means of 0.10 and medians of 0.10 and 0.08, respectively (Supplemental Fig. 3E, 3F). There was no significant difference in GWR or GI values between the two populations. Population F_5_-1, with a mean GWR of 0.587 and mean and median GI values of 0.94 and 0.97, respectively (Supplemental Fig. 3G), had significantly higher GWR and GI values than the two F_6_ populations. GI values were distinctly different between the population F_5_-1 and the two F_6_ populations without overlapping, although the GWR values of more than half of the plants in the three populations were distributed in the same range, between 0.50 and 0.60 (Fig. 10B).

## Discussion

### Variation of intraspikelet differences in grain weight and seed dormancy

This study revealed that the mean one-grain weight of the first floret grains in two-grained spikelets was significantly lower than that of the second floret grains in wild accessions but significantly higher than that of the second floret grains in domesticated accessions. The mean GWR values of wild accessions were significantly lower than those of domesticated accessions (Table 1). These results coincide with those in previous studies (Cook 1913, Harlan 1992, Nave *et al.* 2016). Additionally, the current study indicated that the first floret grains of one-grained spikelets in wild accessions were large and their weights were similar to those of the second floret grains in two-grained spikelets, strongly implying that the first floret grains became smaller than the second floret grains not only because of their positions in spikelets but also due to the development of the second floret grains in the same spikelets. Only first floret grains in two-grained spikelets showed varying degrees of dormancy with GI values ranging from 0 to 1, which positively correlated with their GWR values, according to the current study (Table 6, Figs. 4, 6, Supplemental Table 4). The viability of nongerminated grains of low-GI accessions was not chemically tested, but most of them were deemed viable, judging from the fact that their GR and GI levels had significantly increased two months later (Table 5, Supplemental Table 4). Conversely, the grains of the two other grain groups in wild accessions were nondormant and germinated shortly after being sown, similar to those of domesticated accessions. This result indicates that the GR or GI data from only the first floret grains in two-grained spikelets can provide a more accurate estimate of the degree of dormancy for each individual than data from the total grains of three grain groups.

Furthermore, the current study indicated a wide range of GWR and GI values in wild emmer accessions. Thus, one-grain weights of the first and second florets were not significantly different in more than a quarter of the 60 wild accessions examined, and their GWR was within the range of domesticated accessions (Fig. 2, Supplemental Table 2), with some of them showing high GR and GI values, similar to domesticated accessions (Fig. 6). These results indicate that wild emmer populations consist of individuals with varying degrees of seed dormancy, including those that are nondormant and have large first floret grains. In this study, there were wide variations in GWR and GI values within each of the eight geographical regions, but there were no evident differences in these variables among the geographical regions (Supplemental Table 5). Volis (2016) estimated the contribution of intraspikelet seed dormancy difference along an aridity gradient in four Israeli wild emmer wheat populations and discovered that the first floret grains from the most arid location had the lowest germination fraction in the first year under controlled irrigation condition, whereas those from the least arid location had the highest. Thus, seed dormancy difference is regarded as a bet-hedging trait, allowing a population to survive under insufficient and unpredictable rainfall (Philippi 1993, Volis 2016), and among wild emmer wheat populations, a variation in the frequency of both dormant and nondormant individuals may exist under different water availability conditions.

### Genetic control of intraspikelet differences in grain weight and seed dormancy

In this study, the GWR of both wild and domesticated accessions, as well as the GI of the first floret grains in two-grained spikelets of wild accessions, were considerably different but significantly correlated across harvest years and growing environments (Tables 3, 5). These results indicate that the ranks in GWR and GI values among wild emmer accessions are significantly maintained under different environmental conditions, confirming that the intraspikelet differences in grain weight and seed dormancy are genetically controlled traits influenced by environmental factors. Nave *et al.* (2016) revealed that QTLs associated with intraspikelet differences in grain size and seed dormancy are co-located on chromosome 4B of emmer wheat, and termed it *QGD-4BL*. Furthermore, it was proposed that the intraspikelet differences in grain size and dormancy could be controlled by a single gene with pleiotropic effects or a set of tightly linked genes. The current findings from a survey of F_1_ hybrids and segregations in F_2_ populations strongly suggest that the intraspikelet difference in grain weight, represented by GWR, is controlled by two independently inherited major loci and that a high-GWR spikelet with uniform grains in its two florets is a dominant trait (Table 7, Fig. 8A–8D). The two loci might be located on homoeologous chromosomes in A and B genomes. Ohta (2009) reported that wild einkorn wheat (*T. monococcum* ssp. *aegilopoides*, genome AA) also showed intraspikelet differences in grain weight and seed dormancy. Further studies on the diploid A-genome donor of emmer wheat, *T. urartu*, and its close relative *T. monococcum* are necessary for more precise insight into the genetic control of intraspikelet grain weight and dormancy differences. A discrepancy in the number of loci controlling intraspikelet grain size (weight) difference between the results from Nave *et al.* (2016) and those from the current study could be due to a difference in genotypes of parental lines used, as genetic analyses performed by crossing experiments revealed the inheritance of alleles that differed between the parental lines.

Furthermore, the current finding that low-GWR populations with either a low or high GI value could be successfully selected in F_5_ and F_6_ populations implies that major loci control seed dormancy independently of intraspikelet grain weight difference. These dormancy loci could be crucial and common in wild species from the tribe Hordeae, and they might be homoeologous to those located on chromosome 5H in wild barley (*Hordeum vugare* ssp. *spontaneum*), whose seed dispersal units contain only a single grain (Han *et al.* 1996, Hori *et al.* 2007, Sato *et al.* 2016). The current findings indicate that the degree of dormancy in emmer wheat is a combined trait of GWR and dormancy genotypes: the dormant phenotype appears only under low-GWR phenotypes, whereas the first floret grains under high-GWR phenotypes show nondormant phenotypes regardless of the allele type of the dormancy loci. In this study, F_1_ grains obtained from a cross with the wild line (W43) as a maternal parent exhibited low-GWR and low-GI phenotypes, whereas those with the domesticated line (D26) as a maternal parent exhibited high-GWR and high-GI phenotypes, though their embryos had the same genotypes and were heterozygous for alleles derived from the two parental lines. Hence, it is inferred that in the former F_1_ grains, the dormant phenotype determined by the embryo genotype appeared under the low-GWR phenotype determined by the wild maternal parent’s genotype. However, the latter F_1_ grains exhibited nondormant phenotypes under the high-GWR phenotype determined by the genotype of the domesticated maternal line. Furthermore, it is indicated that a certain portion of F_2_ plants obtained in this study can have dormant genotypes but showed nondormant phenotypes due to large grain size. Further crossing experiments that exclude the effects of intraspikelet differences in grain size are necessary for more precise insight into the genetic control of seed dormancy in wild emmer wheat. Low-GWR lines with either a low or high GI value selected from the current F_5_ or F_6_ populations will be suitable for such crossing experiments.

### Establishment of nondormant populations through the domestication process

Nave *et al.* (2016) tested 51 domesticated emmer wheat accessions (*T. turgidum* ssp. *dicoccum*) for intraspikelet grain dimension and dormancy differences and discovered no significant differences between the first and second grains. It was suggested that the *QGD-4BL* locus has been fixed during the transition from wild subspecies (ssp. *dicoccoides*) to the domesticated hulled emmer wheat (ssp. *dicoccum*). Furthermore, all 82 domesticated emmer accessions surveyed in this study exhibited high-GWR and high-GI values.

In the early aceramic Neolithic site of Çayönü Tepesi, dating from 9200 to 8700 BP, only slender *dicoccoides*-type grains and no typical *dicoccum*-type plump grains were discovered in the lowermost layers yielding emmer grains, whereas *dicoccoides*-type grains were absent and only *dicoccum*-type grains were discovered in the upper half layers belonging to the aceramic occupation (van Zeist and Roller 1991/1992). Willcox (2004) compared the grain size data of emmer wheat published by van Zeist and Roller (1991/1992) and discovered that the grain size in later layers was larger than that of early layers of the sites and the grain size in later layers corresponded well with domesticated grains present in the Chalcolithic site Tell Kosak Shamali (6050 ± 100 BP). These facts support the hypothesis by Nave *et al.* (2016) that spikelets with uniform grains had been fixed in populations during the early domestication stage. Willcox (2004) postulated that this early and rapid increase in grain size resulted from the introduction of naturally occurring ecotypes with larger grains from elsewhere. The selection of natural populations rich in plants with large and uniform grain size might have simultaneously promoted the selection of populations rich in plants with nondormant phenotypes, as large and uniform grains in two-grained spikelets can quickly germinate at the start of autumn rains, regardless of their dormancy genotypes, as shown in this study (Table 8, Fig. 9). There might have been populations rich in nondormant plants with large grains grown in more predictable water-rich environments, as indicated by Volis (2016). However, the selection of nondormant phenotypes has not always been accompanied by the selection for nondormant genotypes because the degree of dormancy is a combined trait of GWR and dormancy genotypes. It is unclear how the selection of genetically nondormant populations occurred (or did not occur) during the domestication process. Assessments of the variation in frequencies of individuals with nondormant phenotypes and genotypes among wild emmer wheat populations will shed light on their adaptation strategy to natural habitats and early domestication environments.

### Potential of seed dormancy alleles of wild emmer wheat in PHS tolerance breeding programs

PHS is a long-standing challenge for wheat production worldwide (Nakamura 2018). To develop PHS-tolerant varieties, several major dormancy genes and QTLs have been located, such as *MFT* (*QPhs-3A.1*) on chromosome 3A, *MKK3* (*Phs1*) on chromosome 4A, and *QPhs-5AL* on chromosome 5A, in common wheat (Himi *et al.* 2011, Kottearachchi *et al.* 2006, Mori *et al.* 2005, Torada *et al.* 2005, 2008; also see Nakamura 2018 for review). However, few PHS-tolerant genetic resources have been developed in durum wheat (Kato *et al.* 2017). Here, dormancy loci independent of GWR did not affect dormancy phenotypes under high-GWR phenotypes, whereas prominent effects were observed under low-GWR phenotypes when grains were sown in late August to early September, just before the autumn rainy season in Southwest Asia. The effects of these dormancy loci on the germination behavior prior to after-ripening under high-GWR phenotypes corresponding to domesticated accessions remain unclear. Germination tests should be performed immediately after harvesting to estimate their degree of phenotypic dormancy. It could be useful for identifying new potential sources of PHS-tolerant genes, especially for improving durum wheat.

## Author Contribution Statement

SO designed the research, conducted the experiments, analyzed the data and wrote the manuscript.

## Supplementary Material

Supplemental Figures

Supplemental Tables

## Figures and Tables

**Table 1.** The mean one-grain weight of the first and second floret grains and GWR values in two-grained spikelets, and the results of a paired *t*-test between the mean one-grain weight of the first and second florets (*t_1st-2nd_*) and a *t*-test between the mean GWR of wild and domesticated emmer wheat (*t_wild-dom_*)

Year of observation	Plant groups	No. of accessions	One-grain weight (mg)					GWR	
			First florets		Second florets	*t_1st-2nd_*		Mean ± SE	*t_wild-dom_*
			Mean ± SE		Mean ± SE				
2009	Wild	60	22.79 ± 0.83		29.09 ± 1.18	10.27**		0.804 ± 0.017	10.82**
	Domesticated	37	40.90 ± 1.86		38.96 ± 2.03	5.22**		1.072 ± 0.015	
2010	Wild	66	18.99 ± 0.50		26.98 ± 0.78	16.22**		0.713 ± 0.013	20.44**
	Domesticated	69	37.60 ± 0.91		34.84 ± 0.89	6.59**		1.087 ± 0.013	
2014 Field	Wild	57	22.82 ± 0.60		34.65 ± 1.03	22.68**		0.666 ± 0.008	30.30**
	Domesticated	35	46.06 ± 1.25		46.11 ± 1.31	0.22		1.001 ± 0.005	
2014 Greenhouse	Wild	53	18.01 ± 0.64		27.88 ± 1.04	18.70**		0.652 ± 0.010	25.75**
	Domesticated	35	41.44 ± 1.41		39.68 ± 1.47	5.01**		1.050 ± 0.010	

** indicates the mean values significantly differ at *p* = 0.01.

**Table 2.** The mean one-grain weight (mg) of the three grain groups, as well as the results of repeated measures analysis of variance (Holm method) among their means in wild and domesticated emmer wheat

Year of observation	Plant groups	No. of accessions	Two-grained spikelets*^a^*				One-grained spikelets*^a^*
			First florets		Second florets		
			Mean ± SE		Mean ± SE		Mean ± SE
2009	Wild	31	22.64 ± 1.16 ^a^		27.65 ± 1.56 ^b^		27.08 ± 1.42 ^b^
	Domesticated	29	39.42 ± 2.16 ^a^		37.27 ± 2.38 ^b^		32.86 ± 2.05 ^c^
2010	Wild	65	18.94 ± 0.50 ^a^		26.85 ± 0.78 ^b^		22.92 ± 0.61 ^c^
	Domesticated	69	37.60 ± 0.91 ^a^		34.84 ± 0.89 ^b^		33.04 ± 0.78 ^c^
2014 Field	Wild	34	22.28 ± 0.90 ^a^		33.76 ± 1.49 ^b^		28.88 ± 1.12 ^c^
	Domesticated	22	46.47 ± 1.96 ^a^		46.22 ± 2.04 ^a^		43.07 ± 2.11 ^b^
2014 Greenhouse	Wild	44	17.92 ± 0.72 ^a^		27.59 ± 1.15 ^b^		23.45 ± 1.30 ^c^
	Domesticated	30	41.11 ± 1.62 ^a^		38.95 ± 1.66 ^b^		34.97 ± 1.64 ^c^

*^a^* Mean weights with different superscrips significantly differ at *p* = 0.01 within each plant group.

**Table 3.** The results of paired *t*-test (*t*) and Pearson’s correlation coefficients (*r*) for the mean one-grain weight of the first and second floret grains, as well as the GWR in two-grained spikelets, between different harvest years (2009 vs. 2010) and growing environments (field vs. greenhouse) in 2014

Comparisons	No. of accessions	Mean ± SE		Mean ± SE		*t*	*r*
Plant groups							
Variables							
Florets							
Between harvest years		2009		2010		between 2009/2010	
Wild emmer wheat							
One-grain weight (mg)							
First florets	59	22.91 ± 0.84		18.74 ± 0.53		8.00**	0.801**
Second florets	59	29.16 ± 1.20		26.65 ± 0.81		3.88**	0.862**
GWR	59	0.807 ± 0.017		0.712 ± 0.014		5.35**	0.348**
Domesticated emmer wheat							
One-grain weight (mg)							
First florets	24	43.83 ± 2.20		35.58 ± 1.83		4.47**	0.594**
Second florets	24	42.28 ± 2.35		33.10 ± 1.93		4.46**	0.553**
GWR	24	1.053 ± 0.017		1.093 ± 0.030		1.17	0.007
Between growing environments in 2014		Field (F)		Greenhouse (GH)		between F/GH	
Wild emmer wheat							
One-grain weight (mg)							
First florets	55	22.77 ± 0.61		18.44 ± 0.71		6.02**	0.418**
Second florets	55	34.64 ± 1.06		28.25 ± 1.03		6.52**	0.558**
GWR	55	0.665 ± 0.008		0.656 ± 0.011		0.86	0.390**
Domesticated emmer wheat							
One-grain weight (mg)							
First florets	35	46.06 ± 1.25		40.88 ± 1.53		5.85**	0.816**
Second florets	35	46.11 ± 1.31		39.05 ± 1.61		7.88**	0.830**
GWR	35	1.001 ± 0.005		1.055 ± 0.011		6.00**	0.538**

* and ** indicate significant at *p* = 0.05, and at *p* = 0.01, respectively.

**Table 4.** Means and medians of GI values for the three grain groups, nonparametric Friedman test results among the three grain groups in each plant group, and Mann–Whitney *U* test results (*z*) between wild and domesticated taxa

Year of observation	Plant groups	No. of accessions	Two-grained spikelets								One-grained spikelets		
			First florets				Second florets				First florets		
			Mean	Median*^a^*	*z*		Mean	Median*^a^*	*z*		Mean	Median*^a^*	*z*
2009 September	Wild	53	0.32	0.19 ^a^	7.95**		0.98	1.00 ^b^	3.08**		0.91	0.98 ^b^	5.56**
	Domesticated	34	1.00	1.00 ^a^			1.00	1.00 ^a^			1.00	1.00 ^a^	
2009 November	Wild	15	0.65	0.88 ^a^	4.78**		1.00	1.00 ^b^	–		0.99	1.00 ^b^	1.77
	Domesticated	24	1.00	1.00 ^a^			1.00	1.00 ^a^			1.00	1.00 ^a^	
2010	Wild	63	0.49	0.52 ^a^	10.43**		1.00	1.00 ^b^	2.77**		0.95	0.98 ^c^	7.48**
	Domesticated	69	1.00	1.00 ^a^			1.00	1.00 ^a^			1.00	1.00 ^a^	
2014 Field	Wild	35	0.22	0.17 ^a^	6.53**		1.00	1.00 ^b^	0.95		0.93	1.00 ^b^	3.14**
	Domesticated	23	1.00	1.00 ^a^			1.00	1.00 ^a^			0.99	1.00 ^a^	
2014 Greenhouse	Wild	45	0.26	0.22 ^a^	7.63**		0.99	1.00 ^b^	2.28*		0.86	0.91 ^c^	5.97**
	Domesticated	31	1.00	1.00 ^a^			1.00	1.00 ^a^			1.00	1.00 ^a^	

*^a^* Different superscripts indicate GI values significantly differ at *p* = 0.01 within each plant group; * and ** indicate significantly different at *p* = 0.05, and at *p* = 0.01, between wild and domesticated taxa, respectively.

**Table 5.** The results from Wilcoxon signed-rank test (*z*) and Spearman’s rank correlation coefficients (*r_s_*) for GI values of the three grain groups between different sowing months (September vs. November) in 2009, harvest years (2009 vs. 2010), and growing environments (field vs. greenhouse) in 2014

Comparisons	Florets	No. of accessions	GI						*z*	*r_s_*
Plant groups			Mean	Median		Mean	Median			
Spikelets										
Between sowing months in 2009			September			November			between Sept./Nov.	
Wild emmer wheat										
Two-grained	First	53	0.28	0.18		0.58	0.66		5.873**	0.845**
	Second	53	0.98	1.00		1.00	1.00		3.920**	–
One-grained	First	16	0.94	0.98		0.99	1.00		2.293*	0.212
Domesticated emmer wheat										
Two-grained	First	37	1.00	1.00		1.00	1.00		1.000	–
	Second	37	1.00	1.00		1.00	1.00		1.342	–
One-grained	First	23	1.00	1.00		1.00	1.00		1.000	–
Between harvest years			2009 (Sept.)			2010			between 2009/2010	
Wild emmer wheat										
Two-grained	First	58	0.30	0.19		0.50	0.54		4.171**	0.447**
	Second	59	0.98	1.00		1.00	1.00		3.049**	0.050
One-grained	First	52	0.91	0.97		0.96	0.98		2.293*	0.173
Domesticated emmer wheat										
Two-grained	First	24	1.00	1.00		1.00	1.00		–	–
	Second	24	1.00	1.00		1.00	1.00		0.535	–0.063
One-grained	First	23	1.00	1.00		1.00	1.00		1.000	–
Between growing environments in 2014			Field(F)			Greenhouse(GH)			between F/GH	
Wild emmer wheat										
Two-grained	First	54	0.20	0.16		0.26	0.23		3.041**	0.459**
	Second	54	1.00	1.00		1.00	1.00		0.549	–0.123
One-grained	First	31	0.92	0.99		0.88	0.94		1.305	0.302
Domesticated emmer wheat										
Two-grained	First	35	1.00	1.00		1.00	1.00		2.023*	–
	Second	35	1.00	1.00		1.00	1.00		1.826	–
One-grained	First	22	0.99	1.00		1.00	1.00		1.342	–

* and ** indicate significant at *p* = 0.05, and at *p* = 0.01, respectively.

**Table 6.** Spearman’s rank correlation coefficients (*r_s_*) between GI values and one-grain weights of the first floret grains in two-grained spikelets and between GI and GWR values in wild emmer wheat

Observation	No. of accessions	*r_s_* between GI and	
		One-grain weight	GWR
2009 September	60	0.111	0.628**
2009 November	53	0.100	0.725**
2010	65	0.228	0.322**
2014 Field	57	–0.354**	0.627**
2014 Greenhouse	53	–0.345*	0.453**

* and ** indicate that correlations are significant at *p* = 0.05, and at *p* = 0.01, respectively.

**Table 7.** Segregation of GWR values in F_2_ populations observed in 2012 and 2013, and the results of χ^2^ tests. Data from reciprocal crosses were pooled because there was no significant difference between them

Year of observation	No. of F_2_ plants			χ^2^ (1:15)	*p*	No. of F_2_ plants		χ^2^ (1:15)	*p*
	Total	GWR				GWR			
		<0.75	≥0.75			<0.80	≥0.80		
2012	170	14	156	1.144	0.285	16	154	2.900	0.089
2013	162	12	150	0.370	0.543	14	148	1.582	0.208

**Table 8.** Comparison in GI and GR values between low- and high-GWR groups with the limit GWR value at 0.75 or 0.80 in the F_2_ populations observed in 2012 and 2013, and the result of Mann–Whitney *U* test (*z*)

Year of observation	Variables	Low-GWR group*^a^*						High-GWR group*^a^*					*z*
		No. of plants	Min.	Max.	Median	IQR		No. of plants	Min.	Max.	Median	IQR	
Limit GWR value = 0.75													
2012	GI	14	0.48	0.995	0.87	0.13		156	0.88	1.00	1.00	0.005	6.786**
	GR		0.52	1.00	0.96	0.11			1.00	1.00	1.00	0.00	8.990**
2013	GI	12	0.86	1.00	0.97	0.07		150	0.98	1.00	1.00	0.00	9.076**
	GR		0.88	1.00	0.98	0.05			0.98	1.00	1.00	0.00	8.115**
Limit GWR value = 0.80													
2012	GI	16	0.48	0.995	0.88	0.13		154	0.88	1.00	1.00	0.003	7.114**
	GR		0.52	1.00	1.00	0.09			1.00	1.00	1.00	0.00	8.356**
2013	GI	14	0.86	1.00	0.99	0.06		148	0.98	1.00	1.00	0.00	8.317**
	GR		0.88	1.00	1.00	0.04			0.98	1.00	1.00	0.00	7.445**

*^a^* IQR indicates the interquartile range; ** indicates that GI and GR values significantly differ at *p* = 0.01 between the low- and high-GWR groups.

**Table 9.** Mean GWR and mean and median GI values of F_4_ populations, and the results from Tukey–Kramer test for mean GWR (in the upper triangle) and Steel–Dwass test for GI (in the lower triangle) among the five F_4_ populations including two subpopulations

Population No.	No. of plants	GWR		GI*^a^*				Population No.				
		Mean ± SE		Mean	Median	IQR		F_4_-1a	F_4_-1b	F_4_-2	F_4_-3	F_4_-4
F_4_-1	9	0.550 ± 0.014		0.41	0.55	0.46						
F_4_-1a	4	0.532 ± 0.016		0.17	0.19	0.06			0.934	0.168	0.059	1.450
F_4_-1b	5	0.564 ± 0.020		0.59	0.65	0.18		2.449		0.962	1.256	0.318
F_4_-2	10	0.537 ± 0.028		0.56	0.54	0.09		2.828*	0.490		0.195	1.816
F_4_-3	40	0.534 ± 0.007		0.85	0.85	0.20		3.268**	3.252**	4.610**		2.914*
F_4_-4	24	0.572 ± 0.009		0.94	0.96	0.07		3.163*	3.476**	4.545**	3.213**	

*^a^* IQR indicates the interquartile range; * and ** indicate significantly different at *p* = 0.05, and at *p* = 0.01, respectively.

**Table 10.** Mean GWR and mean and median GI values of an F_5_ and two F_6_ populations selected from high- or low-GI populations, and the results of the Tukey–Kramer test for mean GWR (in the upper triangle) and Steel–Dwass test for GI (in the lower triangle) among the three populations

Population No.	No. of plants	GWR		GI*^a^*				Population No.		
		Mean ± SE		Mean	Median	IQR		F_6_-1	F_6_-2	F_5_-1
F_6_-1	22	0.524 ± 0.006		0.10	0.10	0.10			0.234	5.417**
F_6_-2	22	0.521 ± 0.008		0.10	0.08	0.08		0.000		5.636**
F_5_-1	17	0.587 ± 0.010		0.94	0.97	0.09		5.308**	5.307**	

*^a^* IQR indicates the interquartile range; ** indicates GWR and GI significantly differ at *p* = 0.01 between populations.
