# Supplementary material for: Genetic variation and genetic control of intraspikelet differences in grain weight and seed dormancy in wild and domesticated emmer wheats
Source: Breed Sci. 2022 Jun 29;72(3):198–212. doi: 10.1270/jsbbs.21060 (PMC9653192; doi:10.1270/jsbbs.21060)
Supplement: Supplementary file 1 — Supplemental Figures [file 72_198_s1.pdf]

doi: 10.1270/jsbbs.21060

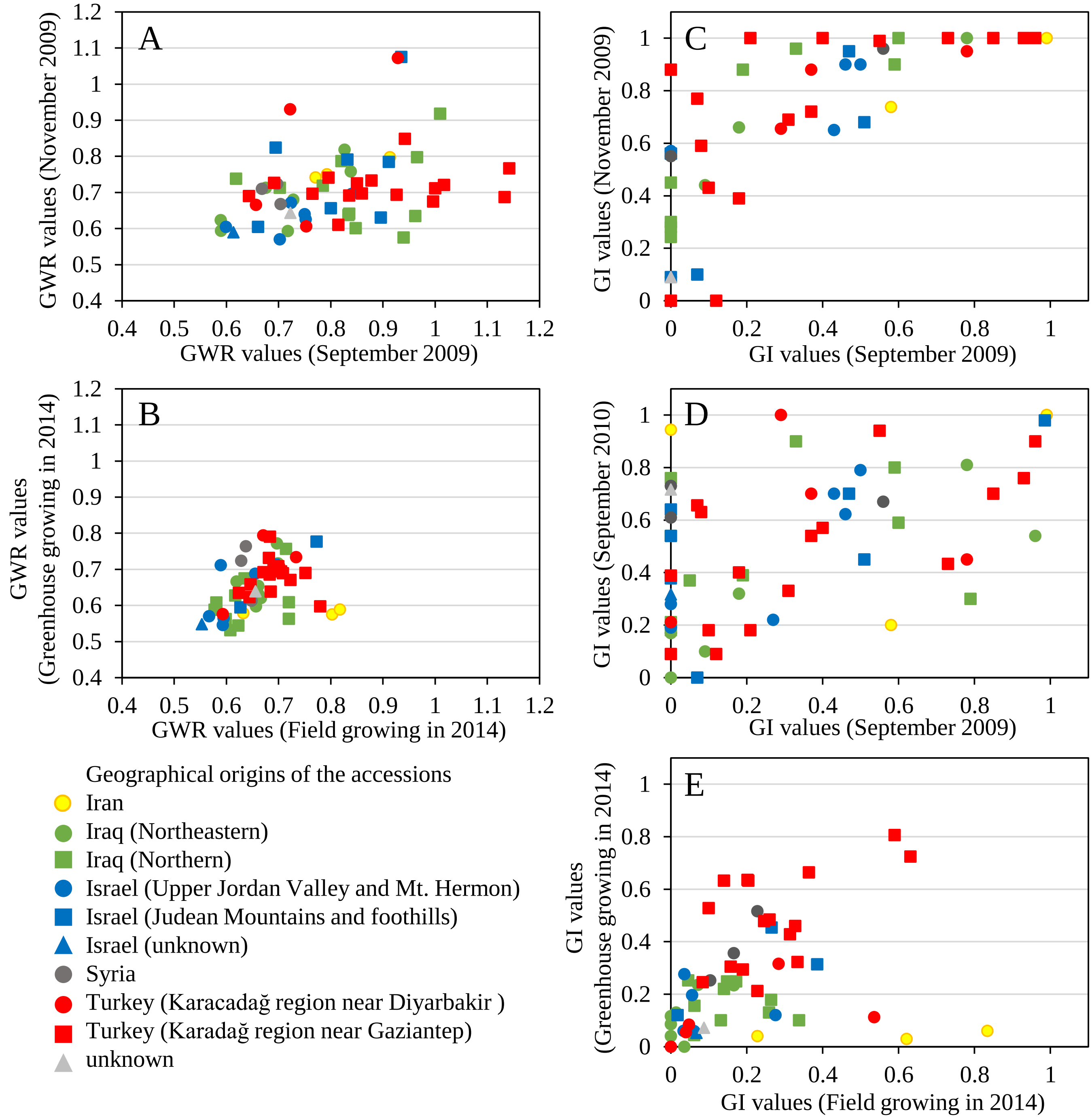

Supplemental Fig. 1. Scatter diagrams showing correlations in GWR values (A and B) and GI values of the first floret grains in two-grained spikelets (C–E) in wild emmer wheat accessions between sowing months in 2009 (C), harvest years (A and D), and growing environments in 2004 (B and E). For correlation coefficients and statistical significance, see Table 3 for GWR values and Table 5 for GI values.

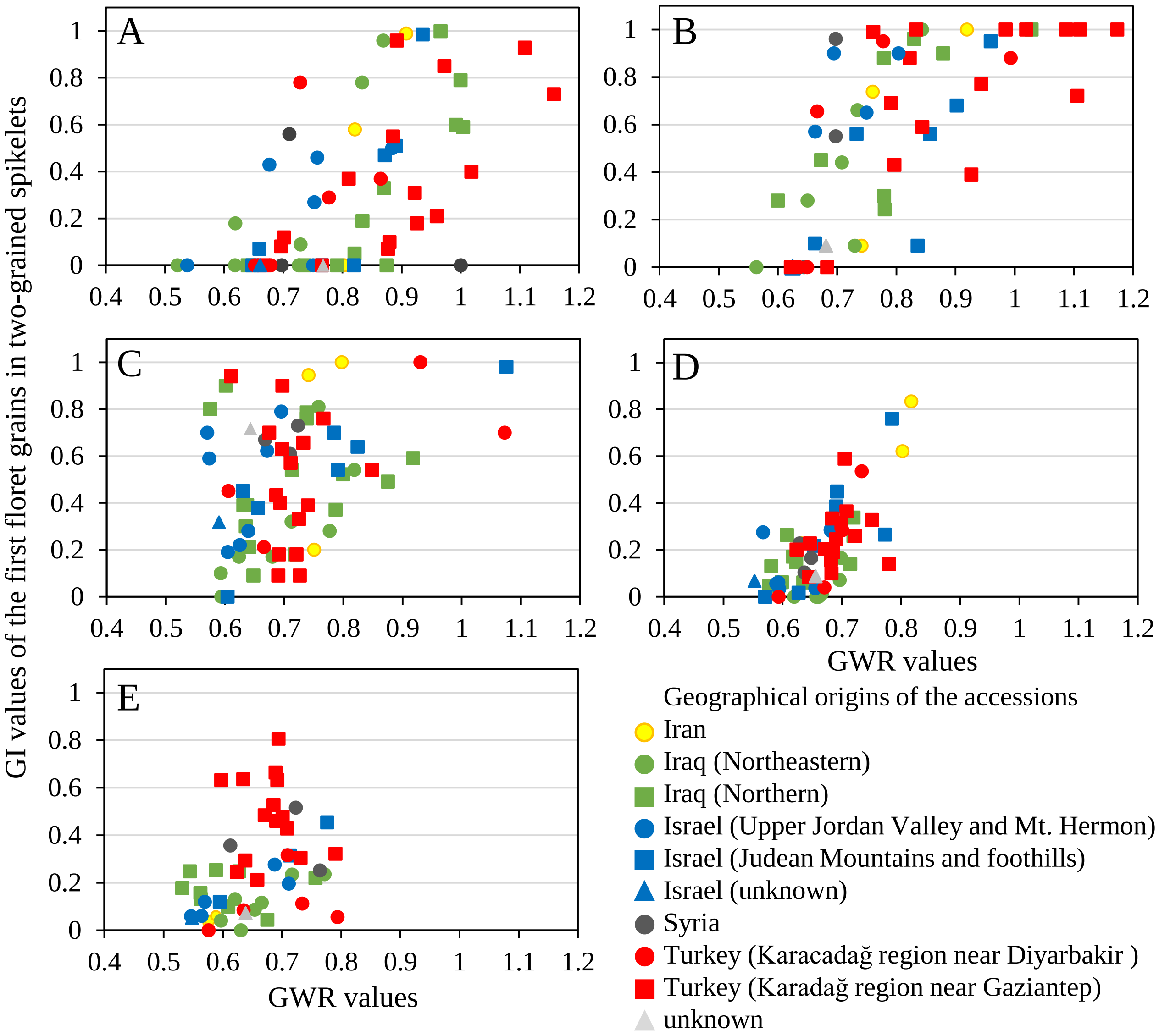

Supplemental Fig. 2. Scatter diagrams showing correlations between GWR and GI values in wild emmer wheat accessions. A: sown in September 2009, B: sown in November 2009, C: sown in September 2010, D: grown in an experimental field and sown in September 2014, and E: grown in a greenhouse and sown in September 2014. For correlation coefficients and statistical significance, see Table 6.

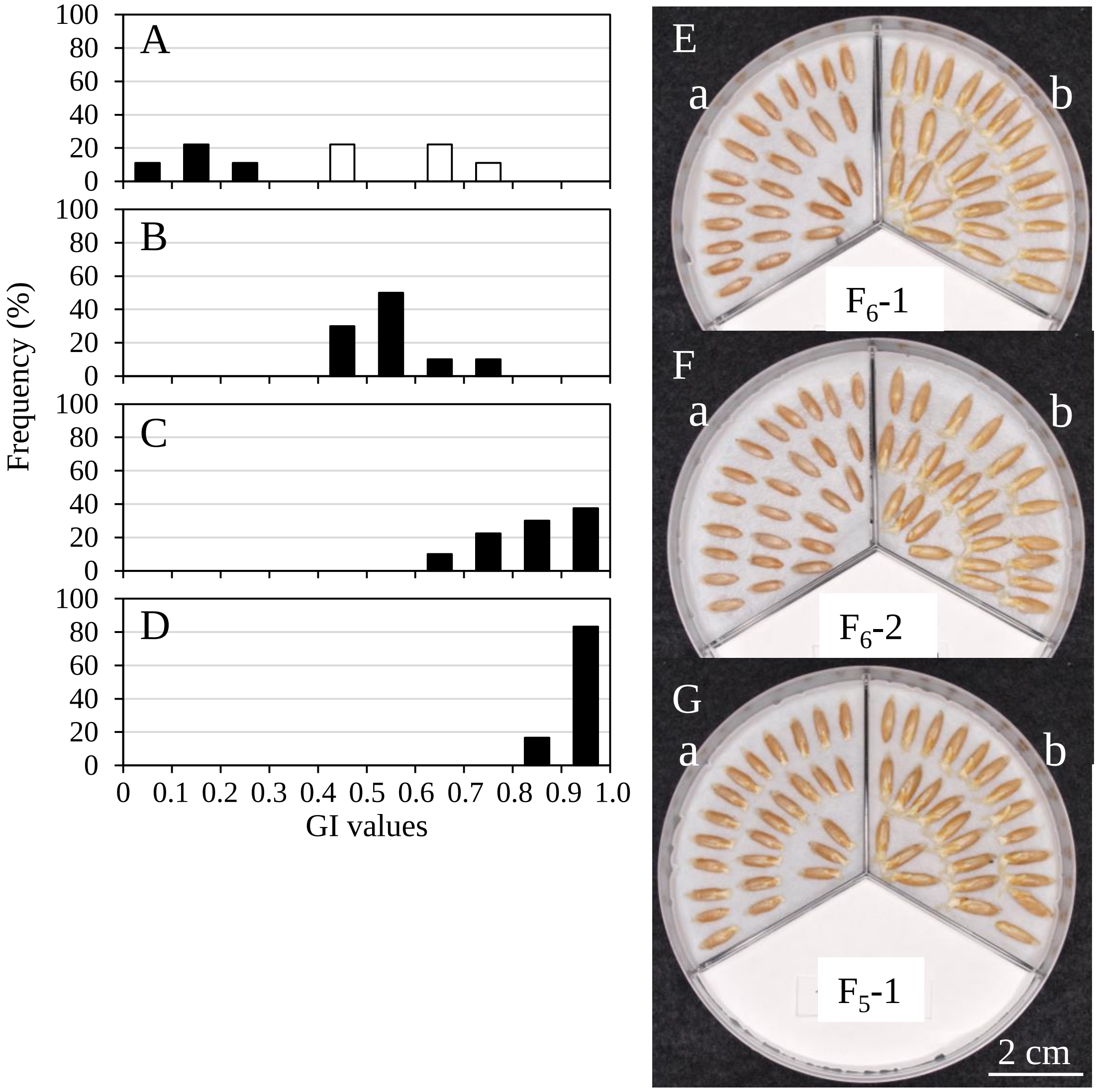

Supplemental Fig. 3. Frequency distributions of GI values of the first floret grains in two-grained spikelets in F_4 populations (A–D), and germination a day after sowing under 20ºC and continuous dark condition in F_5 and F_6 populations (E–G).

A: population F_4-1 whose individuals having GI values lower than 0.3 and higher than 0.4 were grouped into populations F_4-1a (solid columns) and F_4-1b (open columns), respectively, B: population F_4-2, C: population F_4-3, and D: population F_4-4.

E: population F_6-1 (Plant No. 8), F: population F_6-2 (Plant No. 14), and G: population F_5-1 (Plant No. 17). In each Petri dish, the first and second floret grains of two-grained spikelets are indicated with a and b, respectively. For the GRW, GR, and GI values of each plant, see Supplemental Tables 10 and 11.
