# Supplementary material for: Genetic variation and genetic control of intraspikelet differences in grain weight and seed dormancy in wild and domesticated emmer wheats
Source: Breed Sci. 2022 Jun 29;72(3):198–212. doi: 10.1270/jsbbs.21060 (PMC9653192; doi:10.1270/jsbbs.21060)
Supplement: Supplementary file 2 — Supplemental Tables [file 72_198_s2.pdf]

doi: 10.1270/jsbbs.21060

Supplemental Table 1. A list of the origins of 67 wild and 82 domesticated emmer wheat accessions used in this study

Accession No. in this study	Accession No. at origin [a]	Collection localities	Countries (Regions)
Wild emmer wheat (*Triticum turgidum* ssp. *dicoccoides*)			
W01	KU1921	155 km W of Mardin (Urfa-Mardin)	Turkey (Karacadağ region near Diyarbakir)
W02	KU1945	45 km SE of Maras (Maras-Gaziantep)	Turkey (Karadağ region near Gaziantep)
W03	KU1947	45 km SE of Maras (Maras-Gaziantep)	Turkey (Karadağ region near Gaziantep)
W04	KU1948	45 km SE of Maras (Maras-Gaziantep)	Turkey (Karadağ region near Gaziantep)
W05	KU1949	45 km SE of Maras (Maras-Gaziantep)	Turkey (Karadağ region near Gaziantep)
W06	KU1951	45 km SE of Maras (Maras-Gaziantep)	Turkey (Karadağ region near Gaziantep)
W07	KU1952	45 km SE of Maras (Maras-Gaziantep)	Turkey (Karadağ region near Gaziantep)
W08	KU1953	45 km SE of Maras (Maras-Gaziantep)	Turkey (Karadağ region near Gaziantep)
W09	KU1955	45 km SE of Maras (Maras-Gaziantep)	Turkey (Karadağ region near Gaziantep)
W10	KU1957	45 km SE of Maras (Maras-Gaziantep)	Turkey (Karadağ region near Gaziantep)
W11	KU1959A	45 km SE of Maras (Maras-Gaziantep)	Turkey (Karadağ region near Gaziantep)
W12	KU1959B	45 km SE of Maras (Maras-Gaziantep)	Turkey (Karadağ region near Gaziantep)
W13	KU1972B	45 km SE of Maras (Maras-Gaziantep)	Turkey (Karadağ region near Gaziantep)
W14	KU1974	45 km SE of Maras (Maras-Gaziantep)	Turkey (Karadağ region near Gaziantep)
W15	KU1976B	45 km SE of Maras (Maras-Gaziantep)	Turkey (Karadağ region near Gaziantep)
W16	KU1978B	45 km SE of Maras (Maras-Gaziantep)	Turkey (Karadağ region near Gaziantep)
W17	KU1991	45 km SE of Maras (Maras-Gaziantep)	Turkey (Karadağ region near Gaziantep)
W18	KU8536	20.3 km S from Sulaymaniyah to Qara Dagh	Iraq (Northeastern)
W19	KU8537	20.3 km S from Sulaymaniyah to Qara Dagh	Iraq (Northeastern)
W20	KU8538	20.3 km S from Sulaymaniyah to Qara Dagh	Iraq (Northeastern)
W21	KU8539	20.3 km S from Sulaymaniyah to Qara Dagh	Iraq (Northeastern)
W22	KU8541	20.3 km S from Sulaymaniyah to Qara Dagh	Iraq (Northeastern)
W23	KU8736A	SSW of Rowanduz	Iraq (Northeastern)
W24	KU8736B	SSW of Rowanduz	Iraq (Northeastern)
W25	KU8737	SSW of Rowanduz	Iraq (Northeastern)
W26	KU8804	North slope of Jabal Sinjar, N of Kursi	Iraq (Northern)
W27	KU8805	North slope of Jabal Sinjar, N of Kursi	Iraq (Northern)
W28	KU8806	North slope of Jabal Sinjar, N of Kursi	Iraq (Northern)
W29	KU8807	North slope of Jabal Sinjar, N of Kursi	Iraq (Northern)
W30	KU8808	North slope of Jabal Sinjar, N of Kursi	Iraq (Northern)
W31	KU8809	North slope of Jabal Sinjar, N of Kursi	Iraq (Northern)
W32	KU8810	North slope of Jabal Sinjar, N of Kursi	Iraq (Northern)
W33	KU8811	North slope of Jabal Sinjar, N of Kursi	Iraq (Northern)
W34	KU8812	North slope of Jabal Sinjar, N of Kursi	Iraq (Northern)
W35	KU8814	North slope of Jabal Sinjar, N of Kursi	Iraq (Northern)
W36	KU8815	North slope of Jabal Sinjar, N of Kursi	Iraq (Northern)
W37	KU8816A	North slope of Jabal Sinjar, N of Kursi	Iraq (Northern)
W38	KU8816B	North slope of Jabal Sinjar, N of Kursi	Iraq (Northern)
W39	KU8817	North slope of Jabal Sinjar, N of Kursi	Iraq (Northern)
W40	KU8821A	15.3 km ENE from Dohuk to Amadiyah	Iraq (Northern)
W41	KU8821C	15.3 km ENE from Dohuk to Amadiyah	Iraq (Northern)
W42	KU8915A	17.3 km E from Silvan to Bitlis	Turkey (Karacadağ region near Diyarbakir)
W43	KU8915B	17.3 km E from Silvan to Bitlis	Turkey (Karacadağ region near Diyarbakir)
W44	KU8935	9.3 km SE from Ergani to Diyarbakir	Turkey (Karacadağ region near Diyarbakir)
W45	KU8937B	9.3 km SE from Ergani to Diyarbakir	Turkey (Karacadağ region near Diyarbakir)
W46	KU8941	58.8 km N from Kermanshah to Ravansar	Iran
W47	KU8942	58.8 km N from Kermanshah to Ravansar	Iran
W48	KU8943	58.8 km N from Kermanshah to Ravansar	Iran
W49	KU13441	Near Korazim Junction, Upper Jordan Valley	Israel (Upper Jordan Valley and Mt. Hermon)

(to be continued)

Supplemental Table 1. (continued)

Accession No. in this study	Accession No. at origin [a]	Collection localities	Countries (Regions)
W50	KU13442	W of Almagor, UpperJordan Valley	Israel (Upper Jordan Valley and Mt. Hermon)
W51	KU13443	Lower Galilee, near Amnun	Israel (Upper Jordan Valley and Mt. Hermon)
W52	KU13444	Between Almagor and Capernaum, Upper Jordan Valley	Israel (Upper Jordan Valley and Mt. Hermon)
W53	KU13445	N of Almagor, Upper Jordan Valley	Israel (Upper Jordan Valley and Mt. Hermon)
W54	KU13446	Between Almagor and Capernaum, Upper Jordan Valley	Israel (Upper Jordan Valley and Mt. Hermon)
W55	KU13447	Har Yaarran, Judean Mountains	Israel (Judean Mountains and foothills)
W56	KU13448	Matta, Judean Mountains	Israel (Judean Mountains and foothills)
W57	KU13449	Bar Giyyora, Judean Mountains	Israel (Judean Mountains and foothills)
W58	KU13450	Nevo Betar, Judean Mountains	Israel (Judean Mountains and foothills)
W59	KU13451	Nili, Shfelila	Israel (Judean Mountains and foothills)
W60	KU13452	Mattiyahu, Shfeila	Israel (Judean Mountains and foothills)
W61	KU13453	Mattiyahu, Shfeila	Israel (Judean Mountains and foothills)
W62	KU13454	Mount Har Dov	Israel (Upper Jordan Valley and Mt. Hermon)
W63	1993-8-8-1-1	On the hill above Wadi Dederiyeh, NW of Aleppo	Syria
W64	1993-8-16-5-1	9 km from Sirghaya	Syria
W65	KU108-1	unknown	unknown
W66	KU108-3	20 km NW of Suweida (Cheikh Meskine-Suweida)	Syria
W67	KU109	unknown	Israel (unknown)
Domesticated emmer wheat (*T. turgidum* ssp. *dicoccum*)			
D01	KU111	unknown	unknown
D02	KU112	Peiping	China
D03	KU113	unknown	unknown
D04	KU114	unknown	unknown
D05	KU115	unknown	unknown
D06	KU116	unknown	unknown
D07	KU117	unknown	unknown
D08	KU118	unknown	unknown
D09	KU119	unknown	unknown
D10	KU120	unknown	unknown
D11	KU122	unknown	unknown
D12	KU123	unknown	unknown
D13	KU124	unknown	unknown
D14	KU189	unknown	unknown
D15	KU1533	Erevan	Armenia
D16	KU1538	Erevan	Armenia
D17	KU1564	Erevan	Armenia
D18	KU1582	Angaband (Erevan - Airport)	Armenia
D19	KU3371	unknown	Iran
D20	KU3722	unknown	Turkey
D21	KU3723	unknown	Turkey
D22	KU4541	unknown	Iran
D23	KU7301	30 km N of MaiChew, S of Asmara	Ethiopia
D24	KU7302	Debre Markos market, NW of Addis	Ethiopia
D25	KU7303	70 km from Danghela (Bure - Danghela)	Ethiopia
D26	KU7304	76 km NW from Addis Ababa, toward Fiche	Ethiopia
D27	KU7305	Nathi market, 224 km SW of Addis Ababa tward Jimma	Ethiopia
D28	KU7307	Sombo market, 25 km SW of Jimma	Ethiopia
D29	KU7308	5 km NW of Sartomra, NW of Jimma	Ethiopia
D30	KU7310	Addis Ababa market (origin is Akaki, 15 km S of Addis Ababa)	Ethiopia
D31	KU9001	Mt. Entoto, Addis Ababa	Ethiopia
D32	KU9009	Mt. Erer	Ethiopia

(to be continued)

Supplemental Table 1. (continued)

Accession No. in this study	Accession No. at origin [a]	Collection localities	Countries (Regions)
D33	KU9015	21 km from Asbe Tafari to Alemaya	Ethiopia
D34	KU9016	4 km from Kulubi to Deder	Ethiopia
D35	KU9017	12 km from Kulube to Deder	Ethiopia
D36	KU9025	On the west hill of the town of Deder	Ethiopia
D37	KU9028	ca. 10 km from Dessie to Haik	Ethiopia
D38	KU9030	ca. 10 km from Dessie to Haik	Ethiopia
D39	KU9763	Zuquala	Ethiopia
D40	KU9765	Alemaya (Harar)	Ethiopia
D41	KU9768	97 km S of Quiha (near MalChew)	Ethiopia
D42	KU9769	37 km W of Debre Markos	Ethiopia
D43	KU9770	Zuquala	Ethiopia
D44	KU9771	Bale Goba	Ethiopia
D45	KU9772	Near Mt. Yerer	Ethiopia
D46	KU9773	Chercher highland	Ethiopia
D47	KU9774	Goba	Ethiopia
D48	KU9776	46 km NW of Addis Ababa tward Fiche	Ethiopia
D49	KU9777	59 km SW of Jimma (old Omo road)	Ethiopia
D50	KU9778	24 km N of Assella	Ethiopia
D51	KU9779	75 km E of Shashamane tward Goba	Ethiopia
D52	KU9780	Debre Markos market, NW of Addis	Ethiopia
D53	KU9781	Addis Ababa market	Ethiopia
D54	KU9782	Sombo market, 25 km SW of Jimma	Ethiopia
D55	KU9783	Alemaya (Harar)	Ethiopia
D56	KU9784	SE slope of Mt. Yerer	Ethiopia
D57	KU9785	Alemaya (Harar)	Ethiopia
D58	KU9789	100 km NE of Gondar (near Debark)	Ethiopia
D59	KU9791	329 km S of Asmara (near Quiha)	Ethiopia
D60	KU9792	44 km S of Quiha	Ethiopia
D61	KU9793	37 km W of Debre Markos	Ethiopia
D62	KU10490	23.1 km W from Sanandaj to Dezh Shahpur	Iran
D63	KU10492	23.1 km W from Sanandaj to Dezh Shahpur	Iran
D64	KU10497	23.1 km W from Sanandaj to Dezh Shahpur	Iran
D65	KU10500	23.1 km W from Sanandaj to Dezh Shahpur	Iran
D66	KU10501	23.1 km W from Sanandaj to Dezh Shahpur	Iran
D67	KU1023	Valle de Candamo, Oviedo	Spain
D68	KU1056	Piguena de Somiedo, Oviedo	Spain
D69	KU1065	Villar de Vildas de Somiedo, Oviedo	Spain
D70	KU1105	Carrea de Teverga, Oviedo	Spain
D71	KU1108	San Salvador de Teverga, Oviedo	Spain
D72	KU1113	Villa de Sub de Teverga, Oviedo	Spain
D73	KU491	Kallimarai Village, Tamir Nadu	India
D74	KU492	Seed shop at Ootakamund, Nirgiri Hills, Tamir Nadu	India
D75	KU493	Seed shop at Ootakamund, Nirgiri Hills, Tamir Nadu	India
D76	KU494	Seed shop at Ootakamund, Nirgiri Hills, Tamir Nadu	India
D77	KU495	Karimangalam Village, Tamir Nadu	India
D78	KU496	Vellakadai Village, Tamir Nadu	India
D79	2007-Mysore	Supermarket at Mysore, Karnataka	India
D80	2008-10-5-1-1	I. D. Hari, 28 km from Madhugir, Karnataka	India
D81	2008-10-5-1-2	I. D. Hari, 28 km from Madhugir, Karnataka	India
D82	1991-6-24-4E-3	Sovici, ca. 15 km SE of Imotski	Bosnia and Herzegovina

[a] KU, accession numbers of Kyoto University; the other nos., collection numbers of the field surveys conducted by Gifu University, Japan (Furuta and Ohta 1993) and Research Institute for Humanity and Nature, Japan

Supplemental Table 2. The mean one-grain weights (mg) of the three grain groups and the GWR of two-grained spikelets in 60 wild and 37 domesticated emmer wheat accessions examined in 2009, as well as the *t* -test result between the mean one-grain weights of the first and second florets

Accession No.	Two-grained spikelets											One-grained spikelets			
	First florets				Second florets							First florets			
	No. of grains measured	Mean	±	SE	No. of grains measured	Mean	±	SE	*t*		GWR	No. of grains measured	Mean	±	SE
Wild emmer wheat															
W01	20	24.15	±	0.82	20	32.07	±	1.40	4.88	**	0.753	20	30.40	±	1.67
W02	20	17.79	±	0.55	20	25.73	±	1.03	6.77	**	0.691				
W03	20	15.36	±	0.70	20	13.45	±	0.72	1.90		1.142	15	14.34	±	1.01
W04	20	14.28	±	0.35	20	15.15	±	0.63	1.22		0.942				
W05	20	13.10	±	0.52	20	20.36	±	0.69	8.45	**	0.643	20	19.07	±	1.02
W06	20	13.97	±	0.43	20	16.72	±	0.70	3.35	**	0.836	10	17.24	±	1.51
W07	20	16.50	±	0.52	20	20.25	±	1.02	3.28	**	0.815	19	22.33	±	1.47
W08	20	11.00	±	0.29	20	11.87	±	0.48	1.57		0.926				
W09	20	18.22	±	0.80	20	18.22	±	0.80	0.00		1.000				
W10	20	24.61	±	1.02	20	28.64	±	1.29	2.45	*	0.859				
W11	20	12.68	±	0.48	20	14.44	±	0.76	1.95		0.878				
W12	20	18.71	±	0.52	20	16.51	±	1.52	1.37		1.133	18	17.53	±	1.56
W13	20	11.40	±	0.36	20	11.21	±	0.47	0.32		1.017	16	12.30	±	1.28
W14	20	12.89	±	0.36	20	16.20	±	0.57	4.91	**	0.796				
W15	20	14.16	±	0.57	20	14.21	±	0.95	0.05		0.996	10	19.06	±	2.62
W16	20	11.80	±	0.42	20	15.43	±	0.76	4.18	**	0.765	20	13.60	±	0.36
W17	20	21.91	±	0.58	20	25.78	±	1.05	3.21	**	0.850	16	29.41	±	1.72
W18	20	18.43	±	0.43	20	31.27	±	0.85	13.51	**	0.589				
W19	20	17.94	±	0.93	20	30.42	±	1.40	7.45	**	0.590				
W20	20	20.36	±	0.69	20	27.97	±	1.42	4.83	**	0.728				
W21	20	25.26	±	0.91	20	35.19	±	1.41	5.93	**	0.718				
W22	20	22.32	±	0.85	20	33.04	±	0.73	9.57	**	0.675				
W23	20	23.81	±	0.78	20	28.40	±	1.00	3.63	**	0.838				
W25	20	25.03	±	1.02	20	30.29	±	0.87	3.91	**	0.826	19	27.76	±	1.39
W26	9	30.63	±	1.37	10	31.84	±	0.78	0.78		0.962	10	30.65	±	1.23
W27	10	23.59	±	1.09	10	28.29	±	0.99	3.19	**	0.834	10	27.82	±	1.46
W30	10	31.43	±	0.96	10	38.31	±	1.06	4.81	**	0.820	20	38.10	±	0.85
W31	20	29.18	±	0.70	20	37.19	±	0.98	6.66	**	0.785	10	32.46	±	1.47
W32	9	30.53	±	0.72	9	31.62	±	1.13	0.81		0.966	10	32.57	±	1.29
W33	20	27.79	±	1.46	20	32.77	±	1.13	2.70	*	0.848	10	31.49	±	2.27
W35	17	28.02	±	0.56	17	33.55	±	1.19	4.20	**	0.835	10	33.61	±	2.05
W37	20	33.55	±	0.87	20	33.23	±	0.97	0.24		1.010	20	33.86	±	0.87
W39	20	30.51	±	0.87	20	32.47	±	1.04	1.45		0.940				
W40	20	24.00	±	0.75	20	34.16	±	1.13	7.48	**	0.703	18	30.73	±	1.34
W41	20	17.85	±	0.58	20	28.85	±	1.04	9.21	**	0.619				
W42	20	18.76	±	0.29	20	28.57	±	0.63	14.11	**	0.657	15	25.21	±	0.83
W43	20	16.20	±	0.24	20	24.90	±	0.62	13.00	**	0.651	10	21.70	±	1.14
W44	19	22.67	±	1.06	19	31.39	±	0.86	6.40	**	0.722	10	31.96	±	1.95
W45	20	23.06	±	1.73	20	24.84	±	0.71	0.95		0.929				
W46	18	21.53	±	1.13	18	27.16	±	0.89	3.92	**	0.793				
W47	20	22.97	±	0.98	20	29.80	±	0.85	5.27	**	0.771				
W48	20	29.12	±	0.94	20	31.89	±	1.90	1.31		0.913	10	29.57	±	1.69
W49	10	35.91	±	1.21	10	47.87	±	1.22	6.96	**	0.750				
W51	16	27.89	±	1.48	16	39.73	±	1.50	5.61	**	0.702				
W52	10	34.03	±	1.55	10	45.24	±	2.07	4.33	**	0.752				
W53	20	26.19	±	1.36	20	43.72	±	0.99	10.41	**	0.599				
W54	20	28.35	±	1.18	20	39.18	±	1.41	5.91	**	0.723				
W55	20	23.98	±	0.84	20	26.32	±	1.83	1.16		0.911	16	29.44	±	2.50
W56	20	20.67	±	0.81	20	25.83	±	1.10	3.78	**	0.800	16	22.21	±	1.57

(to be continued)

Supplemental Table 2. (continued)

Accession No.	Two-grained spikelets											One-grained spikelets			
	First florets				Second florets							First florets			
	No. of grains measured	Mean	±	SE	No. of grains measured	Mean	±	SE	*t*		GWR	No. of grains measured	Mean	±	SE
W57	20	20.32	±	1.35	20	29.26	±	1.11	5.10	**	0.694				
W58	7	27.83	±	1.34	7	29.76	±	0.76	1.26		0.935	10	34.30	±	1.16
W59	20	20.50	±	0.79	20	22.88	±	0.67	2.30	*	0.896	10	24.40	±	1.10
W60	15	26.88	±	1.01	15	32.31	±	0.86	4.09	**	0.832				
W61	20	19.89	±	0.55	20	30.09	±	1.14	8.09	**	0.661	16	29.33	±	1.30
W62	20	30.10	±	1.27	20	35.84	±	1.77	2.63	*	0.840				
W63	20	23.40	±	0.71	20	33.25	±	0.52	11.23	**	0.704				
W64	20	22.73	±	0.86	20	34.03	±	0.97	8.73	**	0.668				
W65	20	34.73	±	0.94	20	48.04	±	0.91	10.19	**	0.723				
W66	20	26.16	±	0.35	20	37.51	±	1.14	9.55	**	0.697	10	28.22	±	2.35
W67	20	31.18	±	0.53	20	50.83	±	1.30	13.96	**	0.613	10	48.84	±	3.20
Domesticated emmer wheat															
D01	20	46.77	±	1.54	20	46.76	±	1.42	0.00		1.000	20	44.75	±	1.62
D02	20	56.60	±	1.45	20	54.99	±	1.22	0.85		1.029	17	46.49	±	1.81
D03	20	52.44	±	1.23	20	50.74	±	1.09	1.04		1.034				
D05	20	46.70	±	2.06	20	45.50	±	2.06	0.41		1.026				
D06	20	40.02	±	0.82	20	36.64	±	1.74	1.75		1.092	20	44.92	±	0.75
D07	20	28.31	±	1.17	20	29.92	±	0.77	1.15		0.946				
D08	20	49.85	±	1.15	20	48.40	±	2.53	0.52		1.030				
D09	20	52.88	±	1.27	20	50.70	±	1.25	1.23		1.043	20	44.76	±	1.64
D10	20	47.49	±	1.27	20	48.64	±	1.31	0.63		0.976	10	37.35	±	2.08
D11	20	45.17	±	1.06	20	45.08	±	1.39	0.05		1.002	16	33.84	±	1.63
D12	20	35.94	±	1.41	20	32.53	±	1.48	1.67		1.105	20	31.55	±	0.89
D13	20	30.87	±	0.78	20	28.67	±	1.08	1.66		1.077	10	21.25	±	2.02
D14	20	35.00	±	1.54	20	37.37	±	1.44	1.12		0.937	20	36.44	±	0.75
D15	20	22.22	±	1.09	20	17.95	±	1.09	2.76	**	1.238	20	17.02	±	0.92
D16	20	20.28	±	0.47	20	17.57	±	0.68	3.28	**	1.154	20	17.40	±	0.51
D17	20	28.68	±	1.83	20	25.24	±	1.91	1.30		1.136	17	20.49	±	0.87
D19	20	19.69	±	1.00	20	15.05	±	1.01	3.27	**	1.309	20	16.79	±	0.84
D20	20	50.66	±	1.18	20	50.96	±	1.60	0.15		0.994				
D21	20	48.74	±	1.58	20	47.67	±	1.47	0.50		1.022				
D23	20	44.17	±	0.55	20	39.88	±	0.93	3.97	**	1.108	10	41.69	±	1.91
D38	20	47.90	±	0.83	20	46.62	±	1.14	0.91		1.027	17	40.75	±	1.39
D62	20	28.95	±	1.41	20	25.37	±	1.25	1.90		1.141	15	25.19	±	1.14
D63	20	19.19	±	0.49	20	16.35	±	0.49	4.10	**	1.173	20	15.33	±	0.65
D64	20	30.11	±	2.01	20	26.28	±	2.31	1.25		1.146	18	20.37	±	1.32
D65	20	29.67	±	0.58	20	23.10	±	0.72	7.13	**	1.284	20	21.92	±	0.96
D66	20	30.16	±	1.03	20	24.22	±	1.26	3.64	**	1.245	16	21.66	±	1.49
D67	20	39.67	±	0.72	20	34.73	±	0.56	5.43	**	1.142				
D68	20	39.07	±	2.37	20	35.72	±	2.12	1.05		1.094	16	24.13	±	2.03
D69	20	54.26	±	3.05	20	50.40	±	1.98	1.06		1.077	16	42.46	±	4.04
D72	20	60.79	±	0.78	20	58.15	±	1.00	2.08	*	1.045	10	47.35	±	2.23
D73	20	44.89	±	1.53	20	48.10	±	1.56	1.47		0.933	20	40.21	±	1.37
D77	20	45.93	±	0.81	20	46.51	±	0.91	0.48		0.988	10	41.91	±	1.72
D78	20	43.74	±	1.07	20	43.04	±	1.41	0.39		1.016	20	44.34	±	0.52
D79	20	52.26	±	0.92	20	51.16	±	1.78	0.55		1.022	18	45.07	±	1.48
D80	20	47.60	±	1.58	20	48.57	±	1.55	0.44		0.980	20	37.53	±	1.61
D81	20	53.71	±	1.08	20	52.49	±	1.24	0.74		1.023				
D82	20	42.98	±	1.66	20	40.38	±	1.75	1.08		1.065	18	30.02	±	1.95

* and ** indicate the mean one-grain weights of the first and second florets significantly differ from each other at p = 0.05 and p = 0.01, respectively

Supplemental Table 3. One-grain weight of the three grain groups and GWR of two-grained spikelets in wild and domesticated emmer wheat accessions examined in 2010 and 2014

Accession No.	2010							2014 Field							2014 Greenhouse						
	Two-grained spikelets					One-grained spikelets		Two-grained spikelets					One-grained spikelets		Two-grained spikelets					One-grained spikelets	
	First florets		Second florets			First florets		First florets		Second florets			First florets		First florets		Second florets			First florets	
	No. of grains measured	One-grain weight	No. of grains measured	One-grain weight	GWR	No. of grains measured	One-grain weight	No. of grains measured	One-grain weight	No. of grains measured	One-grain weight	GWR	No. of grains measured	One-grain weight	No. of grains measured	One-grain weight	No. of grains measured	One-grain weight	GWR	No. of grains measured	One-grain weight
Wild emmer wheat																					
W01	10	21.31	10	35.17	0.606	10	33.61	50	23.81	50	32.46	0.734	16	29.04	25	22.67	25	30.89	0.734	12	25.93
W02	10	16.13	10	22.21	0.726	10	17.25	50	22.68	50	33.21	0.683	21	29.47	22	15.38	22	22.44	0.686	22	19.97
W03	10	16.11	10	21.01	0.767	10	20.88	50	22.79	50	29.23	0.780			25	11.46	25	19.19	0.597	25	14.79
W04	10	15.76	10	18.57	0.849	10	16.61	50	19.37	50	31.04	0.624	11	27.85	50	13.20	50	20.81	0.634	16	15.37
W05	10	14.72	10	21.33	0.690	10	20.38	50	21.50	50	31.55	0.681	25	32.42	20	16.51	20	22.58	0.731	25	22.69
W06	10	11.40	10	16.49	0.691	10	14.54	50	21.24	50	31.00	0.685			50	13.63	50	21.36	0.638		
W07	10	14.49	10	23.74	0.610	10	21.94	49	25.17	50	35.70	0.705			16	19.08	16	27.49	0.694	25	33.99
W08	10	13.92	10	20.08	0.693			50	17.89	50	26.18	0.683			50	11.85	50	15.00	0.790	18	14.60
W09	10	15.04	10	21.15	0.711	10	23.46														
W10	10	16.54	10	23.73	0.697	10	24.43	50	23.88	50	33.72	0.708			25	16.07	25	23.33	0.689	25	25.95
W11	10	12.82	10	17.50	0.733	10	15.49	50	22.37	50	33.32	0.671			50	12.57	50	18.16	0.692	25	15.99
W12	10	14.63	10	21.30	0.687	10	17.68	50	22.66	50	32.40	0.699			25	12.22	25	17.26	0.708	25	12.31
W13	10	12.50	10	17.34	0.721	10	15.40	50	21.73	49	31.47	0.690	10	28.36	50	14.76	50	21.05	0.701		
W14	10	12.21	10	16.49	0.740	10	15.38	50	22.50	50	29.95	0.751	15	28.11	50	13.79	50	19.99	0.690	12	14.47
W15	10	12.78	10	18.94	0.675	10	16.48	50	21.44	50	33.17	0.646	17	31.99	25	16.81	25	25.54	0.658	25	25.79
W16	10	13.65	10	19.60	0.696	10	14.94	25	21.52	25	29.79	0.722	24	28.63	50	12.04	50	17.95	0.671	13	13.26
W17	10	15.29	10	21.10	0.725	10	19.00	50	20.35	50	31.58	0.644			50	16.08	50	25.80	0.623	22	17.46
W18	10	18.74	10	30.06	0.623	10	25.92	50	25.11	50	37.98	0.661			50	18.80	50	28.74	0.654	12	18.71
W19	10	16.33	10	27.50	0.594	10	20.64	50	27.46	50	41.22	0.666			50	18.81	50	30.31	0.621	18	17.83
W20	10	18.70	10	27.50	0.680	10	21.94	50	27.30	50	41.24	0.662			50	20.07	50	31.84	0.630	19	24.74
W21	10	17.04	10	28.75	0.593	10	22.80	50	27.45	50	41.81	0.657	11	29.71	50	19.10	50	31.99	0.597	25	20.38
W22	10	18.17	10	25.50	0.713	10	16.32	50	24.46	50	39.48	0.620	11	33.17	25	19.75	25	29.66	0.666	24	22.50
W23	10	19.14	10	25.25	0.758	10	21.23	50	22.65	50	32.40	0.699	11	28.67	50	17.83	50	24.87	0.717	20	19.19
W24	10	15.15	10	19.50	0.777	10	17.27														
W25	10	18.46	10	22.55	0.819	10	23.27	50	22.73	50	32.62	0.697	13	24.92	50	18.26	50	23.65	0.772	21	19.29
W26	10	20.45	10	32.21	0.635	10	28.09	50	24.22	50	39.87	0.607	10	29.22	50	14.11	50	26.57	0.531	25	21.55
W27	10	19.40	10	30.44	0.637			50	24.21	50	38.85	0.623	11	28.58	50	14.82	49	27.22	0.544	21	16.77
W28	10	20.43	10	31.54	0.648	10	25.39														
W29	10	23.79	10	27.18	0.875	10	29.23														
W30	10	21.87	10	27.79	0.787	10	31.06	50	24.07	50	38.99	0.617	15	30.21	25	17.55	25	27.96	0.628	23	24.93
W31	10	22.42	10	31.20	0.719	10	25.18	50	30.27	50	47.68	0.635	17	40.93	25	23.72	25	35.14	0.675	25	30.13
W32	10	21.78	10	27.30	0.798	10	21.51	50	23.42	50	32.53	0.720	13	27.68	25	18.88	25	31.01	0.609	25	24.05
W33	10	19.20	10	31.94	0.601	10	23.03	50	25.24	50	35.06	0.720	16	31.14	50	17.43	50	30.96	0.563	24	24.98
W34	10	16.93	10	26.82	0.631	10	23.45														
W35	10	21.70	10	33.86	0.641	10	25.74														
W36	10	24.77	10	33.54	0.739	10	28.02														
W37	10	25.53	10	27.81	0.918	10	28.30	50	32.33	50	45.25	0.714	20	39.95	25	22.73	25	30.04	0.757	25	34.45
W38	10	23.16	10	28.96	0.800	10	23.53														
W39	10	15.39	10	26.77	0.575	10	20.00	48	22.86	50	38.17	0.599	15	25.99	25	14.50	25	25.79	0.562	14	15.59
W40	10	16.80	10	23.56	0.713	10	16.47	50	21.81	50	37.77	0.577			23	16.46	22	27.98	0.588	25	17.85

(to be continued)

Supplemental Table 3. (continued)

Accession No.	2010 Two-grained spikelets First florets No. of grains measured	One-grain weight	Second florets No. of grains measured	One-grain weight	GWR	2010 One-grained spikelets First florets No. of grains measured	One-grain weight	2014 Field Two-grained spikelets First florets No. of grains measured	One-grain weight	Second florets No. of grains measured	One-grain weight	GWR	2014 Field One-grained spikelets First florets No. of grains measured	One-grain weight	2014 Greenhouse Two-grained spikelets First florets No. of grains measured	One-grain weight	Second florets No. of grains measured	One-grain weight	GWR	2014 Greenhouse One-grained spikelets First florets No. of grains measured	One-grain weight
W41	10	19.75	10	26.76	0.738	10	30.41	50	20.94	50	36.05	0.581	11	28.03	25	17.47	25	28.72	0.608	21	16.79
W42	10	15.85	10	23.81	0.666	10	19.75	50	17.86	49	30.11	0.593	25	26.94	25	13.62	25	23.66	0.576	25	19.02
W43															33	13.90	33	21.88	0.635	24	20.78
W44	10	25.79	10	27.72	0.930	10	30.04	50	23.38	50	33.36	0.701	9	29.94	25	13.06	25	18.41	0.709	25	17.73
W45	10	24.38	10	22.73	1.073	10	23.06	50	20.14	50	30.03	0.671	18	27.86	18	17.15	18	21.61	0.794	25	25.62
W46	10	16.21	10	21.60	0.750	10	20.78	25	8.57	25	10.68	0.803	11	11.09	24	13.63	24	23.70	0.575		
W47	10	18.93	10	25.54	0.741	10	19.27	25	9.74	25	15.41	0.632	10	16.91	25	14.15	25	24.47	0.578	10	14.12
W48	10	22.38	10	28.07	0.797	10	23.53	50	10.76	50	13.15	0.818	16	13.08	50	15.68	50	26.65	0.589		
W49	10	28.80	10	45.04	0.639			50	31.09	50	47.44	0.655			25	32.07	25	46.64	0.688	10	43.41
W50	10	23.43	10	40.85	0.574	10	24.19														
W51	10	19.85	10	34.83	0.570	10	22.98	50	25.96	50	43.79	0.593			17	27.23	17	49.88	0.546	19	50.12
W52	10	22.13	10	35.39	0.625			50	26.85	49	45.58	0.589	13	32.50	25	32.18	25	45.26	0.711	19	40.41
W53	10	22.17	10	36.67	0.605	10	24.51	50	24.48	50	43.19	0.567			25	23.92	25	41.99	0.570		
W54	10	23.56	10	35.10	0.671	10	22.74	50	26.71	50	44.93	0.594			25	22.95	25	40.68	0.564	16	38.69
W55	10	19.75	10	25.18	0.784	10	18.73	50	22.20	50	32.15	0.690			50	20.08	50	28.15	0.713		
W56	10	21.20	10	32.31	0.656			50	22.36	50	28.94	0.773			50	22.80	50	29.37	0.776		
W57	10	15.82	10	19.20	0.824	10	21.04	49	20.76	50	30.00	0.692									
W58	10	24.93	10	23.18	1.075	10	27.26	25	18.14	25	23.11	0.785	20	22.67							
W59	10	14.58	10	23.14	0.630	10	21.48	50	17.01	50	26.03	0.653	10	24.08							
W60	10	21.65	10	27.38	0.791	10	28.13	50	25.06	50	39.96	0.627	10	30.64	25	20.60	25	34.63	0.595	22	30.24
W61	10	14.96	10	24.76	0.604	10	27.70	50	17.22	51	30.19	0.570									
W62	10	21.66	10	31.15	0.695	10	21.79	50	27.41	50	40.11	0.683	13	28.32							
W63	10	17.80	10	26.65	0.668	10	23.85	50	22.64	50	36.02	0.629			25	18.79	25	25.98	0.723	12	26.74
W64	10	22.75	10	32.04	0.710	10	30.78	50	24.22	50	38.02	0.637	14	37.46	25	20.04	25	26.24	0.764	19	29.69
W65	10	27.30	10	42.46	0.643	10	33.47	49	30.44	50	46.43	0.656	11	41.98	14	22.86	14	35.80	0.639	17	32.84
W66	10	22.51	10	31.11	0.724	10	31.39	50	26.38	50	40.69	0.648			50	21.13	50	34.48	0.613		
W67	10	20.58	10	34.90	0.590			50	23.90	50	43.23	0.553			25	20.14	25	36.75	0.548		
Domesticated emmer wheat																					
D01								50	45.39	50	45.38	1.000	14	38.59	50	38.38	50	37.18	1.032	11	28.44
D02	10	45.70	10	35.58	1.284	10	36.70	50	47.77	50	46.61	1.025			50	45.45	50	44.26	1.027		
D03	10	41.64	10	42.32	0.984	10	35.40	50	47.71	50	49.72	0.959			50	43.39	50	45.59	0.952		
D04	10	30.08	10	25.68	1.171	10	23.39														
D05	10	40.83	10	40.98	0.996	10	32.94	50	52.83	50	54.63	0.967	12	54.38	25	42.25	25	41.95	1.007	50	40.25
D06	10	35.70	10	31.57	1.131	10	32.29	50	47.86	50	48.15	0.994	25	45.04	50	36.19	50	32.49	1.114	50	33.60
D07	10	34.46	10	32.69	1.054	10	30.63	50	36.72	50	36.54	1.005	13	27.77	50	27.59	50	25.91	1.065	23	18.90
D08	10	21.58	10	16.14	1.337	10	20.51	50	55.50	50	56.33	0.985	23	49.77	25	45.90	25	41.13	1.116	50	42.06
D09								50	54.31	50	56.22	0.966	50	50.06	50	51.71	50	49.49	1.045	25	49.62
D10	10	43.25	10	36.20	1.195	10	37.84	50	44.92	50	45.41	0.989			50	39.54	50	41.10	0.962	14	32.88
D11								50	43.73	50	44.31	0.987			50	46.58	50	46.14	1.010	25	42.21
D12								25	48.74	25	48.64	1.002	13	49.31	50	42.84	50	38.13	1.123	50	43.07

(to be continued)

Supplemental Table 3. (continued)

Accession No.	2010 Two-grained spikelets First florets No. of grains measured	2010 Two-grained spikelets First florets One-grain weight	2010 Two-grained spikelets Second florets No. of grains measured	2010 Two-grained spikelets Second florets One-grain weight	2010 Two-grained spikelets GWR	2010 One-grained spikelets First florets No. of grains measured	2010 One-grained spikelets First florets One-grain weight	2014 Field Two-grained spikelets First florets No. of grains measured	2014 Field Two-grained spikelets First florets One-grain weight	2014 Field Two-grained spikelets Second florets No. of grains measured	2014 Field Two-grained spikelets Second florets One-grain weight	2014 Field Two-grained spikelets GWR	2014 Field One-grained spikelets First florets No. of grains measured	2014 Field One-grained spikelets First florets One-grain weight	2014 Greenhouse Two-grained spikelets First florets No. of grains measured	2014 Greenhouse Two-grained spikelets First florets One-grain weight	2014 Greenhouse Two-grained spikelets Second florets No. of grains measured	2014 Greenhouse Two-grained spikelets Second florets One-grain weight	2014 Greenhouse Two-grained spikelets GWR	2014 Greenhouse One-grained spikelets First florets No. of grains measured	2014 Greenhouse One-grained spikelets First florets One-grain weight
D13	10	16.19	10	16.90	0.958	10	15.89	50	43.27	50	42.26	1.024	10	38.95	50	34.10	50	33.62	1.014	23	29.61
D14								50	43.75	50	42.24	1.036			50	34.67	50	32.69	1.061	25	25.07
D15	10	22.60	10	21.56	1.048	10	22.01	50	35.39	50	35.81	0.988	18	27.25	50	28.15	50	26.05	1.080	25	26.36
D16								50	38.61	50	38.31	1.008			25	29.46	25	29.29	1.006	25	25.36
D17																					
D18	10	17.65	10	17.00	1.038			50	46.97	50	47.43	0.990			50	31.32	50	29.60	1.058	13	24.92
D19	10	27.00	10	22.00	1.227	10	24.17	50	38.66	50	38.06	1.016	19	40.71							
D20	10	31.47	10	23.70	1.328	10	35.36														
D21	10	41.45	10	37.06	1.118	10	34.93	50	45.62	50	47.27	0.965			50	43.47	50	43.59	0.997	14	34.63
D22	10	22.70	10	21.11	1.075	10	22.65														
D23	10	23.87	10	31.49	0.758	10	24.64														
D24	10	31.76	10	31.56	1.006	10	31.67														
D25	10	22.03	10	25.54	0.863	10	22.11														
D26	10	39.45	10	35.99	1.096	10	29.46								24	39.86	24	39.06	1.020	15	39.61
D27	10	46.55	10	37.69	1.235	10	35.23														
D28	10	40.86	10	39.15	1.044	10	36.70														
D29	10	41.06	10	35.48	1.157	10	35.85														
D30	10	45.32	10	42.98	1.054	10	37.52														
D31	10	34.43	10	33.98	1.013	10	30.80														
D32	10	44.37	10	40.04	1.108	10	38.30														
D33	10	40.22	10	39.25	1.025	10	33.04														
D34	10	41.11	10	34.04	1.208	10	32.97														
D35	10	41.93	10	42.22	0.993	10	37.32														
D36	10	39.53	10	35.27	1.121	10	36.16														
D37	10	44.02	10	45.99	0.957	10	43.90														
D38								50	42.78	50	42.48	1.007	19	40.18	50	44.46	50	40.27	1.104	14	39.56
D39	10	37.20	10	32.76	1.136	10	33.82														
D40	10	42.70	10	39.36	1.085	10	40.28														
D41	10	45.20	10	42.81	1.056	10	43.59														
D42	10	42.14	10	40.73	1.035	10	39.54														
D43	10	36.45	10	39.01	0.934	10	30.82														
D44	10	46.62	10	38.73	1.204	10	38.97														
D45	10	34.56	10	29.80	1.160	10	32.35														
D46	10	44.36	10	40.87	1.085	10	35.34														
D47	10	44.70	10	37.31	1.198	10	36.43														
D48	10	41.55	10	38.41	1.082	10	38.96														
D49	10	36.33	10	34.52	1.052	10	32.11														
D50	10	37.57	10	35.97	1.044	10	33.30														
D51	10	39.76	10	38.68	1.028	10	40.31														
D52	10	31.71	10	29.26	1.084	10	26.79														

(to be continued)

Supplemental Table 3. (continued)

Accession No.	2010							2014 Field							2014 Greenhouse						
	Two-grained spikelets					One-grained spikelets		Two-grained spikelets					One-grained spikelets		Two-grained spikelets					One-grained spikelets	
	First florets		Second florets			First florets		First florets		Second florets			First florets		First florets		Second florets			First florets	
	No. of grains measured	One-grain weight	No. of grains measured	One-grain weight	GWR	No. of grains measured	One-grain weight	No. of grains measured	One-grain weight	No. of grains measured	One-grain weight	GWR	No. of grains measured	One-grain weight	No. of grains measured	One-grain weight	No. of grains measured	One-grain weight	GWR	No. of grains measured	One-grain weight
D53	10	39.56	10	34.75	1.138	10	32.70														
D54	10	38.54	10	34.31	1.123	10	37.45														
D55	10	44.69	10	39.13	1.142	10	38.49														
D56	10	41.31	10	36.06	1.146	10	32.14														
D57	10	36.11	10	40.44	0.893	10	34.77														
D58	10	37.67	10	34.18	1.102	10	30.65														
D59	10	44.91	10	43.36	1.036	10	42.08														
D60	10	47.24	10	40.98	1.153	10	40.61														
D61	10	34.20	10	29.47	1.161	10	27.42														
D62	10	25.46	10	20.73	1.228	10	24.86	50	37.24	50	37.30	0.998	15	37.87	50	34.36	50	30.62	1.122	25	30.92
D63								50	35.03	50	33.50	1.045	15	30.57	25	33.26	25	29.19	1.139	25	29.83
D64								50	35.91	50	34.45	1.042	23	30.71	50	42.97	50	37.66	1.141	25	36.44
D65								50	39.63	50	37.05	1.070	25	36.85	50	32.11	50	27.28	1.177	25	26.62
D66								50	38.57	50	37.82	1.020	15	34.43	50	39.00	50	33.98	1.148	25	31.91
D67								50	55.97	50	56.17	0.996	50	58.26	50	56.36	50	52.72	1.069	25	48.07
D68	10	48.33	10	50.55	0.956	10	46.63	50	59.89	50	57.26	1.046	25	50.52	50	56.76	50	54.27	1.046	25	49.28
D69	10	39.64	10	33.16	1.195	10	35.53	50	61.64	50	58.90	1.046	50	58.86	50	55.64	50	54.21	1.026	25	51.58
D70	10	34.18	10	33.28	1.027	10	30.12														
D71	10	43.53	10	41.48	1.049	10	38.74														
D72	10	45.32	10	51.63	0.878	10	42.97	50	59.47	50	62.38	0.953	21	56.22	50	61.50	50	61.19	1.005	17	48.72
D73	10	32.23	10	33.27	0.969	10	32.72	50	45.35	50	48.30	0.939			50	38.44	50	39.70	0.968	11	25.26
D74	10	44.87	10	40.26	1.115	10	38.98														
D75	10	34.70	10	27.98	1.240	10	29.52														
D76	10	35.24	10	32.68	1.078	10	26.44														
D77	10	47.14	10	39.18	1.203	10	39.74	50	45.78	50	45.72	1.001			50	42.59	50	41.90	1.017		
D78	10	31.78	10	28.56	1.113	10	30.92	50	39.48	50	40.25	0.981	17	39.01	50	37.26	50	34.83	1.070	15	26.43
D79	10	39.93	10	41.25	0.968	10	32.45	50	47.69	50	49.92	0.955			50	47.31	50	49.21	0.961		
D80	10	42.34	10	36.73	1.153	10	26.96	50	46.56	50	47.16	0.987			50	40.03	50	37.72	1.061	10	28.72
D81	10	40.24	10	39.67	1.014	10	30.07	50	45.21	50	44.66	1.012			50	38.36	50	39.20	0.979		
D82	10	35.69	10	31.49	1.133	10	28.01	50	58.01	50	57.21	1.014	16	52.28	50	49.23	50	47.57	1.035	20	39.20

Supplemental Table 4. GR and GI values of the three grain groups in wild and domesticated emmer wheat accessions

Accession No.	2009																	
	Sown on September 6									Sown on November 6								
	Two-grained spikelets						One-grained spikelets			Two-grained spikelets						One-grained spikelets		
	First florets			Second florets			First florets			First florets			Second florets			First florets		
	No. of grains sown	GR	GI	No. of grains sown	GR	GI	No. of grains sown	GR	GI	No. of grains sown	GR	GI	No. of grains sown	GR	GI	No. of grains sown	GR	GI
Wild emmer wheat																		
W01	10	0.80	0.78	10	1.00	1.00	10	1.00	1.00	10	1.00	0.95	10	1.00	1.00	10	1.00	1.00
W02	10	0.20	0.12	10	1.00	0.99	5	1.00	0.96	10	0.00	0.00	10	1.00	1.00	-	-	-
W03	10	1.00	0.93	10	1.00	0.97	10	1.00	0.98	10	1.00	1.00	10	1.00	1.00	5	1.00	1.00
W04	10	0.40	0.37	10	1.00	1.00	8	0.75	0.69	10	0.80	0.72	10	1.00	1.00	-	-	-
W05	10	0.00	0.00	10	1.00	0.95	10	1.00	0.94	10	0.00	0.00	10	1.00	1.00	10	1.00	1.00
W06	10	0.10	0.10	10	1.00	0.96	10	1.00	0.94	10	0.50	0.43	10	1.00	1.00	-	-	-
W07	10	0.60	0.55	10	1.00	1.00	10	1.00	1.00	10	1.00	0.99	10	1.00	1.00	9	1.00	1.00
W08	10	0.20	0.18	10	1.00	0.93	5	0.80	0.70	10	0.40	0.39	10	1.00	1.00	-	-	-
W09	10	0.50	0.40	10	1.00	0.99	8	1.00	0.98	10	1.00	1.00	10	1.00	1.00	-	-	-
W10	10	1.00	0.96	10	1.00	1.00	9	1.00	1.00	10	1.00	1.00	10	1.00	1.00	-	-	-
W11	10	0.10	0.07	10	1.00	0.98	-	-	-	10	0.80	0.77	10	1.00	1.00	-	-	-
W12	10	0.80	0.73	10	1.00	0.98	10	1.00	0.98	10	1.00	1.00	10	1.00	1.00	8	0.88	0.88
W13	10	0.30	0.21	10	1.00	0.92	10	0.90	0.83	10	1.00	1.00	10	1.00	1.00	6	1.00	0.98
W14	10	0.00	0.00	10	1.00	0.99	6	0.83	0.73	10	0.90	0.88	10	1.00	1.00	-	-	-
W15	10	0.90	0.85	10	1.00	0.99	10	1.00	0.99	10	1.00	1.00	10	1.00	1.00	-	-	-
W16	10	0.10	0.08	10	1.00	1.00	10	0.90	0.89	10	0.60	0.59	10	1.00	1.00	10	1.00	1.00
W17	10	0.40	0.31	10	1.00	1.00	10	1.00	1.00	10	0.70	0.69	10	1.00	1.00	6	1.00	1.00
W18	10	0.00	0.00	10	1.00	1.00	10	0.50	0.47	10	0.00	0.00	10	1.00	1.00	-	-	-
W19	10	0.00	0.00	10	1.00	1.00	7	0.29	0.27	10	0.30	0.28	10	1.00	1.00	-	-	-
W20	10	0.00	0.00	10	1.00	1.00	9	0.89	0.87	10	0.10	0.09	10	1.00	1.00	-	-	-
W21	10	0.10	0.09	10	1.00	0.99	-	-	-	10	0.50	0.44	10	1.00	1.00	-	-	-
W22	10	0.20	0.18	10	1.00	1.00	-	-	-	10	0.70	0.66	10	1.00	1.00	-	-	-
W23	10	0.80	0.78	10	0.90	0.90	8	1.00	1.00	10	1.00	1.00	10	1.00	1.00	-	-	-
W24	-	-	-	-	-	-	-	-	-	-	-	-	-	-	-	-	-	-
W25	10	1.00	0.96	10	1.00	1.00	10	1.00	0.99	-	-	-	-	-	-	-	-	-
W26	10	0.80	0.79	9	1.00	1.00	10	1.00	1.00	-	-	-	-	-	-	-	-	-
W27	10	0.20	0.19	10	1.00	1.00	10	1.00	1.00	10	0.90	0.88	10	1.00	1.00	10	1.00	1.00
W28	-	-	-	-	-	-	-	-	-	-	-	-	-	-	-	-	-	-
W29	-	-	-	-	-	-	-	-	-	-	-	-	-	-	-	-	-	-
W30	10	0.20	0.05	10	1.00	1.00	10	1.00	1.00	-	-	-	-	-	-	10	1.00	1.00
W31	10	0.00	0.00	10	1.00	1.00	10	1.00	1.00	10	0.30	0.30	10	1.00	1.00	-	-	-
W32	9	1.00	1.00	9	1.00	1.00	10	1.00	1.00	-	-	-	-	-	-	-	-	-
W33	10	0.40	0.33	10	1.00	1.00	10	1.00	1.00	10	1.00	0.96	10	1.00	1.00	-	-	-
W34	-	-	-	-	-	-	-	-	-	-	-	-	-	-	-	-	-	-
W35	10	0.00	0.00	10	1.00	1.00	10	1.00	1.00	7	0.29	0.24	7	1.00	1.00	-	-	-
W36	-	-	-	-	-	-	-	-	-	-	-	-	-	-	-	-	-	-
W37	10	0.60	0.60	10	1.00	1.00	10	1.00	1.00	10	1.00	1.00	10	1.00	1.00	10	1.00	1.00
W38	-	-	-	-	-	-	-	-	-	-	-	-	-	-	-	-	-	-
W39	10	0.60	0.59	10	1.00	1.00	7	1.00	1.00	10	0.90	0.90	10	1.00	1.00	-	-	-
W40	10	0.00	0.00	10	1.00	1.00	10	0.90	0.90	10	0.50	0.45	10	1.00	1.00	8	1.00	1.00
W41	10	0.00	0.00	10	1.00	1.00	9	0.89	0.87	10	0.30	0.28	10	1.00	1.00	-	-	-
W42	10	0.00	0.00	10	1.00	1.00	10	0.80	0.77	10	0.00	0.00	10	1.00	1.00	5	1.00	1.00
W43	10	0.00	0.00	10	1.00	1.00	10	1.00	1.00	10	0.00	0.00	10	1.00	1.00	-	-	-
W44	10	0.30	0.29	10	1.00	1.00	10	0.90	0.90	9	0.67	0.66	9	1.00	1.00	-	-	-
W45	10	0.40	0.37	10	1.00	1.00	-	-	-	10	0.90	0.88	10	1.00	1.00	-	-	-
W46	10	0.60	0.58	10	1.00	1.00	-	-	-	8	0.75	0.74	8	1.00	1.00	-	-	-
W47	10	0.00	0.00	10	1.00	1.00	8	1.00	1.00	10	0.10	0.09	10	1.00	1.00	-	-	-
W48	10	1.00	0.99	10	1.00	1.00	10	1.00	1.00	10	1.00	1.00	10	1.00	1.00	-	-	-
W49	10	0.00	0.00	10	1.00	0.85	5	1.00	0.88	-	-	-	-	-	-	-	-	-
W50	-	-	-	-	-	-	-	-	-	-	-	-	-	-	-	-	-	-
W51	10	0.50	0.43	10	1.00	0.85	9	1.00	0.87	6	0.67	0.65	6	1.00	1.00	-	-	-
W52	10	0.40	0.27	10	1.00	0.89	-	-	-	-	-	-	-	-	-	-	-	-
W53	10	0.00	0.00	10	1.00	0.89	-	-	-	10	0.60	0.57	10	1.00	1.00	-	-	-
W54	10	0.60	0.46	10	1.00	0.90	7	1.00	0.90	10	0.90	0.90	10	1.00	1.00	-	-	-
W55	10	0.60	0.47	10	1.00	1.00	10	1.00	0.97	10	1.00	0.95	10	1.00	1.00	6	1.00	1.00
W56	10	0.00	0.00	10	1.00	0.94	10	0.90	0.86	10	0.10	0.09	10	1.00	1.00	6	1.00	1.00
W57	10	0.00	0.00	10	1.00	1.00	8	1.00	0.96	10	0.60	0.56	10	1.00	1.00	-	-	-
W58	7	1.00	0.99	7	1.00	1.00	10	1.00	1.00	-	-	-	-	-	-	-	-	-
W59	10	0.60	0.51	10	1.00	0.99	10	1.00	0.97	10	0.70	0.68	10	1.00	1.00	-	-	-
W60	10	0.00	0.00	10	1.00	1.00	7	1.00	1.00	5	0.60	0.56	5	1.00	1.00	-	-	-
W61	10	0.10	0.07	10	1.00	0.93	10	1.00	0.89	10	0.10	0.10	10	1.00	1.00	6	1.00	1.00
W62	10	0.50	0.50	10	1.00	1.00	9	0.89	0.89	10	0.90	0.90	10	1.00	1.00	-	-	-
W63	10	0.70	0.56	10	1.00	1.00	8	1.00	0.98	10	1.00	0.96	10	1.00	1.00	-	-	-
W64	10	0.00	0.00	10	1.00	1.00	8	0.63	0.59	10	0.00	0.00	10	1.00	1.00	-	-	-
W65	10	0.00	0.00	10	1.00	0.99	8	1.00	0.95	10	0.10	0.09	10	1.00	1.00	-	-	-
W66	10	0.00	0.00	10	1.00	1.00	10	1.00	0.98	10	0.60	0.55	10	1.00	1.00	-	-	-
W67	10	0.00	0.00	10	1.00	1.00	10	1.00	0.99	10	0.00	0.00	10	1.00	1.00	-	-	-

Accession No.	2010									2014 (sown on September 9)																	
	Sown on September 19									Grown at field									Grown at greenhouse								
	Two-grained spikelets						One-grained spikelets			Two-grained spikelets						One-grained spikelets			Two-grained spikelets						One-grained spikelets		
	First florets			Second florets			First florets			First florets			Second florets			First florets			First florets			Second florets			First florets		
	No. of grains sown	GRs	GIs	No. of grains sown	GRs	GIs	No. of grains sown	GRs	GIs	No. of grains sown	GR	GI	No. of grains sown	GR	GI	No. of grains sown	GR	GI	No. of grains sown	GR	GI	No. of grains sown	GR	GI	No. of grains sown	GR	GI
Wild emmer wheat																											
W01	10	0.50	0.45	10	1.00	1.00	9	1.00	1.00	50	0.54	0.54	50	1.00	1.00	16	0.94	0.94	25	0.12	0.11	25	1.00	1.00	12	1.00	0.99
W02	10	0.10	0.09	10	1.00	1.00	10	1.00	0.97	50	0.10	0.10	50	1.00	1.00	21	0.86	0.84	22	0.59	0.53	22	1.00	1.00	25	0.80	0.79
W03	10	0.80	0.76	10	1.00	1.00	10	1.00	1.00	50	0.16	0.14	50	1.00	1.00	-	-	-	25	0.68	0.63	25	1.00	1.00	25	1.00	1.00
W04	10	0.60	0.54	10	1.00	1.00	10	1.00	1.00	50	0.22	0.20	50	1.00	1.00	11	1.00	1.00	45	0.67	0.64	50	1.00	1.00	16	1.00	0.98
W05	10	0.10	0.09	10	1.00	1.00	10	1.00	0.99	50	0.16	0.16	50	1.00	1.00	25	1.00	1.00	20	0.35	0.31	20	1.00	1.00	25	1.00	0.99
W06	10	0.20	0.18	10	1.00	1.00	10	1.00	0.97	50	0.22	0.19	50	1.00	1.00	-	-	-	50	0.32	0.29	50	1.00	1.00	-	-	-
W07	10	1.00	0.94	7	1.00	1.00	10	1.00	1.00	49	0.61	0.59	50	1.00	1.00	-	-	-	16	0.81	0.81	16	0.94	0.94	25	1.00	1.00
W08	10	0.50	0.40	10	1.00	1.00	6	1.00	1.00	50	0.38	0.33	49	1.00	1.00	-	-	-	36	0.33	0.32	50	1.00	1.00	18	0.78	0.77
W09	10	0.60	0.57	9	1.00	1.00	10	1.00	0.98	57	0.67	0.63	57	1.00	1.00	21	1.00	1.00	50	0.74	0.72	36	1.00	1.00	34	1.00	1.00
W10	10	1.00	0.90	10	1.00	1.00	10	1.00	1.00	50	0.38	0.36	50	1.00	1.00	-	-	-	25	0.68	0.66	25	1.00	1.00	25	1.00	1.00
W11	9	0.78	0.66	10	1.00	1.00	10	0.80	0.80	50	0.24	0.20	50	1.00	1.00	-	-	-	50	0.64	0.63	50	1.00	1.00	25	0.92	0.92
W12	9	0.56	0.43	10	1.00	1.00	10	1.00	0.97	50	0.40	0.31	49	1.00	1.00	-	-	-	25	0.44	0.43	25	1.00	1.00	25	0.92	0.90
W13	10	0.20	0.18	10	1.00	1.00	10	1.00	0.97	50	0.28	0.25	49	1.00	1.00	10	1.00	1.00	50	0.50	0.48	50	1.00	1.00	-	-	-
W14	9	0.44	0.39	9	1.00	1.00	10	0.90	0.89	50	0.38	0.33	50	1.00	1.00	15	1.00	0.98	50	0.48	0.46	50	1.00	1.00	12	0.83	0.83
W15	10	0.80	0.70	10	1.00	1.00	10	1.00	1.00	50	0.24	0.23	50	1.00	1.00	17	1.00	1.00	25	0.24	0.21	25	1.00	1.00	25	1.00	1.00
W16	10	0.70	0.63	10	1.00	1.00	10	1.00	0.95	25	0.28	0.26	25	1.00	1.00	24	0.96	0.95	50	0.50	0.48	50	1.00	1.00	13	0.92	0.91
W17	10	0.40	0.33	10	1.00	1.00	9	0.89	0.84	50	0.10	0.08	50	1.00	1.00	-	-	-	48	0.27	0.25	50	1.00	1.00	22	0.55	0.54
W18	10	0.20	0.17	10	1.00	1.00	10	1.00	0.97	50	0.00	0.00	50	1.00	1.00	-	-	-	50	0.12	0.09	50	1.00	1.00	12	0.58	0.53
W19	10	0.00	0.00	10	1.00	1.00	10	0.70	0.68	50	0.02	0.01	50	1.00	1.00	-	-	-	50	0.14	0.13	50	1.00	1.00	18	0.44	0.39
W20	10	0.30	0.17	10	1.00	0.98	10	1.00	0.97	50	0.06	0.04	50	1.00	1.00	-	-	-	50	0.00	0.00	50	1.00	1.00	19	0.89	0.89
W21	10	0.20	0.10	10	1.00	1.00	10	0.90	0.78	50	0.00	0.00	50	1.00	1.00	11	0.82	0.82	50	0.04	0.04	50	1.00	1.00	25	0.56	0.54
W22	10	0.40	0.32	10	1.00	1.00	10	1.00	0.97	50	0.00	0.00	50	1.00	1.00	11	0.82	0.82	25	0.12	0.12	25	1.00	1.00	24	0.92	0.90
W23	10	0.90	0.81	10	1.00	1.00	10	1.00	1.00	50	0.18	0.17	50	1.00	1.00	11	0.82	0.82	50	0.24	0.23	50	0.98	0.98	20	1.00	1.00
W24	10	0.30	0.28	10	1.00	1.00	10	0.90	0.90	-	-	-	-	-	-	-	-	-	-	-	-	-	-	-	-	-	-
W25	10	0.60	0.54	10	1.00	1.00	10	1.00	1.00	50	0.08	0.07	50	1.00	1.00	13	0.92	0.92	50	0.24	0.24	50	1.00	1.00	21	0.95	0.94
W26	9	0.33	0.30	10	1.00	1.00	10	1.00	1.00	50	0.30	0.26	50	1.00	1.00	10	1.00	1.00	50	0.20	0.18	50	1.00	1.00	25	0.88	0.88
W27	10	0.40	0.39	10	1.00	1.00	7	0.86	0.83	50	0.16	0.15	50	1.00	1.00	11	1.00	1.00	50	0.26	0.25	49	1.00	1.00	21	0.67	0.61
W28	10	0.10	0.09	10	1.00	1.00	10	1.00	1.00	-	-	-	-	-	-	-	-	-	-	-	-	-	-	-	-	-	-
W29	10	0.50	0.49	9	1.00	1.00	10	1.00	0.99	-	-	-	-	-	-	-	-	-	-	-	-	-	-	-	-	-	-
W30	10	0.50	0.37	10	1.00	0.99	10	1.00	1.00	50	0.18	0.17	50	1.00	1.00	15	0.93	0.88	25	0.28	0.25	25	1.00	1.00	23	1.00	1.00
W31	10	0.20	0.18	10	1.00	1.00	10	0.90	0.90	50	0.08	0.06	50	1.00	1.00	17	1.00	1.00	25	0.08	0.04	25	1.00	1.00	25	0.88	0.88
W32	-	-	-	10	1.00	1.00	9	0.89	0.89	50	0.38	0.34	50	1.00	1.00	13	0.92	0.92	25	0.12	0.10	25	1.00	1.00	25	0.92	0.92
W33	7	1.00	0.90	6	1.00	1.00	8	1.00	0.95	49	0.27	0.26	50	1.00	1.00	16	1.00	1.00	50	0.14	0.13	50	1.00	1.00	24	1.00	0.99
W34	10	0.50	0.39	10	1.00	1.00	10	1.00	0.94	-	-	-	-	-	-	-	-	-	-	-	-	-	-	-	-	-	-
W35	9	0.22	0.21	9	1.00	1.00	10	1.00	0.98	-	-	-	-	-	-	-	-	-	-	-	-	-	-	-	-	-	-
W36	7	0.86	0.79	-	-	-	10	1.00	1.00	-	-	-	-	-	-	-	-	-	-	-	-	-	-	-	-	-	-
W37	10	0.60	0.59	10	1.00	1.00	10	1.00	1.00	50	0.14	0.14	50	1.00	1.00	20	1.00	1.00	25	0.24	0.22	25	1.00	1.00	25	1.00	1.00
W38	9	0.56	0.52	9	1.00	1.00	10	1.00	1.00	-	-	-	-	-	-	-	-	-	-	-	-	-	-	-	-	-	-
W39	7	0.86	0.80	9	1.00	1.00	9	1.00	0.93	48	0.06	0.06	50	1.00	1.00	15	0.67	0.67	25	0.16	0.16	25	1.00	1.00	14	0.79	0.78
W40	10	0.60	0.54	9	1.00	0.99	10	0.90	0.86	50	0.06	0.05	50	0.98	0.98	-	-	-	23	0.26	0.25	22	1.00	1.00	25	0.88	0.88
W41	10	0.80	0.76	10	1.00	1.00	10	1.00	1.00	50	0.14	0.13	50	1.00	1.00	11	1.00	0.99	25	0.12	0.10	25	1.00	1.00	21	0.43	0.43
W42	9	0.22	0.21	10	1.00	1.00	10	1.00	0.99	50	0.00	0.00	49	1.00	1.00	25	0.84	0.84	25	0.00	0.00	25	1.00	1.00	25	0.76	0.73
W43	-	-	-	-	-	-	-	-	-	60	0.05	0.05	60	1.00	1.00	30	1.00	1.00	24	0.08	0.08	24	0.92	0.92	63	0.98	0.98
W44	10	1.00	1.00	10	1.00	1.00	10	1.00	1.00	50	0.30	0.28	50	1.00	1.00	-	-	-	25	0.32	0.32	25	1.00	1.00	25	0.88	0.88
W45	10	0.70	0.70	10	1.00	1.00	10	1.00	1.00	50	0.04	0.04	50	1.00	1.00	18	1.00	1.00	18	0.06	0.06	18	1.00	1.00	25	1.00	1.00
W46	10	0.20	0.20	9	1.00	1.00	10	1.00	1.00	24	0.63	0.62	24	1.00	1.00	11	1.00	1.00	24	0.04	0.03	24	1.00	1.00	-	-	-
W47	9	1.00	0.94	10	1.00	0.96	10	1.00	0.98	25	0.24	0.23	25	1.00	1.00	10	1.00	1.00	25	0.04	0.04	25	1.00	1.00	10	0.70	0.70
W48	10	1.00	1.00	10	1.00	0.99	10	1.00	0.99	50	0.84	0.83	50	0.98	0.98	16	1.00	1.00	50	0.06	0.06	50	1.00	1.00			
W49	10	0.30	0.28	10	1.00	1.00	8	1.00	0.94	50	0.04	0.04	50	1.00	0.96	-	-	-	25	0.28	0.28	25	1.00	1.00	10	0.80	0.78
W50	9	0.67	0.59	10	1.00	0.99	10	0.80	0.71	-	-	-	-	-	-	-	-	-	-	-	-	-	-	-	-	-	-
W51	9	0.78	0.70	10	1.00	0.98	10	0.90	0.84	50	0.08	0.06	50	1.00	0.99	-	-	-	17	0.06	0.06	17	1.00	1.00	19	1.00	1.00
W52	10	0.30	0.22	9	1.00	0.98	-	-	-	50	0.06	0.06	48	1.00	0.99	13	0.31	0.31	25	0.20	0.20	25	1.00	1.00	19	0.89	0.89
W53	10	0.20	0.19	10	1.00	1.00	10	0.90	0.85	50	0.30	0.28	50	1.00	1.00	-	-	-	25	0.12	0.12	25	1.00	1.00	-	-	-
W54	9	0.67	0.62	10	1.00	1.00	9	1.00	0.91	50	0.04	0.03	50	1.00	1.00	-	-	-	25	0.08	0.06	25	1.00	1.00	16	1.00	1.00
W55	9	0.78	0.70	10	1.00	1.00	10	1.00	0.96	50	0.42	0.39	50	1.00	1.00	-	-	-	50	0.32	0.31	50	1.00	1.00	-	-	-
W56	9	0.44	0.38	7	1.00	0.97	5	1.00	0.94	50	0.28	0.27	50	1.00	1.00	-	-	-	50	0.46	0.45	50	1.00	1.00	-	-	-
W57	10	0.70	0.64	8	1.00	1.00	10	1.00	1.00	50	0.46	0.45	49	1.00	1.00	-	-	-	-	-	-	-	-	-	-	-	-
W58	10	1.00	0.98	10	1.00	1.00	10	1.00	1.00	25	0.76	0.76	25	1.00	1.00	20	1.00	1.00	-	-	-	-	-	-	17	1.00	1.00
W59	8	0.50	0.45	10	1.00	0.98	10	1.00	0.96	50	0.22	0.22	50	1.00	1.00	10	1.00	1.00	-	-	-	-	-	-	-	-	-
W60	10	0.60	0.54	10	1.00	1.00	10	1.00	0.99	50	0.02	0.02	50	1.00	1.00	10	0.90	0.90	25	0.12	0.12	25	1.00	1.00	22	1.00	1.00
W61	8	0.00	0.00	10	1.00	1.00	10	1.00	0.99	50	0.00	0.00	51	0.98	0.98	-	-	-	-	-	-	-	-	-	-	-	-
W62	10	0.80	0.79	10	1.00	0.98	10	1.00	1.00	51	0.29	0.29	50	1.00	1.00	13	1.00	1.00	-	-	-	-	-	-	18	1.00	1.00
W63	10	0.80	0.67	10	1.00	0.99	10	1.00	0.98	50	0.30	0.23	50	1.00	1.00	-	-	-	25	0.52	0.52	25	1.00	1.00	12	1.00	1.00
W64	10	0.70	0.61	10	1.00	1.00	10	1.00	0.98	50	0.12	0.10	50	1.00	1.00	14	1.00	1.00	25	0.28	0.25	25	1.00	1.00	19	1.00	1.00
W65	6	0.83	0.72	10	1.00	1.00	10	1.00	0.99	49	0.10	0.09	49	1.00	1.00	11	0.91	0.90	14	0.07	0.07	14	0.93	0.93	17	0.71	0.65
W66	10	0.90	0.73	10	1.00	1.00	10	1.00	1.00	50	0.20	0.17	49	1.00	1.00	-	-	-	50	0.36	0.36	50	1.00	1.00	-	-	-
W67	6	0.33	0.32	10	1.00	1.00	8	1.00	0.96	50	0.08	0.07	50	1.00	1.00	-	-	-	25	0.08	0.05	25	1.00	1.00	-	-	-

(to be continued)

Supplemental Table 4. (continued)

Accession No.	2009																		2010									2014 (sown on September 9)																	
	Sown on September 6									Sown on November 6									Sown on September 19									Grown at field									Grown at greenhouse								
	Two-grained spikelets						One-grained spikelets			Two-grained spikelets						One-grained spikelets			Two-grained spikelets						One-grained spikelets			Two-grained spikelets						One-grained spikelets			Two-grained spikelets						One-grained spikelets		
	First florets			Second florets			First florets			First florets			Second florets			First florets			First florets			Second florets			First florets			First florets			Second florets			First florets			First florets			Second florets			First florets		
	No. of grains sown	GR	GI	No. of grains sown	GR	GI	No. of grains sown	GR	GI	No. of grains sown	GR	GI	No. of grains sown	GR	GI	No. of grains sown	GR	GI	No. of grains sown	GRs	GIs	No. of grains sown	GRs	GIs	No. of grains sown	GRs	GIs	No. of grains sown	GR	GI	No. of grains sown	GR	GI	No. of grains sown	GR	GI	No. of grains sown	GR	GI	No. of grains sown	GR	GI	No. of grains sown	GR	GI
Domesticated emmer wheat																																													
D01	10	1.00	0.98	10	1.00	0.99	10	1.00	1.00	10	1.00	1.00	10	1.00	1.00	10	1.00	1.00	-	-	-	-	-	-	-	-	-	50	1.00	1.00	50	1.00	1.00	14	1.00	1.00	50	1.00	1.00	50	1.00	1.00	11	1.00	1.00
D02	10	1.00	1.00	10	1.00	1.00	10	1.00	1.00	10	1.00	1.00	10	1.00	1.00	7	1.00	1.00	10	1.00	1.00	10	1.00	1.00	10	1.00	1.00	50	1.00	1.00	50	1.00	1.00	-	-	-	50	1.00	1.00	50	1.00	1.00	-	-	-
D03	10	1.00	1.00	10	1.00	1.00	9	1.00	1.00	10	1.00	1.00	10	1.00	1.00	-	-	-	10	1.00	1.00	10	1.00	1.00	9	1.00	1.00	50	1.00	1.00	50	1.00	1.00	-	-	-	50	1.00	1.00	50	1.00	1.00	-	-	-
D04	-	-	-	-	-	-	-	-	-	-	-	-	-	-	-	-	-	-	10	1.00	1.00	10	1.00	1.00	10	1.00	0.98	-	-	-	-	-	-	-	-	-	-	-	-	-	-	-	-	-	-
D05	10	1.00	1.00	10	1.00	0.98	6	1.00	1.00	10	1.00	1.00	10	1.00	1.00	-	-	-	10	1.00	1.00	10	1.00	1.00	10	1.00	1.00	50	1.00	1.00	50	1.00	1.00	12	1.00	1.00	25	1.00	1.00	25	1.00	1.00	50	1.00	1.00
D06	10	1.00	1.00	10	1.00	1.00	8	1.00	1.00	10	1.00	1.00	10	1.00	1.00	10	1.00	1.00	10	1.00	1.00	10	1.00	1.00	10	1.00	1.00	50	1.00	1.00	50	1.00	1.00	25	1.00	1.00	50	1.00	1.00	50	1.00	1.00	50	1.00	1.00
D07	10	1.00	1.00	10	1.00	1.00	-	-	-	10	1.00	1.00	10	1.00	1.00	-	-	-	10	1.00	1.00	10	1.00	1.00	10	1.00	1.00	49	1.00	1.00	49	1.00	1.00	13	1.00	1.00	50	1.00	1.00	50	1.00	1.00	25	1.00	1.00
D08	10	1.00	1.00	10	1.00	1.00	7	1.00	1.00	10	1.00	1.00	10	1.00	1.00	-	-	-	10	1.00	1.00	10	1.00	0.98	10	1.00	1.00	50	1.00	1.00	50	1.00	1.00	23	1.00	1.00	25	1.00	1.00	25	1.00	1.00	50	1.00	1.00
D09	10	1.00	1.00	10	1.00	1.00	10	1.00	0.99	10	1.00	1.00	10	1.00	1.00	10	1.00	1.00	-	-	-	-	-	-	-	-	-	50	0.98	0.98	50	1.00	1.00	50	1.00	1.00	50	1.00	1.00	50	1.00	1.00	25	1.00	1.00
D10	10	1.00	1.00	10	1.00	1.00	10	1.00	1.00	10	1.00	1.00	10	1.00	1.00	-	-	-	10	1.00	1.00	10	1.00	1.00	10	1.00	1.00	50	1.00	1.00	50	1.00	1.00	-	-	-	50	1.00	1.00	50	1.00	1.00	14	1.00	1.00
D11	10	1.00	1.00	10	1.00	1.00	10	1.00	1.00	10	1.00	1.00	10	1.00	1.00	6	1.00	1.00	-	-	-	-	-	-	-	-	-	50	1.00	1.00	50	1.00	1.00	-	-	-	50	1.00	1.00	50	1.00	1.00	25	1.00	1.00
D12	10	1.00	1.00	10	1.00	1.00	10	1.00	1.00	10	1.00	1.00	10	1.00	1.00	10	1.00	1.00	-	-	-	-	-	-	-	-	-	25	1.00	1.00	25	1.00	1.00	13	1.00	1.00	50	1.00	1.00	50	1.00	1.00	50	1.00	1.00
D13	10	1.00	1.00	10	1.00	1.00	10	1.00	1.00	10	1.00	1.00	10	1.00	1.00	-	-	-	10	1.00	1.00	10	1.00	1.00	10	1.00	1.00	50	1.00	1.00	50	1.00	1.00	10	1.00	1.00	50	1.00	1.00	50	1.00	1.00	25	1.00	1.00
D14	10	1.00	1.00	10	1.00	1.00	10	1.00	1.00	10	1.00	1.00	10	1.00	1.00	10	1.00	1.00	-	-	-	-	-	-	-	-	-	50	1.00	1.00	50	1.00	1.00	-	-	-	50	1.00	1.00	50	1.00	1.00	25	1.00	1.00
D15	10	1.00	1.00	10	1.00	1.00	10	1.00	1.00	10	1.00	1.00	10	1.00	1.00	10	1.00	1.00	10	1.00	1.00	10	1.00	1.00	10	1.00	1.00	50	1.00	1.00	50	1.00	1.00	18	1.00	1.00	50	1.00	1.00	50	1.00	1.00	25	1.00	1.00
D16	10	1.00	1.00	10	1.00	1.00	-	-	-	10	1.00	1.00	10	1.00	1.00	10	1.00	1.00	-	-	-	-	-	-	-	-	-	50	1.00	1.00	50	1.00	1.00	-	-	-	25	1.00	1.00	25	1.00	1.00	25	1.00	1.00
D17	10	1.00	1.00	10	1.00	1.00	10	1.00	1.00	10	1.00	1.00	10	1.00	1.00	7	1.00	1.00	-	-	-	-	-	-	-	-	-	-	-	-	-	-	-	-	-	-	-	-	-	-	-	-	-	-	-
D18	-	-	-	-	-	-	-	-	-	-	-	-	-	-	-	-	-	-	10	1.00	1.00	10	1.00	1.00	7	1.00	1.00	50	1.00	1.00	50	1.00	1.00	-	-	-	50	1.00	1.00	50	1.00	1.00	13	1.00	1.00
D19	10	1.00	1.00	10	1.00	1.00	10	1.00	1.00	10	1.00	1.00	10	1.00	1.00	10	1.00	1.00	10	1.00	1.00	10	1.00	1.00	10	1.00	1.00	50	1.00	1.00	50	1.00	1.00	19	1.00	1.00	-	-	-	-	-	-	-	-	-
D20	10	1.00	1.00	10	1.00	1.00	5	1.00	1.00	10	1.00	1.00	10	1.00	1.00	-	-	-	10	1.00	1.00	10	1.00	1.00	10	1.00	1.00	-	-	-	-	-	-	-	-	-	15	1.00	1.00	14	1.00	1.00	72	1.00	1.00
D21	10	1.00	1.00	10	1.00	1.00	6	1.00	1.00	10	1.00	1.00	10	1.00	1.00	-	-	-	10	1.00	1.00	10	1.00	1.00	10	1.00	1.00	50	1.00	1.00	50	1.00	1.00	-	-	-	50	1.00	1.00	50	1.00	1.00	14	1.00	1.00
D22	-	-	-	-	-	-	-	-	-	-	-	-	-	-	-	-	-	-	10	1.00	1.00	10	1.00	1.00	10	1.00	1.00	-	-	-	-	-	-	-	-	-	-	-	-	-	-	-	-	-	-
D23	10	1.00	1.00	10	1.00	1.00	10	1.00	1.00	10	1.00	1.00	10	1.00	1.00	-	-	-	10	1.00	1.00	10	1.00	1.00	10	1.00	1.00	90	1.00	1.00	93	1.00	1.00	21	1.00	1.00	83	1.00	1.00	83	1.00	1.00	10	1.00	1.00
D24	-	-	-	-	-	-	-	-	-	-	-	-	-	-	-	-	-	-	10	1.00	1.00	10	1.00	1.00	10	1.00	1.00	-	-	-	-	-	-	-	-	-	-	-	-	-	-	-	-	-	-
D25	-	-	-	-	-	-	-	-	-	-	-	-	-	-	-	-	-	-	10	1.00	1.00	10	1.00	1.00	10	1.00	1.00	-	-	-	-	-	-	-	-	-	-	-	-	-	-	-	-	-	-
D26	-	-	-	-	-	-	-	-	-	-	-	-	-	-	-	-	-	-	10	1.00	1.00	10	1.00	1.00	10	1.00	1.00	-	-	-	-	-	-	-	-	-	-	-	-	-	-	-	-	-	-
D27	-	-	-	-	-	-	-	-	-	-	-	-	-	-	-	-	-	-	10	1.00	1.00	10	1.00	1.00	10	1.00	1.00	-	-	-	-	-	-	-	-	-	-	-	-	-	-	-	-	-	-
D28	-	-	-	-	-	-	-	-	-	-	-	-	-	-	-	-	-	-	10	1.00	1.00	10	1.00	1.00	10	1.00	1.00	-	-	-	-	-	-	-	-	-	-	-	-	-	-	-	-	-	-
D29	-	-	-	-	-	-	-	-	-	-	-	-	-	-	-	-	-	-	10	1.00	1.00	10	1.00	1.00	10	1.00	1.00	-	-	-	-	-	-	-	-	-	-	-	-	-	-	-	-	-	-
D30	-	-	-	-	-	-	-	-	-	-	-	-	-	-	-	-	-	-	10	1.00	1.00	10	1.00	1.00	10	1.00	1.00	-	-	-	-	-	-	-	-	-	-	-	-	-	-	-	-	-	-
D31	-	-	-	-	-	-	-	-	-	-	-	-	-	-	-	-	-	-	10	1.00	1.00	10	1.00	1.00	10	1.00	1.00	-	-	-	-	-	-	-	-	-	-	-	-	-	-	-	-	-	-
D32	-	-	-	-	-	-	-	-	-	-	-	-	-	-	-	-	-	-	10	1.00	1.00	10	1.00	1.00	10	1.00	1.00	-	-	-	-	-	-	-	-	-	-	-	-	-	-	-	-	-	-
D33	-	-	-	-	-	-	-	-	-	-	-	-	-	-	-	-	-	-	10	1.00	1.00	10	1.00	1.00	10	1.00	1.00	-	-	-	-	-	-	-	-	-	-	-	-	-	-	-	-	-	-
D34	-	-	-	-	-	-	-	-	-	-	-	-	-	-	-	-	-	-	10	1.00	1.00	10	1.00	1.00	10	1.00	1.00	-	-	-	-	-	-	-	-	-	-	-	-	-	-	-	-	-	-
D35	-	-	-	-	-	-	-	-	-	-	-	-	-	-	-	-	-	-	10	1.00	1.00	10	1.00	1.00	10	1.00	1.00	-	-	-	-	-	-	-	-	-	-	-	-	-	-	-	-	-	-
D36	-	-	-	-	-	-	-	-	-	-	-	-	-	-	-	-	-	-	10	1.00	1.00	10	1.00	1.00	10	1.00	1.00	-	-	-	-	-	-	-	-	-	-	-	-	-	-	-	-	-	-
D37	-	-	-	-	-	-	-	-	-	-	-	-	-	-	-	-	-	-	10	1.00	1.00	10	1.00	1.00	10	1.00	1.00	-	-	-	-	-	-	-	-	-	-	-	-	-	-	-	-	-	-
D38	10	1.00	1.00	10	1.00	1.00	10	1.00	1.00	10	1.00	1.00	10	1.00	1.00	6	1.00	1.00	-	-	-	-	-	-	-	-	-	50	1.00	1.00	50	1.00	1.00	19	1.00	1.00	50	1.00	1.00	50	1.00	1.00	14	1.00	1.00
D39	-	-	-	-	-	-	-	-	-	-	-	-	-	-	-	-	-	-	10	1.00	1.00	10	1.00	1.00	10	1.00	1.00	-	-	-	-	-	-	-	-	-	-	-	-	-	-	-	-	-	-
D40	-	-	-	-	-	-	-	-	-	-	-	-	-	-	-	-	-	-	10	1.00	1.00	10	1.00	1.00	10	1.00	1.00	-	-	-	-	-	-	-	-	-	-	-	-	-	-	-	-	-	-
D41	-	-	-	-	-	-	-	-	-	-	-	-	-	-	-	-	-	-	10	1.00	1.00	10	1.00	1.00	10	1.00	1.00	-	-	-	-	-	-	-	-	-	-	-	-	-	-	-	-	-	-
D42	-	-	-	-	-	-	-	-	-	-	-	-	-	-	-	-	-	-	10	1.00	1.00	10	1.00	1.00	10	1.00	1.00	-	-	-	-	-	-	-	-	-	-	-	-	-	-	-	-	-	-
D43	-	-	-	-	-	-	-	-	-	-	-	-	-	-	-	-	-	-	10	1.00	1.00	10	1.00	1.00	10	1.00	1.00	-	-	-	-	-	-	-	-	-	-	-	-	-	-	-	-	-	-
D44	-	-	-	-	-	-	-	-	-	-	-	-	-	-	-	-	-	-	10	1.00	1.00	10	1.00	1.00	10	1.00	1.00	-	-	-	-	-	-	-	-	-	-	-	-	-	-	-	-	-	-
D45	-	-	-	-	-	-	-	-	-	-	-	-	-	-	-	-	-	-	10	1.00	1.00	10	1.00	1.00	10	1.00	1.00	-	-	-	-	-	-	-	-	-	-	-	-	-	-	-	-	-	-
D46	-	-	-	-	-	-	-	-	-	-	-	-	-	-	-	-	-	-	10	1.00	1.00	10	1.00	1.00	10	1.00	1.00	-	-	-	-	-	-	-	-	-	-	-	-	-	-	-	-	-	-
D47	-	-	-	-	-	-	-	-	-	-	-	-	-	-	-	-	-	-	10	1.00	1.00	10	1.00	1.00	10	1.00	1.00	-	-	-	-	-	-	-	-	-	-	-	-	-	-	-	-	-	-
D48	-	-	-	-	-	-	-	-	-	-	-	-	-	-	-	-	-	-	10	1.00	1.00	10	1.00	1.00	10	1.00	1.00	-	-	-	-	-	-	-	-	-	-	-	-	-	-	-	-	-	-
D49	-	-	-	-	-	-	-	-	-	-	-	-	-	-	-	-	-	-	10	1.00	1.00	10	1.00	1.00	10	1.00	1.00	-	-	-	-	-	-	-	-	-	-	-	-	-	-	-	-	-	-
D50	-	-	-	-	-	-	-	-	-	-	-	-	-	-	-	-	-	-	10	1.00	1.00	10	1.00	1.00	10	1.00	1.00	-	-	-	-	-	-	-	-	-	-	-	-	-	-	-	-	-	-
D51	-	-	-	-	-	-	-	-	-	-	-	-	-	-	-	-	-	-	10	1.00	1.00	10	1.00	1.00	10	1.00	1.00	-	-	-	-	-	-	-	-	-	-	-	-	-	-	-	-	-	-
D52	-	-	-	-	-	-	-	-	-	-	-	-	-	-	-	-	-	-	10	1.00	1.00	10	1.00	1.00	10	1.00	1.00	-	-	-	-	-	-	-	-	-	-	-	-	-	-	-	-	-	-
D53	-	-	-	-	-	-	-	-	-	-	-	-	-	-	-	-	-	-	10	1.00	1.00	10	1.00	1.00	10	1.00	1.00	-	-	-	-	-	-	-	-	-	-	-	-	-	-	-	-	-	-
D54	-	-	-	-	-	-	-	-	-	-	-	-	-	-	-	-	-	-	10	1.00	1.00	10	1.00	1.00	10	1.00	1.00	-	-	-	-	-	-	-	-	-	-	-	-	-	-	-	-	-	-
D55	-	-	-	-	-	-	-	-	-	-	-	-	-	-	-	-	-	-	10	1.00	1.00	10	1.00	1.00	10	1.00	1.00	-	-	-	-	-	-	-	-	-	-	-	-	-	-	-	-	-	-
D56	-	-	-	-	-	-	-	-	-	-	-	-	-	-	-	-	-	-	10	1.00	1.00	10	1.00	1.00	10	1.00	1.00	-	-	-	-	-	-	-	-	-	-	-	-	-	-	-	-	-	-
D57	-	-	-	-	-	-	-	-	-	-	-	-	-	-	-	-	-	-	10	1.00	1.00	10	1.00	1.00	10	1.00	1.00	-	-	-	-	-	-	-	-	-	-	-	-	-	-	-	-	-	-
D58	-	-	-	-	-	-	-	-	-	-	-	-	-	-	-	-	-	-	10	1.00	1.00	10	1.00	1.00	10	1.00	1.00	-	-	-	-	-	-	-	-	-	-	-	-	-	-	-	-	-	-
D59	-	-	-	-	-	-	-	-	-	-	-	-	-	-	-	-	-	-	10	1.00	1.00	10	1.00	1.00	10	1.00	1.00	-	-	-	-	-	-	-	-	-	-	-	-	-	-	-	-	-	-
D60	-	-	-	-	-	-	-	-	-	-	-	-	-	-	-	-	-	-	10	1.00	1.00	10	1.00	1.00	10	1.00	1.00	-	-	-	-	-	-	-	-	-	-	-	-	-	-	-	-	-	-
D61	-	-	-	-	-	-	-	-	-	-	-	-	-	-	-	-	-	-	10	1.00	1.00	10	1.00	1.00	10	1.00	1.00	-	-	-	-	-	-	-	-	-	-	-	-	-	-	-	-	-	-
D62	10	1.00	1.00	10	1.00	1.00	10	1.00	1.00	10	1.00	1.00	10	1.00	1.00	5	1.00	1.00	10	1.00	1.00	10	1.00	1.00	10	1.00	1.00	50	1.00	1.00	50	1.00	1.00	15	1.00	1.00	50	1.00	1.00	50	1.00	1.00	25	1.00	1.00
D63	10	1.00	1.00	10	1.00	1.00	10	1.00	1.00	10	1.00	1.00	10	1.00	1.00	10	1.00	1.00	-	-	-	-	-	-	-	-	-	50	1.00	1.00	50	1.00	1.00	15	1.00	1.00	25	1.00	1.00	25	1.00	1.00	25	1.00	1.00
D64	10	1.00	1.00	10	1.00	1.00	10	1.00	1.00	10	1.00	1.00	10	1.00	1.00	8	1.00	1.00	-	-	-	-	-	-	-	-	-	50	1.00	1.00	50	1.00	1.00	23	1.00	1.00	50	1.00	1.00	50	1.00	1.00	25	1.00	1.00
D65	10	1.00	1.00	10	1.00	1.00	10	1.00	1.00	10	1.00	1.00	10	1.00	1.00	10	1.00	1.00	-	-	-	-	-	-	-	-	-	50	1.00	1.00	50	1.00	1.00	25	1.00	1.00	50	1.00	1.00	50	1.00	1.00	25	1.00	1.00

(to be continued)

Supplemental Table 4. (continued)

Accession No.	2009																	
	Sown on September 6									Sown on November 6								
	Two-grained spikelets						One-grained spikelets			Two-grained spikelets						One-grained spikelets		
	First florets			Second florets			First florets			First florets			Second florets			First florets		
	No. of grains sown	GR	GI	No. of grains sown	GR	GI	No. of grains sown	GR	GI	No. of grains sown	GR	GI	No. of grains sown	GR	GI	No. of grains sown	GR	GI
D66	10	1.00	1.00	10	1.00	1.00	10	1.00	1.00	10	1.00	1.00	10	1.00	1.00	6	1.00	1.00
D67	10	1.00	1.00	10	1.00	1.00	-	-	-	10	1.00	1.00	10	1.00	1.00	-	-	-
D68	10	1.00	1.00	10	1.00	1.00	10	1.00	1.00	10	1.00	1.00	10	1.00	1.00	10	1.00	1.00
D69	10	1.00	1.00	10	1.00	1.00	10	1.00	1.00	10	1.00	1.00	10	1.00	1.00	6	1.00	1.00
D70	-	-	-	-	-	-	-	-	-	-	-	-	-	-	-	-	-	-
D71	-	-	-	-	-	-	-	-	-	-	-	-	-	-	-	-	-	-
D72	10	1.00	1.00	10	1.00	1.00	10	1.00	1.00	10	1.00	1.00	10	1.00	1.00	-	-	-
D73	10	1.00	1.00	10	1.00	1.00	10	1.00	1.00	10	1.00	1.00	10	1.00	1.00	10	1.00	1.00
D74	-	-	-	-	-	-	-	-	-	-	-	-	-	-	-	-	-	-
D75	-	-	-	-	-	-	-	-	-	-	-	-	-	-	-	-	-	-
D76	-	-	-	-	-	-	-	-	-	-	-	-	-	-	-	-	-	-
D77	10	1.00	1.00	10	1.00	1.00	10	1.00	1.00	10	1.00	1.00	10	1.00	1.00	-	-	-
D78	10	1.00	1.00	10	1.00	1.00	10	1.00	1.00	10	1.00	1.00	10	1.00	1.00	10	1.00	1.00
D79	10	1.00	1.00	10	1.00	1.00	10	1.00	1.00	10	1.00	1.00	10	1.00	1.00	8	1.00	1.00
D80	10	1.00	1.00	10	1.00	1.00	10	1.00	1.00	10	1.00	1.00	10	1.00	1.00	10	1.00	1.00
D81	10	1.00	1.00	10	1.00	1.00	9	1.00	1.00	10	1.00	1.00	10	1.00	1.00	-	-	-
D82	10	1.00	1.00	10	1.00	1.00	10	1.00	1.00	10	1.00	1.00	10	1.00	1.00	8	1.00	1.00

Accession No.	2010									2014 (sown on September 9)																	
	Sown on September 19									Grown at field									Grown at greenhouse								
	Two-grained spikelets						One-grained spikelets			Two-grained spikelets						One-grained spikelets			Two-grained spikelets						One-grained spikelets		
	First florets			Second florets			First florets			First florets			Second florets			First florets			First florets			Second florets			First florets		
	No. of grains sown	GRs	GIs	No. of grains sown	GRs	GIs	No. of grains sown	GRs	GIs	No. of grains sown	GR	GI	No. of grains sown	GR	GI	No. of grains sown	GR	GI	No. of grains sown	GR	GI	No. of grains sown	GR	GI	No. of grains sown	GR	GI
D66	-	-	-	-	-	-	-	-	-	50	1.00	1.00	50	1.00	1.00	15	1.00	1.00	50	1.00	1.00	50	1.00	1.00	25	1.00	1.00
D67	-	-	-	-	-	-	-	-	-	50	1.00	1.00	50	1.00	1.00	50	1.00	1.00	50	1.00	1.00	50	1.00	1.00	25	1.00	1.00
D68	10	1.00	1.00	10	1.00	1.00	10	1.00	1.00	50	1.00	1.00	50	1.00	1.00	25	1.00	1.00	50	1.00	1.00	50	1.00	1.00	25	1.00	1.00
D69	10	1.00	1.00	10	1.00	0.98	10	1.00	0.97	50	0.98	0.94	50	0.98	0.95	50	0.92	0.85	50	1.00	1.00	50	1.00	1.00	25	1.00	1.00
D70	10	1.00	1.00	10	1.00	1.00	10	1.00	1.00	-	-	-	-	-	-	-	-	-	-	-	-	-	-	-	-	-	-
D71	10	1.00	1.00	10	1.00	1.00	10	1.00	1.00	-	-	-	-	-	-	-	-	-	-	-	-	-	-	-	-	-	-
D72	10	1.00	1.00	10	1.00	1.00	10	1.00	1.00	50	1.00	1.00	50	1.00	1.00	21	1.00	1.00	50	1.00	1.00	50	1.00	1.00	17	1.00	1.00
D73	10	1.00	1.00	10	1.00	1.00	10	1.00	1.00	50	1.00	1.00	50	1.00	1.00	-	-	-	50	1.00	1.00	50	1.00	1.00	11	1.00	1.00
D74	10	1.00	1.00	10	1.00	1.00	10	1.00	1.00	-	-	-	-	-	-	-	-	-	-	-	-	-	-	-	-	-	-
D75	10	1.00	1.00	10	1.00	1.00	10	1.00	1.00	-	-	-	-	-	-	-	-	-	-	-	-	-	-	-	-	-	-
D76	10	1.00	1.00	10	1.00	1.00	10	1.00	1.00	-	-	-	-	-	-	-	-	-	-	-	-	-	-	-	-	-	-
D77	10	1.00	1.00	10	1.00	1.00	10	1.00	1.00	50	1.00	1.00	50	1.00	1.00	-	-	-	50	1.00	1.00	50	1.00	1.00	-	-	-
D78	10	1.00	1.00	10	1.00	1.00	10	1.00	1.00	50	1.00	1.00	50	1.00	1.00	17	1.00	1.00	50	1.00	1.00	50	1.00	1.00	15	1.00	1.00
D79	10	1.00	1.00	10	1.00	1.00	10	1.00	1.00	50	1.00	1.00	49	1.00	1.00	-	-	-	50	1.00	1.00	50	1.00	1.00	-	-	-
D80	10	1.00	1.00	10	1.00	1.00	10	1.00	1.00	50	1.00	1.00	50	1.00	1.00	-	-	-	50	1.00	1.00	50	1.00	1.00	10	1.00	1.00
D81	10	1.00	1.00	10	1.00	1.00	10	1.00	1.00	50	1.00	1.00	50	1.00	1.00	-	-	-	50	1.00	1.00	50	1.00	1.00	-	-	-
D82	10	1.00	1.00	10	1.00	1.00	10	1.00	1.00	50	1.00	0.99	50	1.00	0.98	16	1.00	0.98	50	1.00	1.00	50	1.00	1.00	20	1.00	1.00

Supplemental Table 5. Comparison of GWR values and GI values observed in wild emmer wheat accessions in September across eight geographical regions, and the results of Tukey–Kramer test for GWR values (in the upper triangles), and Steel–Dwass test for GI values (in the lower triangles)

Year of observation / Countries (Regions) [a]	GWR No.of accessions	GWR Mean	GWR SE	GI No.of accessions	GI Mean	GI Median	GI IQR [b]	Statistical values [c]: Iran	Iraq (NE)	Iraq (N)	Israel (UJV)	Israel (JM)	Syria	Turkey (D)	Turkey (G)
2009															
Iran	3	0.83	0.04	3	0.52	0.58	0.50		1.460	0.289	1.198	0.088	1.441	0.989	0.930
Iraq (NE)	7	0.71	0.04	7	0.29	0.09	0.48	0.705		2.473	0.288	1.771	0.246	0.487	3.514
Iraq (N)	11	0.85	0.04	11	0.32	0.19	0.60	0.398	0.327		2.039	0.515	2.096	1.687	1.013
Israel (UJV)	6	0.73	0.03	6	0.28	0.35	0.39	1.050	0.294	0.205		1.413	0.467	0.206	2.991
Israel (JM)	7	0.82	0.04	7	0.29	0.07	0.49	0.941	0.000	0.327	0.147		1.617	1.129	1.425
Syria	3	0.69	0.01	3	0.19	0.00	0.28	1.159	0.608	0.891	0.270	0.365		0.623	2.800
Turkey (D)	5	0.74	0.05	5	0.29	0.29	0.37	0.764	0.169	0.174	0.187	0.084	0.476		2.551
Turkey (G)	16	0.89	0.04	16	0.37	0.26	0.50	0.560	0.941	0.645	0.370	0.840	1.123	0.622	
2010															
Iran	3	0.76	0.02	3	0.71	0.94	0.40		1.013	0.750	1.993	0.050	0.766	0.730	0.792
Iraq (NE)	8	0.69	0.03	8	0.30	0.23	0.22	1.633		0.494	1.333	1.392	0.089	2.030	0.433
Iraq (N)	16	0.72	0.02	15	0.49	0.49	0.34	1.126	1.711		1.995	1.117	0.245	1.841	0.076
Israel (UJV)	7	0.63	0.02	7	0.48	0.59	0.41	1.026	1.448	0.035		2.638	1.087	3.084	1.936
Israel (JM)	7	0.77	0.06	7	0.53	0.54	0.26	0.798	1.507	0.423	0.192		0.957	0.834	1.176
Syria	3	0.70	0.02	3	0.67	0.67	0.06	0.655	1.837	1.245	1.026	1.026		1.549	0.202
Turkey (D)	4	0.82	0.11	4	0.59	0.58	0.38	0.178	1.529	0.550	0.378	0.380	0.354		1.889
Turkey (G)	16	0.71	0.01	16	0.49	0.49	0.37	1.343	1.685	0.020	0.167	0.301	1.231	0.756	
2014 (Field)															
Iran	3	0.75	0.06	3	0.56	0.62	0.30		2.310	3.175	3.637	1.803	2.591	1.871	1.754
Iraq (NE)	7	0.67	0.01	7	0.04	0.01	0.05	2.423		1.007	1.757	0.654	0.756	0.263	1.059
Iraq (N)	10	0.64	0.02	10	0.16	0.14	0.16	2.028	2.544		0.932	1.716	0.039	1.117	2.402
Israel (UJV)	6	0.61	0.02	6	0.13	0.06	0.18	1.807	1.654	0.976		2.385	0.644	1.770	3.027
Israel (JM)	7	0.68	0.03	7	0.30	0.27	0.30	1.254	2.067	1.073	0.714		1.263	0.295	0.295
Syria	3	0.64	0.01	3	0.17	0.17	0.06	1.771	2.083	0.169	0.775	0.798		0.899	1.592
Turkey (D)	4	0.67	0.03	5	0.18	0.05	0.24	1.640	1.323	0.612	0.000	0.488	0.447		0.568
Turkey (G)	15	0.69	0.01	16	0.27	0.24	0.15	1.565	3.478	1.924	2.064	0.334	1.230	1.321	
2014 (Greenhouse)															
Iran	3	0.58	0.00	3	0.04	0.04	0.02		1.909	0.611	0.747	2.178	2.279	2.323	2.452
Iraq (NE)	7	0.67	0.02	7	0.12	0.12	0.12	1.372		1.856	1.318	0.668	0.788	0.648	0.511
Iraq (N)	10	0.61	0.02	10	0.17	0.17	0.13	2.373	1.320		0.261	2.090	2.215	2.363	2.814
Israel (UJV)	5	0.62	0.03	5	0.14	0.12	0.14	1.800	0.406	0.491		1.688	1.801	1.821	1.948
Israel (JM)	3	0.69	0.05	3	0.30	0.31	0.17	1.964	1.709	1.356	1.500		0.101	0.112	0.359
Syria	3	0.70	0.05	3	0.37	0.36	0.13	1.964	2.393	2.373	1.938	0.655		0.225	0.490
Turkey (D)	5	0.69	0.04	5	0.11	0.08	0.06	1.043	0.488	1.227	0.731	1.640	1.938		0.282
Turkey (G)	15	0.68	0.01	16	0.49	0.48	0.31	2.684	3.609	3.849	3.139	1.566	0.895	2.974	

[a] Iraq (NE): Iraq (Northeastern), Iraq (N): Iraq (Northern), Israel (UJV): Israel (Upper Jordan Valley and Mt. Hermon), Israel (JM): Israel (Judean Mountains and Foothills), Turkey (D): Turkey (Karacadağ region near Diyarbakir), and Turkey (G): Turkey (Karadağ region near Gaziantep), respectively. For the detailed collection localities, see Supplemental Table 1; [b] IQR: interquartile range; [c] Orange and red figures indicate significantly different at $p = 0.05$, and at $p = 0.01$, respectively

Supplemental Table 6. One-grain weights, and GWR, GR, and GI values in wild and domesticated parental lines, F_1 grains, reciprocal F_1 hybrids, and F_2 populations derived from self-pollination of the F_1 hybrids (sown on September 7, 2012)

		Two-grained spikelets											One-grained spikelets	
		First florets					Second florets						First florets	
Line and Population No.	Plant No.	No. of grains measured	One-grain weight (mg)	No. of grains sown	GR	GI	No. of grains measured	One-grain weight (mg)	No. of grains sown	GR	GI	GWR	No. of grains measured	One-grain weight (mg)
Wild emmer parent														
W43	1	25	13.81	not observed			25	22.82	not observed			0.608	66	22.83
	2	37	12.77				37	19.97				0.645	54	18.82
	3	51	13.99	49	0.14	0.12	51	21.03	49	1.00	1.00	0.669	36	20.59
	4	31	12.68	not observed			31	19.08	not observed			0.670	61	20.19
	5	17	14.34				17	23.69				0.610	78	21.86
Domesticated emmer parent														
D26	1	59	41.00	50	1.00	1.00	59	40.01	50	1.00	1.00	1.041	not observed due to few grains	
	2	61	40.40	not observed			61	39.48	not observed			1.032		
	3	61	43.58				61	42.68				1.027		
	4	57	44.32				57	43.82				1.020		
	5	52	41.28				52	39.29				1.056		
F_1 grains on the maternal plants obtained from the reciprocal crosses between wild (W43) and domesticated (D26) emmer parents														
W43 x D26		9	17.71	9	0.89	0.88	9	23.50	9	1.00	1.00	0.753	14	23.05
D26 x W43		39	36.99	39	1.00	1.00	39	34.89	39	1.00	1.00	1.062	7	36.64
F_1 hybrids obtained from the reciprocal crosses between wild (W43) and domesticated (D26) emmer parents														
W43 x D26	A1	22	30.82	22	1.00	1.00	22	26.48	22	1.00	1.00	1.176	15	31.38
	B4	28	30.57	28	1.00	1.00	28	28.52	28	1.00	1.00	1.085	13	30.80
D26 x W43	A1	24	29.45	24	1.00	0.99	24	24.76	24	1.00	0.99	1.200	18	28.79
	A8	15	31.63	8	1.00	1.00	15	29.44	8	1.00	1.00	1.078	9	33.70
	B4	18	31.59	18	1.00	0.99	18	27.71	18	1.00	1.00	1.147	22	33.28

(to be contimuted)

Supplemental Table 6. (continued)

Line and Population No.	Plant No.	Two-grained spikelets: First florets: No. of grains measured	One-grain weight (mg)	No. of grains sown	GR	GI	Second florets: No. of grains measured	One-grain weight (mg)	No. of grains sown	GR	GI	GWR	One-grained spikelets: First florets: No. of grains measured	One-grain weight (mg)
F_2 populations derived from the cross combination of wild emmer parent (W43) x domesticated emmer parent (D26)														
12403A	2	52	32.36	50	1.00	1.00	52	29.17	50	1.00	1.00	1.117	not observed	
	3	23	39.03	23	1.00	1.00	23	35.25	23	1.00	1.00	1.111		
	4	51	37.85	50	1.00	1.00	51	34.56	50	1.00	1.00	1.109		
	5	33	40.43	33	1.00	1.00	33	32.18	33	1.00	1.00	1.263		
	6	32	34.48	32	1.00	1.00	32	29.44	32	1.00	1.00	1.185		
	7	25	35.40	25	1.00	1.00	25	29.52	25	1.00	1.00	1.199		
	8	27	32.01	25	1.00	1.00	27	28.34	25	1.00	1.00	1.129		
	9	12	30.65	12	1.00	1.00	12	26.97	12	1.00	1.00	1.137		
	10	29	38.06	25	1.00	1.00	29	34.84	25	1.00	1.00	1.092		
	11	30	37.13	25	1.00	1.00	30	33.39	25	1.00	1.00	1.112		
	12	25	40.35	25	1.00	1.00	25	37.22	25	1.00	1.00	1.084		
	13	30	34.96	25	1.00	1.00	30	37.59	25	1.00	1.00	0.930		
	14	26	31.56	25	1.00	1.00	26	28.18	25	1.00	1.00	1.120		
	15	26	33.90	25	1.00	1.00	26	37.38	25	1.00	1.00	0.907		
	16	28	33.14	25	1.00	1.00	28	34.45	25	1.00	1.00	0.962		
	17	17	46.66	17	1.00	1.00	17	43.36	17	1.00	1.00	1.076		
	18	26	38.43	25	1.00	1.00	26	36.78	25	1.00	1.00	1.045		
	19	32	19.11	25	0.92	0.87	32	33.97	25	1.00	1.00	0.572		
	20	38	35.48	25	1.00	1.00	38	33.23	25	1.00	1.00	1.068		
	21	25	34.34	25	1.00	1.00	25	29.18	25	1.00	1.00	1.177		
	22	29	30.55	25	1.00	1.00	29	32.18	25	1.00	1.00	0.949		
	23	27	25.70	25	1.00	1.00	27	27.57	25	1.00	1.00	0.932		

(to be contimuted)

Supplemental Table 6. (continued)

Line and Population No.	Plant No.	Two-grained spikelets											One-grained spikelets	
		First florets					Second florets						First florets	
		No. of grains measured	One-grain weight (mg)	No. of grains sown	GR	GI	No. of grains measured	One-grain weight (mg)	No. of grains sown	GR	GI	GWR	No. of grains measured	One-grain weight (mg)
	24	26	16.39	25	1.00	0.90	26	29.64	25	1.00	1.00	0.553	not observed	
	25	28	31.55	25	1.00	1.00	28	32.36	25	1.00	1.00	0.975		
	26	29	39.32	25	1.00	1.00	29	33.96	25	1.00	1.00	1.158		
	27	27	34.00	25	1.00	1.00	27	32.35	25	1.00	1.00	1.051		
	28	26	37.43	25	1.00	1.00	26	35.67	25	1.00	1.00	1.049		
	29	29	35.22	25	1.00	1.00	29	34.75	25	1.00	1.00	1.014		
	30	25	37.02	25	1.00	1.00	25	33.89	25	1.00	1.00	1.092		
	31	28	39.18	25	1.00	1.00	28	38.24	25	1.00	1.00	1.024		
	32	32	27.54	25	1.00	1.00	32	30.54	25	1.00	1.00	0.902		
	33	27	30.33	25	1.00	1.00	27	35.14	25	1.00	1.00	0.863		
	34	26	34.81	25	1.00	1.00	26	33.44	25	1.00	1.00	1.041		
	35	28	36.51	25	1.00	1.00	28	35.09	25	1.00	1.00	1.040		
	36	30	38.41	25	1.00	1.00	30	35.01	25	1.00	1.00	1.097		
	37	25	33.49	25	1.00	1.00	25	31.28	25	1.00	1.00	1.071		
	38	26	27.16	25	1.00	1.00	26	25.91	25	1.00	1.00	1.048		
	39	29	14.77	25	0.68	0.51	29	25.91	25	1.00	1.00	0.580		
	41	27	33.63	25	1.00	1.00	27	27.97	25	1.00	1.00	1.202		
	42	27	37.74	25	1.00	1.00	27	35.32	25	1.00	1.00	1.069		
	43	29	40.41	25	1.00	1.00	29	37.02	25	1.00	1.00	1.092		
	44	26	32.22	25	1.00	1.00	26	30.91	25	1.00	1.00	1.043		
	45	28	42.95	25	1.00	1.00	28	37.89	25	1.00	1.00	1.134		
	46	27	41.10	25	1.00	1.00	27	35.97	25	1.00	1.00	1.143		
	47	35	30.16	25	1.00	1.00	35	33.65	25	1.00	1.00	0.896		

(to be contimuted)

Supplemental Table 6. (continued)

Line and Population No.	Plant No.	Two-grained spikelets: First florets: No. of grains measured	One-grain weight (mg)	No. of grains sown	GR	GI	Second florets: No. of grains measured	One-grain weight (mg)	No. of grains sown	GR	GI	GWR	One-grained spikelets: First florets: No. of grains measured	One-grain weight (mg)
	48	33	37.25	25	1.00	1.00	33	35.87	25	1.00	1.00	1.038	not observed	
	49	28	36.74	25	1.00	1.00	28	36.44	25	1.00	1.00	1.008		
	50	25	48.13	25	1.00	1.00	25	45.54	25	1.00	1.00	1.057		
	51	27	36.62	25	1.00	1.00	27	36.13	25	1.00	1.00	1.014		
	52	28	36.42	25	1.00	1.00	28	32.75	25	1.00	1.00	1.112		
	53	18	43.21	18	1.00	1.00	18	39.20	18	1.00	1.00	1.102		
	54	28	35.36	25	1.00	1.00	28	31.67	25	1.00	1.00	1.117		
	55	19	34.98	19	1.00	1.00	19	34.78	19	1.00	1.00	1.006		
	56	31	36.36	25	1.00	1.00	31	39.88	25	1.00	1.00	0.912		
	57	28	42.44	25	1.00	1.00	28	39.72	25	1.00	1.00	1.069		
	58	27	21.88	25	0.88	0.78	27	41.41	25	1.00	1.00	0.528		
	59	26	37.83	25	1.00	1.00	26	36.71	25	1.00	1.00	1.031		
	60	26	37.48	25	1.00	1.00	26	38.69	25	1.00	1.00	0.969		
12403B	1	31	33.16	25	1.00	1.00	31	28.81	25	1.00	1.00	1.151		
	2	27	42.53	25	1.00	1.00	27	44.23	25	1.00	1.00	0.962		
	3	28	39.48	25	1.00	1.00	28	37.64	25	1.00	1.00	1.049		
	4	30	39.35	25	1.00	1.00	30	36.89	25	1.00	1.00	1.067		
	5	28	42.55	25	1.00	1.00	28	38.81	25	1.00	1.00	1.097		
	6	25	32.60	25	1.00	1.00	25	29.82	25	1.00	1.00	1.093		
	7	27	34.38	24	1.00	1.00	27	29.95	25	1.00	1.00	1.148		
	8	27	35.16	25	1.00	1.00	27	32.03	25	1.00	1.00	1.097		
	9	29	33.46	25	1.00	1.00	29	30.93	25	1.00	1.00	1.082		
	10	31	37.98	25	1.00	1.00	31	35.83	25	1.00	1.00	1.060		

(to be contimuted)

Supplemental Table 6. (continued)

Line and Population No.	Plant No.	Two-grained spikelets: First florets: No. of grains measured	One-grain weight (mg)	No. of grains sown	GR	GI	Second florets: No. of grains measured	One-grain weight (mg)	No. of grains sown	GR	GI	GWR	One-grained spikelets: First florets: No. of grains measured	One-grain weight (mg)
	11	28	25.90	25	1.00	1.00	28	36.49	25	1.00	1.00	0.710	not observed	
	13	27	35.36	25	1.00	1.00	27	31.33	25	1.00	1.00	1.129		
	14	28	18.32	25	0.76	0.68	28	32.53	25	1.00	1.00	0.562		
	15	26	40.06	25	1.00	1.00	26	37.45	25	1.00	1.00	1.070		
	16	25	40.56	25	1.00	1.00	25	39.35	25	1.00	1.00	1.031		
	17	32	42.32	25	1.00	1.00	32	38.16	25	1.00	1.00	1.109		
	18	24	35.18	24	1.00	1.00	24	30.55	24	1.00	1.00	1.152		
	19	35	30.54	25	1.00	1.00	35	32.52	25	1.00	1.00	0.939		
	20	22	40.45	22	1.00	1.00	22	36.25	22	1.00	1.00	1.116		
	21	19	39.74	19	1.00	1.00	19	36.27	19	1.00	1.00	1.096		
	22	25	26.21	25	1.00	0.99	25	37.04	25	1.00	1.00	0.708		
	24	29	33.40	25	1.00	1.00	29	34.34	25	1.00	1.00	0.973		
	25	28	33.36	25	1.00	1.00	28	28.67	25	1.00	1.00	1.164		
	26	26	26.03	25	1.00	1.00	26	22.72	25	1.00	1.00	1.146		
	27	28	39.86	25	1.00	1.00	28	37.11	25	1.00	1.00	1.074		
	28	29	40.23	25	1.00	0.99	29	35.11	25	1.00	1.00	1.146		
	29	28	36.35	25	1.00	0.98	28	35.01	25	1.00	1.00	1.038		
	30	28	27.69	25	1.00	1.00	28	28.06	25	1.00	1.00	0.987		

(to be contimuted)

Supplemental Table 6. (continued)

Line and Population No.	Plant No.	Two-grained spikelets: First florets: No. of grains measured	One-grain weight (mg)	No. of grains sown	GR	GI	Second florets: No. of grains measured	One-grain weight (mg)	No. of grains sown	GR	GI	GWR	One-grained spikelets: First florets: No. of grains measured	One-grain weight (mg)
F_2 populations derived from the cross combination of domesticated emmer parent (D26) x wild emmer parent (W43)														
12404A	1	16	37.56	16	1.00	1.00	16	33.06	16	1.00	1.00	1.156	not observed	
	2	24	41.66	24	1.00	1.00	24	38.34	24	1.00	1.00	1.097		
	3	43	32.23	42	1.00	1.00	43	26.44	42	1.00	0.99	1.251		
	4	42	36.38	42	1.00	1.00	42	38.48	42	1.00	1.00	0.970		
	5	50	31.51	50	1.00	1.00	50	26.31	50	1.00	1.00	1.215		
	6	32	31.86	25	1.00	1.00	32	26.97	25	1.00	1.00	1.181		
	7	26	31.85	25	1.00	1.00	26	30.02	25	1.00	1.00	1.061		
	8	31	42.62	25	1.00	1.00	31	42.96	25	1.00	1.00	0.992		
	9	27	36.17	25	1.00	1.00	27	31.88	25	1.00	1.00	1.135		
	10	24	28.84	24	1.00	0.99	24	36.00	24	1.00	1.00	0.801		
	11	12	43.42	12	1.00	0.88	12	42.30	12	1.00	0.88	1.026		
	12	33	24.68	25	1.00	1.00	33	28.95	25	1.00	1.00	0.850		
	13	31	31.97	25	1.00	1.00	31	28.91	25	1.00	0.99	1.106		
	14	30	31.09	25	1.00	1.00	30	28.17	25	1.00	1.00	1.104		
	15	30	29.23	25	1.00	1.00	30	29.16	25	1.00	0.98	1.003		
	16	30	17.16	25	0.92	0.83	30	33.18	25	1.00	1.00	0.517		
	17	25	38.88	25	1.00	1.00	25	36.70	25	1.00	1.00	1.059		
	18	28	29.39	25	1.00	0.99	28	26.51	25	1.00	1.00	1.109		
	19	28	34.63	25	1.00	1.00	28	33.39	25	1.00	1.00	1.037		
	20	26	25.00	25	1.00	1.00	26	29.72	25	1.00	1.00	0.841		
	21	26	36.69	25	1.00	0.89	26	33.43	25	1.00	0.95	1.097		
	22	29	26.47	25	1.00	0.88	29	36.35	25	1.00	0.95	0.728		
	23	30	34.32	25	1.00	1.00	30	31.92	25	1.00	1.00	1.075		

(to be contimuted)

Supplemental Table 6. (continued)

Line and Population No.	Plant No.	Two-grained spikelets: First florets: No. of grains measured	One-grain weight (mg)	No. of grains sown	GR	GI	Second florets: No. of grains measured	One-grain weight (mg)	No. of grains sown	GR	GI	GWR	One-grained spikelets: First florets: No. of grains measured	One-grain weight (mg)
	24	30	31.94	25	1.00	1.00	30	32.08	25	1.00	1.00	0.996	not observed	
	25	30	22.90	25	1.00	1.00	30	30.38	25	1.00	1.00	0.754		
	26	29	37.54	25	1.00	0.98	29	35.57	25	1.00	0.98	1.055		
	27	25	38.55	25	1.00	0.88	25	37.46	25	1.00	0.88	1.029		
	28	31	32.59	25	1.00	0.88	31	34.72	25	1.00	0.88	0.939		
	29	27	30.43	25	1.00	1.00	27	37.25	25	1.00	1.00	0.817		
	30	34	43.88	25	1.00	0.97	34	36.78	25	1.00	0.94	1.193		
	31	31	29.96	25	1.00	1.00	31	30.87	25	1.00	1.00	0.971		
	32	30	25.59	24	1.00	0.88	30	31.92	25	1.00	0.88	0.802		
	33	25	34.91	25	1.00	1.00	25	29.24	25	1.00	1.00	1.194		
	34	31	35.89	25	1.00	1.00	31	29.75	25	1.00	1.00	1.206		
	35	27	31.09	25	1.00	1.00	27	30.65	25	1.00	1.00	1.014		
	36	32	26.78	25	1.00	1.00	32	28.09	25	1.00	1.00	0.953		
	37	34	22.99	25	1.00	0.95	34	33.02	25	1.00	0.99	0.696		
	38	32	39.03	25	1.00	0.88	32	36.27	25	1.00	0.90	1.076		
	39	26	27.93	25	1.00	1.00	26	23.13	25	1.00	1.00	1.208		
	40	26	24.21	25	1.00	0.88	26	35.11	25	1.00	0.90	0.690		
	41	24	30.93	25	1.00	1.00	24	28.93	25	1.00	1.00	1.069		
	42	10	32.19	10	1.00	1.00	10	31.81	10	1.00	1.00	1.012		
	43	30	32.93	25	1.00	1.00	30	27.81	25	1.00	1.00	1.184		
	44	27	34.84	25	1.00	1.00	27	31.55	25	1.00	1.00	1.104		
	45	26	35.93	25	1.00	1.00	26	34.55	25	1.00	1.00	1.040		
	46	30	37.57	25	1.00	0.99	30	37.08	25	1.00	1.00	1.013		

(to be contimuted)

Supplemental Table 6. (continued)

Line and Population No.	Plant No.	Two-grained spikelets: First florets: No. of grains measured	One-grain weight (mg)	No. of grains sown	GR	GI	Second florets: No. of grains measured	One-grain weight (mg)	No. of grains sown	GR	GI	GWR	One-grained spikelets: First florets: No. of grains measured	One-grain weight (mg)
	47	30	17.18	25	1.00	0.93	30	23.43	25	1.00	1.00	0.737	not observed	
	48	12	33.58	12	1.00	0.95	12	26.00	12	1.00	1.00	1.291		
	49	25	42.82	25	1.00	0.94	25	36.66	25	1.00	0.97	1.168		
	50	20	31.69	20	1.00	1.00	20	27.05	20	1.00	1.00	1.172		
	51	30	28.36	25	1.00	1.00	30	26.95	25	1.00	1.00	1.052		
	52	23	39.15	22	1.00	1.00	23	33.90	22	1.00	1.00	1.155		
	53	13	39.75	13	1.00	0.99	13	33.08	13	1.00	1.00	1.202		
	54	28	38.70	25	1.00	1.00	28	36.38	25	1.00	0.98	1.064		
	55	29	29.36	25	1.00	1.00	29	28.71	25	1.00	1.00	1.023		
	56	25	35.65	25	1.00	1.00	25	29.70	25	1.00	1.00	1.200		
	57	26	26.08	25	1.00	1.00	26	24.50	25	1.00	1.00	1.065		
12404B	1	32	25.17	25	1.00	1.00	32	28.18	25	1.00	1.00	0.893		
	2	28	23.56	25	1.00	1.00	28	22.42	25	1.00	1.00	1.051		
	3	29	35.79	25	1.00	1.00	29	31.52	25	1.00	1.00	1.136		
	4	27	33.81	25	1.00	1.00	27	31.83	25	1.00	1.00	1.062		
	5	26	35.00	25	1.00	1.00	26	30.67	25	1.00	1.00	1.141		
	7	26	38.52	25	1.00	1.00	26	34.29	25	1.00	1.00	1.124		
	8	33	35.00	25	1.00	1.00	33	30.09	25	1.00	1.00	1.163		
	10	25	41.90	25	1.00	1.00	25	36.25	25	1.00	1.00	1.156		
	11	26	35.68	25	1.00	1.00	26	33.09	25	1.00	1.00	1.078		
	12	31	27.12	25	1.00	1.00	31	26.44	25	1.00	1.00	1.025		
	13	14	36.54	14	1.00	0.96	14	33.50	14	1.00	0.96	1.091		

(to be contimuted)

Supplemental Table 6. (continued)

Line and Population No.	Plant No.	Two-grained spikelets: First florets: No. of grains measured	One-grain weight (mg)	No. of grains sown	GR	GI	Second florets: No. of grains measured	One-grain weight (mg)	No. of grains sown	GR	GI	GWR	One-grained spikelets: First florets: No. of grains measured	One-grain weight (mg)
	14	29	29.02	25	1.00	1.00	29	34.81	25	1.00	1.00	0.834	not observed	
	15	26	37.96	25	1.00	0.99	26	34.15	25	1.00	1.00	1.112		
	17	37	22.23	25	1.00	0.88	37	29.13	25	1.00	0.88	0.770		
	18	31	36.44	25	1.00	1.00	31	30.54	25	1.00	1.00	1.193		
	19	28	16.12	25	0.52	0.48	28	33.99	25	1.00	1.00	0.478		
	20	37	33.43	25	1.00	0.94	37	27.37	25	1.00	0.99	1.221		
	21	22	47.12	22	1.00	0.99	22	43.57	22	1.00	0.99	1.081		
	22	25	32.21	25	1.00	1.00	25	31.88	25	1.00	1.00	1.010		
	23	25	28.52	25	1.00	0.92	25	34.46	25	1.00	0.97	0.828		
	24	30	39.71	25	1.00	0.92	30	42.58	25	1.00	0.99	0.933		
	25	33	36.57	25	1.00	0.92	33	35.62	25	1.00	0.93	1.026		
	26	24	32.25	24	1.00	0.99	24	28.94	24	1.00	0.99	1.114		
	27	25	29.87	25	1.00	0.99	25	30.92	25	1.00	1.00	0.966		
	28	29	43.61	25	1.00	1.00	29	43.22	25	1.00	1.00	1.009		
	29	29	18.58	25	0.92	0.81	29	30.22	25	1.00	1.00	0.619		
	30	30	27.54	25	1.00	1.00	30	31.61	25	1.00	1.00	0.888		

Supplemental Table 7. One-grain weights of the three grain groups and GWR values of two-grained spikelets in wild and domesticated parental lines, their reciprocal F_1 hybrids, and F_2 populations derived from self-pollination of the F_1 hybrids (observed in 2013)

		Two-grained spikelets													One-grained spikelets	
		Replicate 1					Replicate 2					Averages				
		First florets		Second florets			First florets		Second florets			One-grain weight (mg)			First florets	
Line and Population No.	Plant No.	No. of grains measured	One-grain weight (mg)	No. of grains measured	One-grain weight (mg)	GWR	No. of grains measured	One-grain weight (mg)	No. of grains measured	One-grain weight (mg)	GWR	First florets	Second florets	GWR	No. of grains measured	One-grain weight (mg)
Wild emmer parent																
W43	1	15	25.7	15	39.0	0.658	15	25.2	15	38.9	0.649	25.4	39.0	0.653	15	36.0
	2	20	22.4	20	35.4	0.634	20	20.8	20	35.8	0.580	21.6	35.6	0.607	20	35.5
	3	20	21.4	20	31.4	0.681	20	20.2	20	27.8	0.728	20.8	29.6	0.705	15	30.6
	6	20	19.7	20	36.2	0.543	20	22.5	20	33.7	0.669	21.1	34.9	0.606	15	31.8
Domesticated emmer parent																
D26	1	25	39.0	25	37.0	1.054	25	38.1	25	38.2	0.996	38.6	37.6	1.025	10	34.2
	2	25	40.7	25	40.5	1.007	25	39.6	25	36.8	1.074	40.1	38.7	1.040	20	34.3
	8	25	42.3	25	41.0	1.032	25	40.4	25	40.1	1.007	41.4	40.6	1.019	20	35.2
	9	25	39.1	25	38.5	1.015	25	32.0	25	31.4	1.017	35.5	35.0	1.016	10	27.7
F_1 hybrids obtained from the reciprocal crosses between wild (W43) and domesticated (D26) emmer parents																
W43 x D26	1	25	41.3	25	40.9	1.011	25	40.9	25	39.9	1.025	41.1	40.4	1.018	15	39.7
	2	25	41.8	25	41.5	1.008	25	40.6	25	41.3	0.983	41.2	41.4	0.995	20	38.8
	4	25	41.5	25	39.9	1.041	25	41.3	25	39.0	1.061	41.4	39.4	1.051	20	41.6
	5	25	43.3	25	41.5	1.043	25	40.3	25	39.7	1.015	41.8	40.6	1.029	20	44.0
D26 x W43	1	25	40.6	25	42.3	0.960	25	43.0	25	40.3	1.068	41.8	41.3	1.014	15	43.8
	2	25	40.1	25	39.1	1.027	25	37.0	25	36.1	1.025	38.6	37.6	1.026	20	42.2
	3	20	43.7	20	42.0	1.040	20	42.1	20	41.6	1.014	42.9	41.8	1.027	20	47.5
	4	20	43.9	20	41.9	1.047	20	41.0	20	40.4	1.017	42.4	41.1	1.032	15	43.8

(to be continued)

Supplemental Table 7. (continued)

Line and Population No.	Plant No.	Two-grained spikelets														One-grained spikelets	
		Replicate 1					Replicate 2					Averages					
		First florets		Second florets			First florets		Second florets			One-grain weight (mg)				First florets	
		No. of grains measured	One-grain weight (mg)	No. of grains measured	One-grain weight (mg)	GWR	No. of grains measured	One-grain weight (mg)	No. of grains measured	One-grain weight (mg)	GWR	First florets	Second florets	GWR		No. of grains measured	One-grain weight (mg)
F_2 population derived from the cross combination of wild emmer parent (W43) x domesticated emmer parent (D26)																	
13405	1	25	39.3	25	39.1	1.005	25	39.1	25	38.9	1.006	39.2	39.0	1.005		20	31.1
	2	25	38.8	25	39.1	0.992	25	41.2	25	41.1	1.002	40.0	40.1	0.997		20	37.0
	3	15	47.8	15	51.1	0.935	15	49.7	15	51.5	0.964	48.7	51.3	0.949		20	42.3
	5	25	31.4	25	32.9	0.954	25	33.5	25	36.3	0.922	32.4	34.6	0.938		15	28.6
	8	25	45.7	25	45.8	0.997	25	46.2	25	44.6	1.036	45.9	45.2	1.016		20	44.4
	9	25	40.4	25	36.0	1.124	25	40.9	25	36.4	1.125	40.7	36.2	1.124		15	33.6
	10	25	40.2	25	36.8	1.091	25	40.3	25	36.4	1.109	40.3	36.6	1.100		20	30.5
	11	25	36.7	25	39.5	0.929	25	38.6	25	39.7	0.974	37.7	39.6	0.951		10	34.8
	12	25	40.5	25	41.7	0.969	25	40.8	25	39.6	1.031	40.7	40.7	1.000		20	38.7
	13	15	42.9	15	45.2	0.949	15	39.3	15	45.9	0.856	41.1	45.6	0.903		15	41.1
	14	25	42.8	25	41.8	1.025	25	38.5	25	40.3	0.955	40.6	41.0	0.990		20	41.1
	15	25	39.7	25	37.9	1.047	25	39.3	25	37.9	1.039	39.5	37.9	1.043		15	35.5
	16	15	55.7	15	54.4	1.024	15	57.4	15	58.4	0.982	56.5	56.4	1.003		15	54.2
	17	25	48.9	25	50.2	0.974	25	48.4	25	49.8	0.972	48.6	50.0	0.973		10	83.2
	18	25	30.6	25	39.2	0.780	25	30.7	25	39.6	0.776	30.6	39.4	0.778		25	26.2
	19	25	41.0	25	40.2	1.022	25	38.6	25	42.7	0.904	39.8	41.4	0.963		10	38.1
	20	25	41.3	25	37.8	1.095	25	39.9	25	37.6	1.061	40.6	37.7	1.078		20	37.1
	22	10	45.5	10	43.9	1.035	10	47.5	10	45.9	1.034	46.5	44.9	1.035		15	46.3
	24	25	31.8	25	40.9	0.778	25	29.3	25	41.4	0.707	30.6	41.2	0.743		5	27.9
	25	25	42.0	25	41.4	1.015	25	43.4	25	37.9	1.145	42.7	39.6	1.080		10	39.0
	26	25	23.9	25	35.7	0.669	25	27.2	25	36.5	0.745	25.5	36.1	0.707		15	29.3
	27	25	49.2	25	51.7	0.952	25	48.8	25	49.3	0.990	49.0	50.5	0.971		15	5.0
	28	15	48.4	15	49.8	0.973	15	49.9	15	49.1	1.016	49.2	49.4	0.995		15	49.0
	29	15	47.0	15	48.6	0.968	15	48.2	15	50.0	0.963	47.6	49.3	0.965		15	46.9
	30	25	48.4	25	46.7	1.037	25	46.9	25	50.5	0.928	47.7	48.6	0.982		20	47.8
	31	25	46.8	25	49.3	0.950	25	48.4	25	46.7	1.037	47.6	48.0	0.993		10	40.5
	33	25	50.1	25	55.3	0.906	25	51.0	25	50.8	1.005	50.6	53.0	0.955		10	42.6
	34	25	38.1	25	35.6	1.071	25	36.3	25	36.3	1.002	37.2	35.9	1.037		20	34.2

(to be continued)

Supplemental Table 7. (continued)

Line and Population No.	Plant No.	Two-grained spikelets													One-grained spikelets	
		Replicate 1					Replicate 2					Averages			First florets	
		First florets		Second florets			First florets		Second florets			One-grain weight (mg)				
		No. of grains measured	One-grain weight (mg)	No. of grains measured	One-grain weight (mg)	GWR	No. of grains measured	One-grain weight (mg)	No. of grains measured	One-grain weight (mg)	GWR	First florets	Second florets	GWR	No. of grains measured	One-grain weight (mg)
13405	35	25	35.5	25	36.6	0.970	25	36.0	25	33.6	1.073	35.7	35.1	1.021	20	27.3
	36	25	47.7	25	47.0	1.016	25	47.6	25	48.0	0.990	47.6	47.5	1.003	20	43.2
	37	25	45.5	25	46.7	0.975	25	43.8	25	39.0	1.123	44.7	42.8	1.049	20	47.3
	38	25	44.0	25	45.5	0.969	25	46.2	25	45.8	1.008	45.1	45.6	0.989	20	40.5
	39	25	38.8	25	40.0	0.969	25	36.4	25	39.0	0.932	37.6	39.5	0.950	20	34.5
	40	25	38.7	25	39.4	0.983	25	38.2	25	38.6	0.989	38.4	39.0	0.986	15	29.5
	41	25	38.7	25	40.2	0.963	25	37.9	25	41.1	0.922	38.3	40.6	0.942	15	32.3
	43	25	44.7	25	43.4	1.032	25	44.7	25	45.0	0.994	44.7	44.2	1.013	5	36.2
	44	25	40.2	25	40.0	1.006	25	41.2	25	39.3	1.048	40.7	39.6	1.027	20	34.8
	45	25	49.9	25	50.0	0.998	25	51.3	25	48.9	1.049	50.6	49.4	1.023	15	41.9
	46	25	22.3	25	37.1	0.600	25	22.9	25	38.4	0.598	22.6	37.7	0.599	10	29.6
	47	25	35.3	25	47.7	0.742	25	32.8	25	47.2	0.695	34.1	47.4	0.718	20	33.6
	48	25	48.3	25	42.5	1.138	25	46.9	25	45.0	1.042	47.6	43.7	1.090	20	44.0
	49	25	48.7	25	51.9	0.938	25	47.1	25	51.0	0.925	47.9	51.4	0.932	5	34.5
	50	25	26.1	25	33.5	0.780	25	30.0	25	33.5	0.897	28.1	33.5	0.838	10	26.4
	51	20	45.8	20	43.2	1.058	20	46.0	20	41.8	1.101	45.9	42.5	1.079	20	45.4
	52	25	22.0	25	32.5	0.675	25	22.1	25	30.3	0.727	22.0	31.4	0.701	20	22.0
	53	25	38.1	25	38.9	0.981	25	38.9	25	37.5	1.037	38.5	38.2	1.009	15	30.2
	54	25	44.5	25	46.7	0.953	25	45.2	25	44.8	1.010	44.9	45.8	0.981	15	41.6
	55	25	42.9	25	41.3	1.037	25	43.9	25	41.8	1.048	43.4	41.6	1.043	20	33.4
	56	15	52.8	15	50.3	1.049	15	50.0	15	47.5	1.054	51.4	48.9	1.052	20	48.5
	57	25	30.6	25	43.3	0.706	25	26.9	25	42.1	0.639	28.7	42.7	0.672	20	42.2
	58	25	35.9	25	36.7	0.979	25	37.6	25	38.5	0.977	36.7	37.6	0.978	20	38.6
	59	25	39.1	25	35.8	1.091	25	38.5	25	34.9	1.101	38.8	35.4	1.096	20	35.1
	61	25	33.5	25	38.4	0.871	25	35.2	25	37.7	0.933	34.3	38.1	0.902	20	33.6
	62	25	38.4	25	42.1	0.911	25	38.2	25	41.0	0.933	38.3	41.5	0.922	20	41.0
	63	25	39.6	25	37.3	1.062	25	38.0	25	37.3	1.019	38.8	37.3	1.040	20	33.2
	64	25	42.2	25	38.1	1.107	25	39.6	25	38.8	1.021	40.9	38.4	1.064	10	36.4
	66	25	42.4	25	43.2	0.980	25	44.4	25	43.3	1.025	43.4	43.3	1.002	20	37.8
	67	25	43.6	25	45.0	0.969	25	42.1	25	40.9	1.030	42.9	42.9	0.999	20	41.0

(to be continued)

Supplemental Table 7. (continued)

Line and Population No.	Plant No.	Two-grained spikelets: Replicate 1: First florets: No. of grains measured	Two-grained spikelets: Replicate 1: First florets: One-grain weight (mg)	Two-grained spikelets: Replicate 1: Second florets: No. of grains measured	Two-grained spikelets: Replicate 1: Second florets: One-grain weight (mg)	Two-grained spikelets: Replicate 1: GWR	Two-grained spikelets: Replicate 2: First florets: No. of grains measured	Two-grained spikelets: Replicate 2: First florets: One-grain weight (mg)	Two-grained spikelets: Replicate 2: Second florets: No. of grains measured	Two-grained spikelets: Replicate 2: Second florets: One-grain weight (mg)	Two-grained spikelets: Replicate 2: GWR	Two-grained spikelets: Averages: One-grain weight (mg): First florets	Two-grained spikelets: Averages: One-grain weight (mg): Second florets	Two-grained spikelets: Averages: GWR	One-grained spikelets: First florets: No. of grains measured	One-grained spikelets: First florets: One-grain weight (mg)
13405	69	15	48.1	15	45.0	1.067	15	47.2	15	47.0	1.003	47.6	46.0	1.035	15	44.8
	70	15	48.7	15	46.9	1.038	15	51.7	15	46.8	1.104	50.2	46.8	1.071	15	48.5
	71	25	43.7	25	42.4	1.030	25	44.7	25	40.1	1.115	44.2	41.3	1.073	20	42.9
	72	25	40.7	25	40.7	1.000	25	39.6	25	42.0	0.942	40.2	41.4	0.971	20	39.8
	73	25	42.1	25	39.7	1.063	25	43.2	25	41.1	1.052	42.7	40.4	1.057	20	31.1
	74	25	39.1	25	33.8	1.154	25	39.7	25	35.0	1.134	39.4	34.4	1.144	20	36.6
	75	25	48.7	25	47.6	1.023	25	48.5	25	49.5	0.979	48.6	48.5	1.001	20	46.7
	76	25	44.6	25	48.2	0.925	25	45.1	25	47.0	0.959	44.8	47.6	0.942	20	40.9
	77	25	33.3	25	33.0	1.009	25	35.7	25	35.1	1.017	34.5	34.1	1.013	20	29.0
	78	25	39.1	25	41.2	0.948	25	40.6	25	39.2	1.035	39.8	40.2	0.991	20	31.9
	79	25	46.2	25	50.5	0.915	25	49.0	25	48.5	1.010	47.6	49.5	0.962	15	37.2
	80	25	43.5	25	45.6	0.955	25	43.4	25	44.0	0.987	43.5	44.8	0.971	20	35.3
	81	25	40.2	25	35.4	1.133	25	38.5	25	34.7	1.109	39.3	35.1	1.121	20	38.8
	82	25	41.3	25	42.4	0.972	25	41.5	25	45.7	0.909	41.4	44.0	0.941	15	32.8
	83	25	37.4	25	43.8	0.854	25	34.8	25	43.2	0.807	36.1	43.5	0.830	20	38.5
	84	25	33.5	25	29.7	1.128	25	33.8	25	30.4	1.112	33.6	30.0	1.120	20	26.2
	85	25	38.9	25	40.0	0.974	25	38.7	25	36.7	1.053	38.8	38.3	1.014	20	42.6
	86	25	44.9	25	43.4	1.035	25	44.3	25	42.1	1.053	44.6	42.7	1.044	20	43.6
	87	20	42.1	20	39.5	1.064	20	41.8	20	40.4	1.034	41.9	40.0	1.049	20	40.5
	89	25	41.4	25	42.3	0.980	25	38.0	25	42.7	0.891	39.7	42.5	0.935	20	37.8
	90	25	49.9	25	51.8	0.963	25	51.8	25	49.1	1.054	50.8	50.5	1.009	20	50.5
	91	20	51.2	20	51.9	0.988	20	48.9	20	48.9	1.001	50.1	50.4	0.994	20	49.7
	92	25	45.6	25	47.0	0.970	25	44.4	25	46.7	0.951	45.0	46.9	0.961	15	32.4
	93	25	52.3	25	52.2	1.003	25	52.6	25	51.4	1.022	52.4	51.8	1.013	20	51.7
	94	25	23.7	25	43.9	0.540	25	24.9	25	39.3	0.635	24.3	41.6	0.588	15	29.0
	95	25	40.3	25	39.3	1.025	25	38.5	25	36.8	1.046	39.4	38.0	1.036	20	43.1

(to be continued)

Supplemental Table 7. (continued)

Line and Population No.	Plant No.	Two-grained spikelets: Replicate 1: First florets: No. of grains measured	One-grain weight (mg)	Second florets: No. of grains measured	One-grain weight (mg)	GWR	Replicate 2: First florets: No. of grains measured	One-grain weight (mg)	Second florets: No. of grains measured	One-grain weight (mg)	GWR	Averages: One-grain weight (mg): First florets	Second florets	GWR	One-grained spikelets: First florets: No. of grains measured	One-grain weight (mg)
F_2 population derived from the cross combination of domesticated emmer parent (D26) x wild emmer parent (W43)																
13406	1	25	35.2	25	33.9	1.040	25	34.6	25	34.3	1.010	34.9	34.1	1.025	20	29.9
	2	25	20.9	25	34.8	0.602	25	21.4	25	34.0	0.630	21.2	34.4	0.616	20	30.1
	3	25	48.9	25	47.8	1.023	25	46.6	25	46.5	1.001	47.7	47.2	1.012	20	49.9
	5	25	42.0	25	41.1	1.022	25	46.4	25	42.8	1.084	44.2	42.0	1.053	20	46.9
	6	25	32.0	25	35.0	0.914	25	31.8	25	31.2	1.018	31.9	33.1	0.966	20	29.4
	7	25	44.4	25	43.6	1.019	25	42.6	25	42.9	0.992	43.5	43.2	1.006	20	41.2
	8	25	46.0	25	46.6	0.986	25	46.1	25	44.7	1.030	46.0	45.7	1.008	20	39.8
	9	25	47.5	25	48.2	0.986	25	45.7	25	46.8	0.977	46.6	47.5	0.982	20	45.1
	10	25	45.3	25	49.4	0.916	25	48.5	25	44.8	1.083	46.9	47.1	0.999	15	44.4
	11	25	33.9	25	37.2	0.913	25	34.2	25	35.1	0.975	34.1	36.1	0.944	20	36.9
	13	25	37.9	25	36.2	1.047	25	31.9	25	33.3	0.959	34.9	34.7	1.003	15	27.1
	14	15	52.1	15	48.3	1.079	15	49.3	15	50.2	0.981	50.7	49.2	1.030	20	46.8
	15	25	42.1	25	42.9	0.983	25	42.5	25	39.7	1.069	42.3	41.3	1.026	20	36.8
	16	25	38.2	25	40.8	0.938	25	37.4	25	40.4	0.926	37.8	40.6	0.932	15	33.3
	17	25	42.6	25	40.5	1.051	25	41.2	25	39.5	1.043	41.9	40.0	1.047	15	52.7
	18	25	21.6	25	42.3	0.509	25	23.0	25	42.1	0.547	22.3	42.2	0.528	10	28.6
	19	20	47.2	20	49.6	0.952	20	49.9	20	49.2	1.014	48.5	49.4	0.983	20	49.2
	20	15	23.6	15	37.5	0.629	15	21.8	15	34.5	0.630	22.7	36.0	0.630	20	33.6
	21	20	46.0	20	48.2	0.956	20	46.7	20	45.9	1.017	46.4	47.1	0.986	20	45.3
	22	20	46.9	20	42.4	1.107	20	41.1	20	43.7	0.940	44.0	43.1	1.024	20	40.1
	23	20	47.1	20	38.5	1.223	20	44.3	20	38.8	1.142	45.7	38.6	1.182	20	48.0
	24	25	31.6	25	39.6	0.797	25	27.9	25	35.9	0.778	29.8	37.8	0.787	15	32.6
	26	15	49.4	15	51.7	0.955	15	43.1	15	49.6	0.870	46.2	50.6	0.912	20	51.8
	27	25	41.7	25	40.7	1.024	25	40.6	25	38.4	1.057	41.1	39.6	1.040	20	40.2
	28	20	55.2	20	55.1	1.002	20	52.7	20	53.7	0.980	53.9	54.4	0.991	20	52.8
	29	25	31.6	25	40.7	0.778	25	35.3	25	38.3	0.923	33.5	39.5	0.850	20	38.9
	30	25	17.4	25	22.4	0.777	25	17.4	25	20.8	0.838	17.4	21.6	0.808	20	21.4
	31	15	24.6	15	23.0	1.071	15	24.5	15	23.0	1.063	24.6	23.0	1.067	20	26.9

(to be continued)

Supplemental Table 7. (continued)

		Two-grained spikelets													One-grained spikelets	
		Replicate 1					Replicate 2					Averages				
		First florets		Second florets			First florets		Second florets			One-grain weight (mg)			First florets	
Line and Population No.	Plant No.	No. of grains measured	One-grain weight (mg)	No. of grains measured	One-grain weight (mg)	GWR	No. of grains measured	One-grain weight (mg)	No. of grains measured	One-grain weight (mg)	GWR	First florets	Second florets	GWR	No. of grains measured	One-grain weight (mg)
13406	32	25	40.9	25	39.7	1.028	25	44.5	25	39.4	1.131	42.7	39.5	1.079	20	36.7
	33	25	43.3	25	44.1	0.982	25	45.0	25	43.1	1.044	44.1	43.6	1.013	15	43.4
	34	25	46.9	25	48.8	0.963	25	48.2	25	48.0	1.004	47.6	48.4	0.984	5	24.7
	35	25	37.0	25	34.7	1.065	25	35.7	25	36.2	0.988	36.4	35.5	1.027	20	35.1
	36	25	47.6	25	45.2	1.051	25	47.5	25	43.7	1.088	47.6	44.5	1.069	20	50.0
	37	25	44.8	25	43.6	1.028	25	43.8	25	41.3	1.060	44.3	42.5	1.044	20	41.2
	38	25	40.4	25	45.8	0.882	25	44.3	25	46.3	0.957	42.3	46.1	0.919	15	40.3
	39	25	37.1	25	34.7	1.071	25	36.0	25	32.6	1.105	36.6	33.6	1.088	20	35.0
	40	25	22.2	25	43.2	0.515	25	23.1	25	43.9	0.525	22.6	43.5	0.520	20	41.6
	41	25	35.2	25	38.4	0.917	25	30.3	25	38.0	0.798	32.8	38.2	0.857	10	26.1
	42	25	45.9	25	44.1	1.039	25	42.7	25	43.4	0.983	44.3	43.8	1.011	10	37.2
	43	15	39.2	15	39.4	0.995	15	42.2	15	41.7	1.012	40.7	40.5	1.004	20	41.8
	44	25	42.8	25	41.6	1.029	25	39.9	25	40.7	0.981	41.3	41.1	1.005	20	42.3
	47	25	35.7	25	35.6	1.002	25	32.7	25	33.3	0.982	34.2	34.5	0.992	20	33.0
	48	25	40.9	25	37.5	1.089	25	40.0	25	35.4	1.130	40.4	36.5	1.109	20	37.3
	49	25	36.3	25	38.0	0.955	25	36.7	25	36.6	1.002	36.5	37.3	0.978	20	25.2
	50	25	44.5	25	46.5	0.957	25	43.8	25	43.5	1.007	44.1	45.0	0.982	20	43.9
	51	25	42.2	25	40.9	1.031	25	41.0	25	41.3	0.993	41.6	41.1	1.012	20	38.4
	52	25	44.7	25	43.3	1.033	25	43.3	25	43.6	0.993	44.0	43.4	1.013	20	40.9
	53	25	35.5	25	34.5	1.030	25	35.9	25	34.0	1.057	35.7	34.2	1.044	15	30.1
	54	25	39.6	25	35.8	1.106	25	38.5	25	37.4	1.028	39.1	36.6	1.067	20	35.5
	55	25	42.6	25	44.7	0.954	25	43.8	25	42.0	1.044	43.2	43.3	0.999	20	40.1
	56	25	39.7	25	40.4	0.983	25	37.3	25	39.0	0.957	38.5	39.7	0.970	20	34.6
	57	25	42.5	25	43.7	0.974	25	40.7	25	42.2	0.965	41.6	42.9	0.969	15	31.9
	58	25	46.8	25	46.1	1.015	25	44.6	25	45.2	0.986	45.7	45.7	1.001	20	42.0
	59	25	39.7	25	38.4	1.034	25	36.3	25	35.8	1.013	38.0	37.1	1.024	20	31.6
	61	25	34.6	25	34.9	0.990	25	33.9	25	34.2	0.992	34.2	34.5	0.991	20	27.9
	63	25	48.9	25	48.9	0.998	25	48.2	25	42.5	1.135	48.5	45.7	1.067	20	38.5
	64	25	36.4	25	35.1	1.036	25	35.3	25	33.7	1.047	35.8	34.4	1.042	20	32.0
	66	20	38.3	20	35.6	1.073	20	37.2	20	32.4	1.148	37.7	34.0	1.111	20	37.8

(to be continued)

Supplemental Table 7. (continued)

		Two-grained spikelets														One-grained spikelets	
		Replicate 1					Replicate 2					Averages				
		First florets		Second florets			First florets		Second florets			One-grain weight (mg)			First florets	
Line and Population No.	Plant No.	No. of grains measured	One-grain weight (mg)	No. of grains measured	One-grain weight (mg)	GWR	No. of grains measured	One-grain weight (mg)	No. of grains measured	One-grain weight (mg)	GWR	First florets	Second florets	GWR	No. of grains measured	One-grain weight (mg)
13406	67	25	37.7	25	39.0	0.969	25	38.0	25	38.4	0.989	37.9	38.7	0.979	20	33.9
	68	10	52.8	10	48.9	1.079	10	51.0	10	46.8	1.089	51.9	47.9	1.084	10	48.8
	69	15	30.0	15	46.6	0.644	15	27.1	15	45.2	0.601	28.6	45.9	0.622	20	43.3
	70	25	43.7	25	41.4	1.055	25	44.0	25	38.2	1.152	43.8	39.8	1.103	20	37.8
	71	20	54.3	20	54.0	1.006	20	50.5	20	50.0	1.011	52.4	52.0	1.008	20	52.8
	72	25	45.3	25	43.4	1.043	25	39.1	25	41.1	0.951	42.2	42.3	0.997	15	39.1
	74	10	52.9	10	51.5	1.026	10	48.0	10	51.4	0.934	50.4	51.5	0.980	20	50.7
	75	25	38.4	25	42.8	0.897	25	41.7	25	43.5	0.958	40.0	43.2	0.928	10	31.8
	76	25	41.8	25	43.4	0.965	25	43.4	25	45.9	0.945	42.6	44.6	0.955	20	44.1
	77	25	45.2	25	45.4	0.995	25	44.3	25	44.8	0.988	44.8	45.1	0.992	20	33.7
	78	25	41.0	25	38.0	1.079	25	39.1	25	38.1	1.028	40.1	38.0	1.053	15	30.3
	80	15	36.4	15	31.7	1.150	15	36.1	15	30.6	1.179	36.3	31.1	1.165	20	36.0
	81	25	37.1	25	36.4	1.020	25	35.9	25	35.5	1.013	36.5	35.9	1.016	20	30.6
	82	20	44.2	20	45.1	0.980	20	40.9	20	41.6	0.983	42.5	43.3	0.981	20	46.5
	83	20	35.4	20	35.4	1.001	20	37.0	20	32.7	1.131	36.2	34.0	1.066	20	37.6
	84	25	36.5	25	34.9	1.047	25	33.9	25	32.8	1.033	35.2	33.8	1.040	20	26.0
	85	25	36.7	25	35.2	1.041	25	34.0	25	38.4	0.885	35.3	36.8	0.963	20	33.6
	86	20	35.1	20	33.5	1.046	20	34.8	20	32.6	1.069	34.9	33.0	1.058	20	34.4
	87	20	41.1	20	37.3	1.103	20	40.6	20	38.3	1.060	40.9	37.8	1.081	20	40.0
	88	25	45.2	25	44.1	1.025	25	43.9	25	41.1	1.069	44.5	42.6	1.047	20	45.2

Supplemental Table 8. GR and GI values of the three grain groups in wild and domesticated parental lines, their reciprocal F_1 hybrids, and F_2 populations derived from self-pollination of the F_1 hybrids (sown on September 18, 2013)

Line and Population No.	Plant No.	Two-grained spikelets																	One-grained spikelets		
		Replicate 1						Replicate 2						Averages							
		First florets			Second florets			First florets			Second florets			First florets		Second florets			First florets		
		No. of grains sown	GR	GI	No. of grains sown	GR	GI	No. of grains sown	GR	GI	No. of grains sown	GR	GI	GR	GI	GR	GI		No. of grains sown	GR	GI
Wild emmer parent																					
W43	1	15	0.87	0.79	15	1.00	1.00	15	0.80	0.73	15	1.00	1.00	0.83	0.76	1.00	1.00		15	1.00	1.00
	2	20	0.55	0.49	20	1.00	1.00	20	0.60	0.56	20	1.00	1.00	0.58	0.52	1.00	1.00		20	1.00	1.00
	3	20	0.40	0.37	20	1.00	1.00	19	0.68	0.61	20	1.00	1.00	0.54	0.49	1.00	1.00		15	1.00	1.00
	6	20	0.25	0.22	20	1.00	1.00	21	0.52	0.48	20	1.00	1.00	0.39	0.35	1.00	1.00		15	1.00	1.00
Domesticated emmer parent																					
D26	1	25	1.00	1.00	25	1.00	1.00	25	1.00	1.00	25	1.00	1.00	1.00	1.00	1.00	1.00		10	1.00	1.00
	2	25	1.00	1.00	26	1.00	1.00	25	1.00	1.00	25	1.00	1.00	1.00	1.00	1.00	1.00		20	1.00	1.00
	8	25	1.00	1.00	25	1.00	1.00	25	1.00	1.00	25	1.00	1.00	1.00	1.00	1.00	1.00		20	1.00	1.00
	9	25	1.00	1.00	25	1.00	1.00	24	1.00	1.00	25	1.00	1.00	1.00	1.00	1.00	1.00		10	1.00	1.00
F_1 hybrids obtained from the reciprocal crosses between wild (W43) and domesticated (D26) emmer parents																					
W43 x D26	1	25	1.00	1.00	25	1.00	1.00	25	1.00	1.00	24	1.00	1.00	1.00	1.00	1.00	1.00		15	1.00	1.00
	2	25	1.00	1.00	25	1.00	1.00	25	1.00	1.00	25	1.00	1.00	1.00	1.00	1.00	1.00		20	1.00	1.00
	4	25	1.00	1.00	25	1.00	1.00	25	1.00	1.00	25	1.00	1.00	1.00	1.00	1.00	1.00		20	1.00	1.00
	5	25	1.00	1.00	25	1.00	1.00	25	1.00	1.00	25	1.00	1.00	1.00	1.00	1.00	1.00		20	1.00	1.00
D26 x W43	1	25	1.00	1.00	25	1.00	1.00	25	1.00	1.00	25	1.00	1.00	1.00	1.00	1.00	1.00		15	1.00	1.00
	2	25	1.00	1.00	25	1.00	1.00	25	1.00	1.00	25	1.00	1.00	1.00	1.00	1.00	1.00		20	1.00	1.00
	3	20	1.00	1.00	20	1.00	1.00	20	1.00	1.00	20	1.00	1.00	1.00	1.00	1.00	1.00		20	1.00	1.00
	4	20	1.00	1.00	20	1.00	1.00	20	1.00	1.00	20	1.00	1.00	1.00	1.00	1.00	1.00		15	1.00	1.00
F_2 population derived from the cross combination of wild emmer parent (W43) x domesticated emmer parent (D26)																					
13405	1	25	1.00	1.00	25	1.00	1.00	25	1.00	1.00	25	1.00	1.00	1.00	1.00	1.00	1.00		20	1.00	1.00
	2	25	1.00	1.00	25	1.00	1.00	25	1.00	1.00	25	1.00	1.00	1.00	1.00	1.00	1.00		20	1.00	1.00
	3	15	1.00	1.00	15	1.00	1.00	15	1.00	1.00	15	1.00	1.00	1.00	1.00	1.00	1.00		20	1.00	1.00
	5	25	0.96	0.96	25	1.00	1.00	25	1.00	1.00	25	1.00	1.00	0.98	0.98	1.00	1.00		15	1.00	1.00
	8	25	1.00	1.00	25	1.00	1.00	25	1.00	1.00	25	1.00	1.00	1.00	1.00	1.00	1.00		20	1.00	1.00
	9	25	1.00	1.00	25	1.00	1.00	25	1.00	1.00	25	1.00	1.00	1.00	1.00	1.00	1.00		15	1.00	1.00
	10	25	1.00	1.00	25	1.00	1.00	25	1.00	1.00	25	1.00	1.00	1.00	1.00	1.00	1.00		20	1.00	1.00
	11	25	1.00	1.00	25	1.00	1.00	25	1.00	1.00	25	1.00	1.00	1.00	1.00	1.00	1.00		10	1.00	1.00
	12	25	1.00	1.00	25	1.00	1.00	25	1.00	1.00	25	1.00	1.00	1.00	1.00	1.00	1.00		20	1.00	1.00
	13	15	1.00	1.00	15	1.00	1.00	15	1.00	1.00	15	1.00	1.00	1.00	1.00	1.00	1.00		15	1.00	1.00

(to be continued)

Supplemental Table 8. (continued)

Line and Population No.	Plant No.	Two-grained spikelets																One-grained spikelets		
		Replicate 1						Replicate 2						Averages						
		First florets			Second florets			First florets			Second florets			First florets		Second florets		First florets		
		No. of grains sown	GR	GI	No. of grains sown	GR	GI	No. of grains sown	GR	GI	No. of grains sown	GR	GI	GR	GI	GR	GI	No. of grains sown	GR	GI
13405	14	25	1.00	1.00	25	1.00	1.00	25	1.00	1.00	25	1.00	1.00	1.00	1.00	1.00	1.00	20	1.00	1.00
	15	25	1.00	1.00	25	1.00	1.00	25	1.00	1.00	25	1.00	1.00	1.00	1.00	1.00	1.00	15	1.00	1.00
	16	25	1.00	1.00	25	1.00	1.00	15	1.00	1.00	15	1.00	1.00	1.00	1.00	1.00	1.00	15	1.00	1.00
	17	25	1.00	1.00	25	1.00	1.00	25	1.00	1.00	25	1.00	1.00	1.00	1.00	1.00	1.00	10	1.00	1.00
	18	25	1.00	1.00	25	1.00	1.00	25	1.00	1.00	25	1.00	1.00	1.00	1.00	1.00	1.00	20	1.00	1.00
	19	25	1.00	1.00	25	1.00	1.00	25	1.00	1.00	25	1.00	1.00	1.00	1.00	1.00	1.00	10	1.00	1.00
	20	25	1.00	1.00	25	1.00	1.00	25	1.00	1.00	25	1.00	1.00	1.00	1.00	1.00	1.00	20	1.00	1.00
	22	10	1.00	1.00	10	1.00	1.00	10	1.00	1.00	10	1.00	1.00	1.00	1.00	1.00	1.00	15	1.00	1.00
	24	25	1.00	1.00	25	1.00	1.00	25	1.00	1.00	25	1.00	1.00	1.00	1.00	1.00	1.00	5	1.00	1.00
	25	25	1.00	1.00	25	1.00	1.00	24	1.00	1.00	25	1.00	1.00	1.00	1.00	1.00	1.00	10	1.00	1.00
	26	25	1.00	1.00	25	1.00	1.00	25	1.00	1.00	25	1.00	1.00	1.00	1.00	1.00	1.00	15	1.00	1.00
	27	25	1.00	1.00	25	1.00	1.00	25	1.00	1.00	24	1.00	1.00	1.00	1.00	1.00	1.00	15	1.00	1.00
	28	15	1.00	1.00	15	1.00	1.00	15	1.00	1.00	15	1.00	1.00	1.00	1.00	1.00	1.00	15	1.00	1.00
	29	15	1.00	1.00	15	1.00	1.00	15	1.00	1.00	15	1.00	1.00	1.00	1.00	1.00	1.00	15	1.00	1.00
	30	25	1.00	1.00	25	1.00	1.00	25	1.00	1.00	25	1.00	1.00	1.00	1.00	1.00	1.00	20	1.00	1.00
	31	25	1.00	1.00	25	1.00	1.00	25	1.00	1.00	25	1.00	1.00	1.00	1.00	1.00	1.00	10	1.00	1.00
	33	25	1.00	1.00	25	1.00	1.00	25	1.00	1.00	25	1.00	1.00	1.00	1.00	1.00	1.00	10	1.00	1.00
	34	25	1.00	1.00	25	1.00	1.00	25	1.00	1.00	25	1.00	1.00	1.00	1.00	1.00	1.00	20	1.00	1.00
	35	25	1.00	1.00	25	1.00	1.00	25	1.00	1.00	25	1.00	1.00	1.00	1.00	1.00	1.00	20	1.00	1.00
	36	25	1.00	1.00	25	1.00	1.00	25	1.00	1.00	25	1.00	1.00	1.00	1.00	1.00	1.00	20	1.00	1.00
	37	25	1.00	1.00	25	1.00	1.00	25	1.00	1.00	25	1.00	1.00	1.00	1.00	1.00	1.00	20	1.00	1.00
	38	25	1.00	1.00	25	1.00	1.00	25	1.00	1.00	25	1.00	1.00	1.00	1.00	1.00	1.00	20	1.00	1.00
	39	25	1.00	1.00	25	1.00	1.00	25	1.00	1.00	25	1.00	1.00	1.00	1.00	1.00	1.00	20	1.00	1.00
	40	25	1.00	1.00	25	1.00	1.00	25	1.00	1.00	25	1.00	1.00	1.00	1.00	1.00	1.00	15	1.00	1.00
	41	25	1.00	1.00	25	1.00	1.00	25	1.00	1.00	25	1.00	1.00	1.00	1.00	1.00	1.00	15	1.00	1.00
	43	25	1.00	1.00	25	1.00	1.00	25	1.00	1.00	25	1.00	1.00	1.00	1.00	1.00	1.00	5	1.00	1.00
	44	25	1.00	1.00	25	1.00	1.00	25	1.00	1.00	25	1.00	1.00	1.00	1.00	1.00	1.00	20	1.00	1.00
	45	25	1.00	1.00	25	1.00	1.00	25	1.00	1.00	25	1.00	1.00	1.00	1.00	1.00	1.00	15	1.00	1.00
	46	25	0.88	0.86	25	1.00	1.00	25	1.00	1.00	25	1.00	1.00	0.94	0.93	1.00	1.00	10	1.00	1.00
	47	25	0.84	0.82	25	1.00	1.00	25	0.92	0.90	25	1.00	1.00	0.88	0.86	1.00	1.00	20	1.00	1.00

(to be continued)

Supplemental Table 8. (continued)

Line and Population No.	Plant No.	Two-grained spikelets																One-grained spikelets		
		Replicate 1						Replicate 2						Averages						
		First florets			Second florets			First florets			Second florets			First florets		Second florets		First florets		
		No. of grains sown	GR	GI	No. of grains sown	GR	GI	No. of grains sown	GR	GI	No. of grains sown	GR	GI	GR	GI	GR	GI	No. of grains sown	GR	GI
13405	48	25	1.00	1.00	25	1.00	1.00	25	1.00	1.00	25	1.00	1.00	1.00	1.00	1.00	1.00	20	1.00	1.00
	49	25	1.00	1.00	25	1.00	1.00	25	1.00	1.00	25	1.00	1.00	1.00	1.00	1.00	1.00	5	1.00	1.00
	50	25	1.00	0.98	25	1.00	1.00	25	1.00	1.00	25	1.00	1.00	1.00	0.99	1.00	1.00	10	1.00	1.00
	51	25	1.00	1.00	25	1.00	1.00	25	1.00	1.00	25	1.00	1.00	1.00	1.00	1.00	1.00	20	1.00	1.00
	52	25	0.92	0.92	25	1.00	1.00	25	0.84	0.83	25	1.00	1.00	0.88	0.87	1.00	1.00	20	1.00	1.00
	53	25	1.00	1.00	25	1.00	1.00	25	1.00	1.00	25	1.00	1.00	1.00	1.00	1.00	1.00	15	1.00	1.00
	54	25	1.00	1.00	25	1.00	1.00	25	1.00	1.00	25	1.00	1.00	1.00	1.00	1.00	1.00	15	1.00	1.00
	55	25	1.00	1.00	25	1.00	1.00	25	1.00	1.00	25	1.00	1.00	1.00	1.00	1.00	1.00	20	1.00	1.00
	56	15	1.00	1.00	15	1.00	1.00	15	1.00	1.00	15	1.00	1.00	1.00	1.00	1.00	1.00	20	1.00	1.00
	57	25	1.00	1.00	25	1.00	1.00	25	1.00	0.98	25	1.00	1.00	1.00	0.99	1.00	1.00	20	1.00	1.00
	58	25	1.00	1.00	25	1.00	1.00	25	1.00	1.00	25	1.00	1.00	1.00	1.00	1.00	1.00	20	1.00	1.00
	59	25	1.00	1.00	25	1.00	1.00	25	1.00	1.00	25	1.00	1.00	1.00	1.00	1.00	1.00	20	1.00	1.00
	61	25	1.00	1.00	25	1.00	1.00	25	1.00	1.00	25	1.00	1.00	1.00	1.00	1.00	1.00	20	1.00	1.00
	62	25	1.00	1.00	25	1.00	1.00	25	1.00	1.00	25	1.00	1.00	1.00	1.00	1.00	1.00	20	1.00	1.00
	63	25	1.00	1.00	25	1.00	1.00	25	1.00	1.00	25	1.00	1.00	1.00	1.00	1.00	1.00	20	1.00	1.00
	64	25	1.00	1.00	25	1.00	1.00	25	1.00	1.00	25	1.00	1.00	1.00	1.00	1.00	1.00	10	1.00	1.00
	66	25	1.00	1.00	25	1.00	1.00	25	1.00	1.00	25	1.00	1.00	1.00	1.00	1.00	1.00	20	1.00	1.00
	67	25	1.00	1.00	25	1.00	1.00	25	1.00	1.00	25	1.00	1.00	1.00	1.00	1.00	1.00	20	1.00	1.00
	69	15	1.00	1.00	15	1.00	1.00	15	1.00	1.00	15	1.00	1.00	1.00	1.00	1.00	1.00	15	1.00	1.00
	70	15	1.00	1.00	15	1.00	1.00	15	1.00	1.00	15	1.00	1.00	1.00	1.00	1.00	1.00	15	1.00	1.00
	71	25	1.00	1.00	25	1.00	1.00	25	1.00	1.00	25	1.00	1.00	1.00	1.00	1.00	1.00	20	1.00	1.00
	72	25	1.00	1.00	25	1.00	1.00	25	1.00	1.00	25	1.00	1.00	1.00	1.00	1.00	1.00	20	1.00	1.00
	73	25	1.00	1.00	25	1.00	1.00	25	1.00	1.00	25	1.00	1.00	1.00	1.00	1.00	1.00	20	1.00	1.00
	74	25	1.00	1.00	25	1.00	1.00	25	1.00	1.00	25	1.00	1.00	1.00	1.00	1.00	1.00	20	1.00	1.00
	75	25	1.00	1.00	25	1.00	1.00	25	1.00	1.00	25	1.00	1.00	1.00	1.00	1.00	1.00	20	1.00	1.00
	76	25	1.00	1.00	25	1.00	1.00	25	1.00	1.00	25	1.00	1.00	1.00	1.00	1.00	1.00	20	1.00	1.00
	77	25	1.00	1.00	25	1.00	1.00	25	1.00	1.00	25	1.00	1.00	1.00	1.00	1.00	1.00	20	1.00	1.00
	78	25	1.00	1.00	25	1.00	1.00	25	1.00	1.00	25	1.00	1.00	1.00	1.00	1.00	1.00	20	1.00	1.00
	79	25	1.00	1.00	25	1.00	1.00	25	1.00	1.00	25	1.00	1.00	1.00	1.00	1.00	1.00	15	1.00	1.00
	80	25	1.00	1.00	25	1.00	1.00	25	1.00	1.00	25	1.00	1.00	1.00	1.00	1.00	1.00	20	1.00	1.00

(to be continued)

Supplemental Table 8. (continued)

Line and Population No.	Plant No.	Two-grained spikelets																One-grained spikelets		
		Replicate 1						Replicate 2						Averages						
		First florets			Second florets			First florets			Second florets			First florets		Second florets		First florets		
		No. of grains sown	GR	GI	No. of grains sown	GR	GI	No. of grains sown	GR	GI	No. of grains sown	GR	GI	GR	GI	GR	GI	No. of grains sown	GR	GI
13405	81	25	1.00	1.00	25	1.00	1.00	25	1.00	1.00	25	1.00	1.00	1.00	1.00	1.00	1.00	20	1.00	1.00
	82	25	1.00	1.00	25	1.00	1.00	25	1.00	1.00	25	1.00	1.00	1.00	1.00	1.00	1.00	15	1.00	1.00
	83	25	1.00	1.00	25	1.00	1.00	25	1.00	1.00	25	1.00	1.00	1.00	1.00	1.00	1.00	20	1.00	1.00
	84	25	1.00	1.00	25	1.00	1.00	25	1.00	1.00	25	1.00	1.00	1.00	1.00	1.00	1.00	20	1.00	1.00
	85	25	1.00	1.00	25	1.00	1.00	25	1.00	1.00	25	1.00	1.00	1.00	1.00	1.00	1.00	20	1.00	1.00
	86	25	1.00	1.00	25	1.00	1.00	25	1.00	1.00	25	1.00	1.00	1.00	1.00	1.00	1.00	20	1.00	1.00
	87	20	1.00	1.00	20	1.00	1.00	20	1.00	1.00	20	1.00	1.00	1.00	1.00	1.00	1.00	20	1.00	1.00
	89	25	1.00	1.00	25	1.00	1.00	25	1.00	1.00	25	1.00	1.00	1.00	1.00	1.00	1.00	20	1.00	1.00
	90	25	1.00	1.00	25	1.00	1.00	25	1.00	1.00	25	1.00	1.00	1.00	1.00	1.00	1.00	20	1.00	1.00
	91	20	1.00	1.00	20	1.00	1.00	20	1.00	1.00	20	1.00	1.00	1.00	1.00	1.00	1.00	20	1.00	1.00
	92	25	1.00	1.00	25	1.00	1.00	25	1.00	1.00	25	1.00	1.00	1.00	1.00	1.00	1.00	15	1.00	1.00
	93	25	1.00	1.00	25	1.00	1.00	25	1.00	1.00	25	1.00	1.00	1.00	1.00	1.00	1.00	20	1.00	1.00
	94	25	0.96	0.95	25	1.00	1.00	25	0.96	0.96	25	1.00	1.00	0.96	0.95	1.00	1.00	15	1.00	1.00
	95	25	1.00	1.00	25	1.00	1.00	25	1.00	1.00	24	1.00	1.00	1.00	1.00	1.00	1.00	20	1.00	1.00
F_2 population derived from the cross combination of domesticated emmer parent (D26) x wild emmer parent (W43)																				
13406	1	25	1.00	1.00	25	1.00	1.00	25	1.00	1.00	25	1.00	1.00	1.00	1.00	1.00	1.00	20	1.00	1.00
	2	25	1.00	0.99	25	1.00	1.00	25	1.00	1.00	25	1.00	1.00	1.00	1.00	1.00	1.00	20	1.00	1.00
	3	25	1.00	1.00	25	1.00	1.00	25	1.00	1.00	25	1.00	1.00	1.00	1.00	1.00	1.00	20	1.00	1.00
	5	25	1.00	1.00	25	1.00	1.00	25	1.00	1.00	25	1.00	1.00	1.00	1.00	1.00	1.00	20	1.00	1.00
	6	25	1.00	1.00	25	1.00	1.00	25	1.00	1.00	25	1.00	1.00	1.00	1.00	1.00	1.00	20	1.00	1.00
	7	25	1.00	1.00	25	1.00	1.00	25	1.00	1.00	25	1.00	1.00	1.00	1.00	1.00	1.00	20	1.00	1.00
	8	25	1.00	1.00	25	1.00	1.00	25	1.00	1.00	25	1.00	1.00	1.00	1.00	1.00	1.00	20	1.00	1.00
	9	25	1.00	1.00	25	1.00	1.00	25	1.00	1.00	25	1.00	1.00	1.00	1.00	1.00	1.00	20	1.00	1.00
	10	25	1.00	1.00	25	1.00	1.00	25	1.00	1.00	25	1.00	1.00	1.00	1.00	1.00	1.00	15	1.00	1.00
	11	25	1.00	1.00	25	1.00	1.00	25	1.00	1.00	25	1.00	1.00	1.00	1.00	1.00	1.00	20	1.00	1.00
	13	25	1.00	1.00	25	1.00	1.00	25	1.00	1.00	25	1.00	1.00	1.00	1.00	1.00	1.00	15	1.00	1.00
	14	15	1.00	1.00	15	1.00	1.00	15	1.00	1.00	15	1.00	1.00	1.00	1.00	1.00	1.00	20	1.00	1.00
	15	25	1.00	1.00	25	1.00	1.00	25	1.00	1.00	25	1.00	1.00	1.00	1.00	1.00	1.00	20	1.00	1.00
	16	25	1.00	1.00	25	1.00	1.00	25	1.00	1.00	25	1.00	1.00	1.00	1.00	1.00	1.00	15	1.00	1.00
	17	25	1.00	1.00	25	1.00	1.00	25	1.00	1.00	25	1.00	1.00	1.00	1.00	1.00	1.00	20	1.00	1.00

(to be continued)

Supplemental Table 8. (continued)

Line and Population No.	Plant No.	Two-grained spikelets																One-grained spikelets		
		Replicate 1						Replicate 2						Averages						
		First florets			Second florets			First florets			Second florets			First florets		Second florets		First florets		
		No. of grains sown	GR	GI	No. of grains sown	GR	GI	No. of grains sown	GR	GI	No. of grains sown	GR	GI	GR	GI	GR	GI	No. of grains sown	GR	GI
13406	18	25	0.92	0.91	25	1.00	1.00	25	1.00	1.00	25	1.00	1.00	0.96	0.96	1.00	1.00	10	1.00	0.99
	19	25	1.00	1.00	25	1.00	1.00	20	1.00	1.00	20	1.00	1.00	1.00	1.00	1.00	1.00	20	1.00	1.00
	20	15	1.00	1.00	15	1.00	1.00	15	1.00	1.00	15	1.00	1.00	1.00	1.00	1.00	1.00	20	1.00	1.00
	21	20	1.00	1.00	20	1.00	1.00	20	1.00	1.00	20	1.00	1.00	1.00	1.00	1.00	1.00	20	1.00	1.00
	22	20	1.00	1.00	20	1.00	1.00	20	1.00	1.00	20	1.00	1.00	1.00	1.00	1.00	1.00	20	1.00	1.00
	23	20	1.00	1.00	20	1.00	1.00	20	1.00	1.00	20	1.00	1.00	1.00	1.00	1.00	1.00	20	1.00	1.00
	24	25	1.00	1.00	25	1.00	1.00	25	1.00	1.00	25	1.00	1.00	1.00	1.00	1.00	1.00	15	1.00	1.00
	26	15	1.00	1.00	15	1.00	1.00	15	1.00	1.00	15	1.00	1.00	1.00	1.00	1.00	1.00	20	1.00	1.00
	27	25	1.00	1.00	25	1.00	1.00	25	1.00	1.00	25	1.00	1.00	1.00	1.00	1.00	1.00	20	1.00	1.00
	28	25	1.00	1.00	25	1.00	1.00	20	1.00	1.00	20	1.00	1.00	1.00	1.00	1.00	1.00	20	1.00	1.00
	29	25	1.00	1.00	25	1.00	1.00	25	1.00	1.00	23	1.00	1.00	1.00	1.00	1.00	1.00	20	1.00	1.00
	30	25	1.00	1.00	25	1.00	1.00	25	1.00	1.00	25	1.00	1.00	1.00	1.00	1.00	1.00	20	1.00	1.00
	31	15	1.00	1.00	15	1.00	1.00	15	1.00	1.00	15	1.00	1.00	1.00	1.00	1.00	1.00	20	1.00	1.00
	32	25	1.00	1.00	25	1.00	1.00	25	1.00	1.00	25	1.00	1.00	1.00	1.00	1.00	1.00	20	1.00	1.00
	33	25	1.00	1.00	25	1.00	1.00	25	1.00	1.00	25	1.00	1.00	1.00	1.00	1.00	1.00	15	1.00	1.00
	34	25	1.00	1.00	25	1.00	1.00	25	1.00	1.00	25	1.00	1.00	1.00	1.00	1.00	1.00	10	1.00	1.00
	35	25	1.00	1.00	25	1.00	1.00	25	1.00	1.00	25	1.00	1.00	1.00	1.00	1.00	1.00	20	1.00	1.00
	36	25	1.00	1.00	25	1.00	1.00	25	1.00	1.00	25	1.00	1.00	1.00	1.00	1.00	1.00	20	1.00	1.00
	37	25	1.00	1.00	25	1.00	1.00	25	1.00	1.00	25	1.00	1.00	1.00	1.00	1.00	1.00	20	1.00	1.00
	38	25	1.00	1.00	25	1.00	1.00	25	1.00	1.00	25	1.00	1.00	1.00	1.00	1.00	1.00	15	1.00	1.00
	39	25	1.00	1.00	25	1.00	1.00	25	1.00	1.00	25	1.00	1.00	1.00	1.00	1.00	1.00	20	1.00	1.00
	40	25	0.92	0.87	25	1.00	1.00	25	1.00	1.00	25	1.00	1.00	0.96	0.93	1.00	1.00	20	1.00	1.00
	41	25	1.00	1.00	25	1.00	1.00	25	1.00	1.00	25	1.00	1.00	1.00	1.00	1.00	1.00	10	1.00	1.00
	42	25	1.00	1.00	25	1.00	1.00	25	1.00	1.00	25	1.00	1.00	1.00	1.00	1.00	1.00	10	1.00	1.00
	43	15	1.00	1.00	15	1.00	1.00	15	1.00	1.00	15	1.00	1.00	1.00	1.00	1.00	1.00	20	1.00	1.00
	44	25	1.00	1.00	25	1.00	1.00	25	1.00	1.00	25	1.00	1.00	1.00	1.00	1.00	1.00	20	1.00	1.00
	47	25	1.00	1.00	25	1.00	1.00	25	1.00	1.00	25	1.00	1.00	1.00	1.00	1.00	1.00	20	1.00	1.00
	48	25	1.00	1.00	25	1.00	1.00	25	1.00	1.00	25	1.00	1.00	1.00	1.00	1.00	1.00	20	1.00	1.00
	49	25	1.00	1.00	25	1.00	1.00	25	1.00	1.00	25	1.00	1.00	1.00	1.00	1.00	1.00	20	1.00	1.00
	50	25	1.00	1.00	25	1.00	1.00	25	1.00	1.00	25	1.00	1.00	1.00	1.00	1.00	1.00	20	1.00	1.00
	51	25	1.00	1.00	25	1.00	1.00	25	1.00	1.00	25	1.00	1.00	1.00	1.00	1.00	1.00	20	1.00	1.00

(to be continued)

Supplemental Table 8. (continued)

Line and Population No.	Plant No.	Two-grained spikelets																	One-grained spikelets		
		Replicate 1						Replicate 2						Averages							
		First florets			Second florets			First florets			Second florets			First florets		Second florets			First florets		
		No. of grains sown	GR	GI	No. of grains sown	GR	GI	No. of grains sown	GR	GI	No. of grains sown	GR	GI	GR	GI	GR	GI		No. of grains sown	GR	GI
13406	52	25	1.00	1.00	25	1.00	1.00	25	1.00	1.00	25	1.00	1.00	1.00	1.00	1.00	1.00		20	1.00	1.00
	53	25	1.00	1.00	25	1.00	1.00	25	1.00	1.00	25	1.00	1.00	1.00	1.00	1.00	1.00		15	1.00	1.00
	54	25	1.00	1.00	25	1.00	1.00	25	1.00	1.00	25	1.00	1.00	1.00	1.00	1.00	1.00		20	1.00	1.00
	55	25	1.00	1.00	25	1.00	1.00	25	1.00	1.00	25	1.00	1.00	1.00	1.00	1.00	1.00		20	1.00	1.00
	56	25	1.00	1.00	25	1.00	1.00	25	1.00	1.00	25	1.00	1.00	1.00	1.00	1.00	1.00		20	1.00	1.00
	57	25	1.00	1.00	25	1.00	1.00	25	1.00	1.00	25	1.00	1.00	1.00	1.00	1.00	1.00		15	1.00	1.00
	58	25	1.00	1.00	25	1.00	1.00	25	1.00	1.00	25	1.00	1.00	1.00	1.00	1.00	1.00		20	1.00	1.00
	59	25	1.00	1.00	25	1.00	1.00	25	1.00	1.00	25	1.00	1.00	1.00	1.00	1.00	1.00		20	1.00	1.00
	61	25	1.00	1.00	25	1.00	1.00	25	1.00	1.00	25	1.00	1.00	1.00	1.00	1.00	1.00		20	1.00	1.00
	63	25	1.00	1.00	25	1.00	1.00	25	1.00	1.00	25	1.00	1.00	1.00	1.00	1.00	1.00		20	1.00	1.00
	64	25	1.00	1.00	25	1.00	1.00	25	1.00	1.00	25	1.00	1.00	1.00	1.00	1.00	1.00		20	1.00	1.00
	66	20	1.00	1.00	20	1.00	1.00	20	1.00	1.00	20	1.00	1.00	1.00	1.00	1.00	1.00		20	1.00	1.00
	67	25	1.00	1.00	25	1.00	1.00	25	1.00	1.00	25	1.00	1.00	1.00	1.00	1.00	1.00		20	1.00	1.00
	68	10	1.00	1.00	10	1.00	1.00	10	1.00	1.00	10	1.00	1.00	1.00	1.00	1.00	1.00		10	1.00	1.00
	69	15	1.00	1.00	15	1.00	1.00	15	1.00	1.00	15	1.00	1.00	1.00	1.00	1.00	1.00		20	1.00	1.00
	70	25	1.00	1.00	25	1.00	1.00	25	1.00	1.00	25	1.00	1.00	1.00	1.00	1.00	1.00		20	1.00	1.00
	71	25	1.00	1.00	25	1.00	1.00	20	1.00	1.00	20	1.00	1.00	1.00	1.00	1.00	1.00		20	1.00	1.00
	72	25	1.00	1.00	25	1.00	1.00	25	1.00	1.00	25	1.00	1.00	1.00	1.00	1.00	1.00		15	1.00	1.00
	74	10	1.00	1.00	10	1.00	1.00	10	1.00	1.00	10	1.00	1.00	1.00	1.00	1.00	1.00		20	1.00	1.00
	75	25	1.00	1.00	25	1.00	1.00	25	1.00	1.00	25	1.00	1.00	1.00	1.00	1.00	1.00		10	1.00	1.00
	76	25	1.00	1.00	25	1.00	1.00	25	1.00	1.00	25	1.00	1.00	1.00	1.00	1.00	1.00		20	1.00	1.00
	77	25	1.00	1.00	25	1.00	1.00	25	1.00	1.00	25	1.00	1.00	1.00	1.00	1.00	1.00		20	1.00	1.00
	78	25	1.00	1.00	25	1.00	1.00	25	1.00	1.00	25	1.00	1.00	1.00	1.00	1.00	1.00		15	1.00	1.00
	80	15	1.00	1.00	15	1.00	1.00	15	1.00	1.00	15	1.00	1.00	1.00	1.00	1.00	1.00		20	1.00	1.00
	81	25	1.00	1.00	25	1.00	1.00	25	1.00	1.00	25	1.00	1.00	1.00	1.00	1.00	1.00		20	1.00	1.00
	82	20	1.00	1.00	20	1.00	1.00	20	1.00	1.00	20	1.00	1.00	1.00	1.00	1.00	1.00		20	1.00	1.00
	83	20	1.00	1.00	20	1.00	1.00	20	1.00	1.00	20	1.00	1.00	1.00	1.00	1.00	1.00		20	1.00	1.00
	84	25	1.00	1.00	25	1.00	1.00	25	1.00	1.00	25	1.00	1.00	1.00	1.00	1.00	1.00		20	1.00	1.00
	85	25	1.00	1.00	24	1.00	1.00	25	1.00	1.00	25	1.00	1.00	1.00	1.00	1.00	1.00		20	1.00	1.00
	86	20	1.00	1.00	20	1.00	1.00	20	1.00	1.00	20	1.00	1.00	1.00	1.00	1.00	1.00		20	1.00	1.00
	87	20	1.00	1.00	20	1.00	1.00	20	1.00	1.00	20	1.00	1.00	1.00	1.00	1.00	1.00		20	1.00	1.00
	88	25	1.00	1.00	25	1.00	1.00	25	1.00	1.00	25	1.00	1.00	1.00	1.00	1.00	1.00		20	1.00	1.00

Supplemental Table 9. One-grain weights, GWR, GR, and GI values of the first and second floret grains in two-grained spikelets of the F_3 population derived from self-pollination of the F_2 plant 12403A-24 (sown on October 17, 2015)

		First florets					Second florets					
Population No.	Plant No.	No. of grains measured	One-grain weight (mg)	No. of grains sown	GR	GI	No. of grains measured	One-grain weight (mg)	No. of grains sown	GR	GI	GWR
15502	1	25	21.50	25	1.00	0.98	25	39.98	25	1.00	1.00	0.538
	2	25	14.82	25	1.00	0.99	25	28.43	25	1.00	1.00	0.521
	3	25	15.70	25	0.20	0.17	25	36.96	25	1.00	1.00	0.425
	4	25	16.40	25	0.24	0.20	25	33.68	25	1.00	1.00	0.487
	5	20	14.09	20	0.25	0.21	20	24.18	20	1.00	1.00	0.583
	6	25	18.92	25	0.84	0.75	25	38.17	25	1.00	1.00	0.496
	7	25	17.99	25	0.64	0.57	25	37.02	25	1.00	1.00	0.486
	8	25	23.09	25	1.00	0.99	25	41.80	25	1.00	1.00	0.552
	9	25	23.41	25	0.36	0.31	25	44.66	25	1.00	1.00	0.524
	10	25	19.20	25	0.32	0.28	25	33.42	25	1.00	1.00	0.575
	11	25	16.62	25	0.04	0.03	25	34.32	25	1.00	1.00	0.484
	12	25	20.19	25	0.12	0.10	25	37.75	25	1.00	1.00	0.535
	13	25	21.79	25	1.00	1.00	25	41.38	25	1.00	1.00	0.527
	14	25	18.02	25	0.00	0.00	25	36.49	25	1.00	1.00	0.494
	15	25	21.35	25	0.12	0.10	25	39.08	25	1.00	1.00	0.546
	16	25	22.78	25	0.68	0.59	25	42.85	25	1.00	1.00	0.532
	17	25	21.55	25	0.52	0.46	25	43.22	25	1.00	1.00	0.499
	18	20	15.94	20	0.90	0.87	20	34.29	20	1.00	1.00	0.465
	19	25	22.83	25	0.20	0.17	25	44.10	25	1.00	1.00	0.518
	20	15	17.73	14	0.21	0.19	15	33.25	15	1.00	1.00	0.533
	21	25	19.31	25	0.52	0.47	25	36.84	25	1.00	1.00	0.524
	22	25	13.16	25	0.12	0.10	25	26.04	25	1.00	1.00	0.505
	23	25	19.24	25	0.32	0.29	25	34.80	25	1.00	1.00	0.553
	24	25	18.35	25	0.44	0.39	25	35.67	25	1.00	1.00	0.514
	25	25	19.66	25	1.00	1.00	25	35.91	25	1.00	1.00	0.547

Supplemental Table 10. One-grain weights, GWR, GR, and GI values of the first and second floret grains in two-grained spikelets of the F_4 populations derived from the self-pollination of four low-GWR F_3 plants showing different GR and GI values (sown on September 4, 2016)

Population No.	Plant No.	First florets: No. of grains measured	First florets: One-grain weight (mg)	First florets: No. of grains sown	First florets: GR	First florets: GI	Second florets: No. of grains measured	Second florets: One-grain weight (mg)	Second florets: No. of grains sown	Second florets: GR	Second florets: GI	GWR
F_4 population derived from self-pollination of the F_3 plant 15502-14												
F_4-1a	1	25	24.18	25	0.24	0.18	25	44.58	25	1.00	1.00	0.542
	2	25	20.09	25	0.24	0.19	25	36.73	25	1.00	1.00	0.547
	5	25	19.00	25	0.28	0.24	25	39.20	25	1.00	1.00	0.485
	6	25	25.07	25	0.08	0.07	25	45.16	25	1.00	1.00	0.555
F_4-1b	3	25	24.06	25	0.56	0.48	25	38.97	25	1.00	1.00	0.617
	4	25	20.71	25	0.76	0.65	25	40.50	25	1.00	1.00	0.511
	7	25	22.16	25	0.84	0.73	25	42.41	25	1.00	1.00	0.523
	8	25	19.85	25	0.76	0.66	25	33.85	25	1.00	1.00	0.586
	9	25	20.84	25	0.56	0.45	25	35.82	25	1.00	1.00	0.582
F_4 population derived from self-pollination of the F_3 plant 15502-22												
F_4-2	1	25	16.49	25	0.56	0.50	25	31.74	25	1.00	1.00	0.520
	2	25	16.83	25	0.52	0.46	25	32.78	25	1.00	1.00	0.513
	3	25	16.97	25	0.52	0.47	25	34.16	25	1.00	1.00	0.497
	4	25	13.05	25	0.72	0.63	25	26.04	25	1.00	1.00	0.501
	5	25	16.80	25	0.64	0.55	25	33.47	25	1.00	1.00	0.502
	6	25	15.19	25	0.76	0.75	15	19.44	15	1.00	1.00	0.781
	7	25	16.74	25	0.68	0.59	25	34.20	25	1.00	1.00	0.490
	8	25	16.10	25	0.68	0.59	25	31.30	25	1.00	1.00	0.514
	9	25	16.87	25	0.60	0.53	25	29.66	25	1.00	1.00	0.569
	10	25	15.50	25	0.60	0.51	25	31.87	25	1.00	1.00	0.486
F_4 population derived from self-pollination of the F_3 plant 15502-17												
F_4-3	1	25	23.39	25	1.00	0.95	25	45.92	25	1.00	1.00	0.509
	2	25	21.42	25	1.00	0.95	25	41.84	25	1.00	1.00	0.512
	3	25	17.46	25	0.72	0.61	25	34.08	25	1.00	1.00	0.512
	4	25	20.39	25	0.96	0.82	25	39.74	25	1.00	1.00	0.513
	5	25	21.14	25	0.96	0.84	25	40.92	25	1.00	1.00	0.517
	6	25	19.76	25	0.92	0.82	25	37.32	25	1.00	1.00	0.529
	7	25	24.72	25	1.00	0.98	25	47.44	25	1.00	1.00	0.521
	8	25	24.35	25	1.00	0.98	25	43.43	25	1.00	1.00	0.561
	9	25	27.80	25	0.96	0.87	25	41.90	24	1.00	1.00	0.663
	10	25	23.26	25	0.76	0.67	25	44.28	25	1.00	1.00	0.525
	11	25	19.29	25	1.00	0.89	25	39.40	25	1.00	1.00	0.490
	12	25	19.31	25	0.80	0.70	25	38.25	25	1.00	1.00	0.505
	13	25	21.82	25	0.96	0.90	25	43.15	25	1.00	1.00	0.506
	14	25	19.32	25	0.84	0.75	25	40.58	25	1.00	1.00	0.476
	15	25	24.94	25	1.00	0.98	25	38.44	25	1.00	1.00	0.649
	16	25	18.93	25	0.84	0.76	25	38.08	25	1.00	1.00	0.497
	17	25	22.80	25	1.00	0.93	25	37.77	25	1.00	1.00	0.604
	18	25	20.91	25	1.00	0.98	25	39.95	25	1.00	1.00	0.523
	19	25	25.92	25	1.00	1.00	25	42.85	25	1.00	1.00	0.605
	20	25	19.73	25	0.84	0.77	25	40.17	25	1.00	1.00	0.491
	21	25	20.56	25	0.96	0.85	25	41.01	25	1.00	1.00	0.501
	22	25	20.42	25	1.00	0.98	25	39.05	25	1.00	1.00	0.523
	23	25	24.17	25	1.00	0.91	25	45.25	25	1.00	1.00	0.534
	24	25	26.93	25	0.96	0.89	25	42.35	25	1.00	1.00	0.636
	25	25	24.25	25	1.00	1.00	25	45.01	25	1.00	1.00	0.539
	26	25	20.13	25	0.76	0.66	25	40.07	25	1.00	1.00	0.502
	27	25	20.49	25	1.00	0.98	25	41.95	25	1.00	1.00	0.488
	28	25	19.23	25	1.00	0.99	25	36.33	25	1.00	1.00	0.529
	29	25	24.04	25	0.96	0.85	25	46.42	25	1.00	1.00	0.518
	30	25	20.38	25	0.72	0.61	25	37.58	25	1.00	1.00	0.542

(to be continued)

Supplemental Table 10. (continued)

Population No.	Plant No.	First florets No. of grains measured	First florets One-grain weight (mg)	First florets No. of grains sown	First florets GR	First florets GI	Second florets No. of grains measured	Second florets One-grain weight (mg)	Second florets No. of grains sown	Second florets GR	Second florets GI	GWR
F_4-3	31	25	23.70	25	1.00	0.86	25	45.64	25	1.00	1.00	0.519
	32	25	22.73	25	0.92	0.81	25	42.47	25	1.00	1.00	0.535
	33	25	21.95	25	0.84	0.77	25	40.94	25	1.00	1.00	0.536
	34	25	22.93	25	0.80	0.72	25	42.14	25	1.00	1.00	0.544
	35	25	22.45	25	0.88	0.74	25	44.02	25	1.00	1.00	0.510
	36	25	20.79	25	0.84	0.75	25	39.46	25	1.00	1.00	0.527
	37	25	23.51	25	0.88	0.80	25	43.39	25	1.00	1.00	0.542
	38	25	21.30	25	0.80	0.73	25	39.82	25	1.00	1.00	0.535
	39	25	23.98	25	0.96	0.81	25	44.66	25	1.00	1.00	0.537
	40	25	20.68	25	1.00	0.99	25	37.78	25	1.00	1.00	0.547
F_4 population derived from self-pollination of the F_3 plant 15502-13												
F_4-4	1	25	24.92	25	1.00	0.99	25	36.86	25	1.00	1.00	0.676
	2	25	19.29	25	1.00	0.92	25	34.61	25	1.00	1.00	0.557
	3	25	20.80	25	1.00	0.93	25	38.67	25	1.00	1.00	0.538
	4	25	23.88	25	1.00	0.99	25	39.12	24	1.00	1.00	0.610
	5	25	22.74	25	1.00	0.98	25	39.00	25	1.00	1.00	0.583
	6	25	24.56	25	1.00	0.98	25	41.02	25	1.00	1.00	0.599
	7	25	23.86	25	1.00	0.99	25	39.90	25	1.00	1.00	0.598
	8	25	20.05	25	1.00	0.95	25	36.54	25	1.00	1.00	0.549
	9	25	17.68	25	0.92	0.87	25	34.53	25	1.00	1.00	0.512
	10	25	19.97	25	1.00	0.91	25	35.55	25	1.00	1.00	0.562
	11	25	20.90	25	0.88	0.84	25	38.50	25	1.00	1.00	0.543
	12	25	21.22	25	0.88	0.87	25	38.40	25	1.00	1.00	0.553
	13	25	23.01	25	1.00	0.95	25	39.47	25	1.00	1.00	0.583
	14	25	27.36	25	1.00	0.99	25	40.74	25	1.00	1.00	0.672
	15	25	22.61	25	1.00	0.86	25	40.78	25	1.00	1.00	0.554
	16	25	19.50	25	1.00	0.99	25	33.36	25	1.00	1.00	0.584
	17	25	20.91	25	1.00	0.97	25	35.99	25	1.00	1.00	0.581
	18	25	21.36	25	1.00	0.97	25	37.68	25	1.00	1.00	0.567
	19	25	19.82	25	0.96	0.90	25	37.79	23	1.00	1.00	0.525
	20	25	17.19	25	1.00	0.92	25	30.66	25	1.00	1.00	0.561
	21	25	20.63	25	1.00	0.98	25	38.42	25	1.00	1.00	0.537
	22	25	21.03	25	1.00	0.96	25	39.18	25	1.00	1.00	0.537
	23	25	22.49	24	1.00	0.97	25	34.92	25	1.00	1.00	0.644
	24	25	16.15	25	1.00	0.95	25	32.32	25	1.00	1.00	0.500

Supplemental Table 11. One-grain weights, GWR, GR, and GI values of the first and second floret grains in two-grained spikelets of two F_6 populations and an F_5 population derived from low-GI and high-GI F_4 plants, respectively (sown on August 24, 2018)

		First floret grains					Second floret grains					
Population No.	Plant No.	No. of grains measured	One-grain weight (mg)	No. of grains sown	GR	GI	No. of grains measured	One-grain weight (mg)	No. of grains sown	GR	GI	GWR
F_6 populations derived from the low-GI F_4 plant, F_4-1a-6												
F_6-1	1	25	19.18	25	0.32	0.31	25	40.79	25	1.00	1.00	0.470
	2	25	21.49	25	0.08	0.07	25	38.70	24	1.00	1.00	0.555
	3	10	16.93	9	0.22	0.19	10	30.07	10	1.00	1.00	0.563
	4	15	17.67	15	0.13	0.10	15	32.26	15	1.00	1.00	0.548
	5	25	19.49	25	0.08	0.06	25	37.51	25	1.00	1.00	0.520
	6	25	18.43	25	0.16	0.13	25	33.70	23	1.00	1.00	0.547
	7	25	18.06	25	0.08	0.06	25	36.84	25	1.00	1.00	0.490
	8	25	20.95	25	0.12	0.07	25	40.97	25	1.00	1.00	0.511
	9	25	21.37	25	0.12	0.12	25	39.86	25	1.00	1.00	0.536
	10	25	20.00	25	0.04	0.03	25	40.78	25	1.00	1.00	0.490
	11	25	18.64	25	0.16	0.11	25	34.84	25	1.00	0.99	0.535
	12	20	19.80	20	0.00	0.00	20	34.50	20	1.00	1.00	0.574
	13	20	19.09	20	0.10	0.09	20	37.86	20	1.00	1.00	0.504
	14	17	18.34	17	0.00	0.00	17	35.05	17	1.00	1.00	0.523
	15	18	20.94	18	0.00	0.00	18	39.84	18	1.00	1.00	0.526
	16	25	18.45	25	0.20	0.19	25	35.24	25	1.00	1.00	0.524
	17	25	18.65	25	0.20	0.18	25	38.68	25	1.00	1.00	0.482
	18	25	21.00	25	0.24	0.22	25	39.82	25	1.00	1.00	0.527
	19	25	20.57	25	0.04	0.03	25	37.88	25	1.00	1.00	0.543
	20	25	18.37	25	0.04	0.02	25	36.38	25	1.00	1.00	0.505
	21	25	20.94	25	0.16	0.14	25	41.90	25	1.00	1.00	0.500
	22	25	22.02	25	0.16	0.12	25	39.84	25	1.00	1.00	0.553
F_6-2	1	20	21.31	20	0.15	0.11	20	34.65	20	1.00	1.00	0.615
	2	25	20.18	25	0.00	0.00	25	37.22	25	1.00	1.00	0.542
	3	25	17.28	25	0.08	0.06	25	32.02	25	1.00	1.00	0.540
	4	25	16.28	25	0.16	0.13	25	27.91	25	1.00	1.00	0.583
	5	25	16.33	24	0.29	0.26	25	35.13	25	1.00	1.00	0.465
	6	25	17.06	25	0.00	0.00	25	34.12	24	1.00	1.00	0.500
	7	17	17.56	15	0.07	0.07	17	33.75	17	1.00	1.00	0.520
	8	25	19.69	25	0.08	0.06	25	38.52	25	1.00	1.00	0.511
	9	25	18.44	25	0.08	0.07	25	31.57	24	1.00	1.00	0.584
	10	25	16.41	25	0.04	0.03	25	32.52	25	1.00	1.00	0.505
	11	25	17.03	25	0.28	0.26	25	33.38	25	1.00	1.00	0.510
	12	25	16.98	24	0.08	0.08	25	33.27	25	1.00	1.00	0.510
	13	25	18.60	25	0.20	0.17	25	37.18	25	1.00	1.00	0.500
	14	25	17.01	25	0.04	0.03	25	34.38	25	1.00	0.99	0.495
	15	25	15.05	25	0.08	0.05	25	32.36	25	1.00	0.99	0.465

(to be continued)

Supplemental Table 11. (continued)

Population No.	Plant No.	First floret grains: No. of grains measured	One-grain weight (mg)	No. of grains sown	GR	GI	Second floret grains: No. of grains measured	One-grain weight (mg)	No. of grains sown	GR	GI	GWR
F_6-2	16	18	18.24	18	0.33	0.27	18	32.43	14	1.00	1.00	0.563
	17	20	17.11	20	0.20	0.20	20	31.77	20	1.00	1.00	0.538
	18	25	16.58	25	0.12	0.08	25	30.91	25	1.00	1.00	0.536
	19	25	16.78	25	0.04	0.03	25	34.78	24	1.00	1.00	0.482
	20	20	16.63	19	0.11	0.09	20	34.09	20	1.00	1.00	0.488
	21	25	17.02	25	0.08	0.08	25	32.93	25	1.00	1.00	0.517
	22	25	16.15	25	0.16	0.14	25	32.30	25	1.00	1.00	0.500
F_5 population derived from the high-GI F_4 plant, F_4-4-21												
F_5-1	1	25	20.45	25	1.00	1.00	25	35.56	21	1.00	1.00	0.575
	2	25	21.81	25	0.92	0.92	25	36.39	25	1.00	1.00	0.599
	3	25	18.31	25	0.84	0.82	25	32.98	25	1.00	1.00	0.555
	4	25	23.98	25	1.00	0.99	25	36.29	25	1.00	1.00	0.661
	8	25	24.01	25	1.00	1.00	25	38.38	25	1.00	1.00	0.626
	9	25	19.42	25	0.92	0.91	25	34.80	25	1.00	1.00	0.558
	10	25	18.34	25	0.96	0.94	25	33.55	25	1.00	1.00	0.547
	12	25	19.85	25	0.92	0.91	25	32.93	25	1.00	1.00	0.603
	13	25	19.78	25	0.88	0.81	25	33.30	25	1.00	1.00	0.594
	14	25	23.58	25	1.00	1.00	25	37.31	25	1.00	1.00	0.632
	15	25	21.32	25	1.00	1.00	25	32.28	25	1.00	1.00	0.660
	16	25	19.36	25	1.00	0.97	25	33.90	25	1.00	1.00	0.571
	17	25	21.01	25	1.00	0.99	25	35.63	25	1.00	1.00	0.590
	18	25	20.09	25	0.84	0.84	25	39.04	25	1.00	0.99	0.514
	19	25	20.56	25	1.00	1.00	25	36.42	25	1.00	1.00	0.564
	20	25	19.18	25	0.84	0.84	25	36.28	25	1.00	0.99	0.529
	21	25	21.68	25	1.00	1.00	25	36.21	25	1.00	1.00	0.599
